# Novel Selective IDO1 Inhibitors with Isoxazolo[5,4-*d*]pyrimidin-4(5*H*)-one Scaffold

**DOI:** 10.3390/ph14030265

**Published:** 2021-03-15

**Authors:** Ana Dolšak, Tomaž Bratkovič, Larisa Mlinarič, Eva Ogorevc, Urban Švajger, Stanislav Gobec, Matej Sova

**Affiliations:** 1 Faculty of Pharmacy, University of Ljubljana, Aškerčeva 7, 1000 Ljubljana, Slovenia; ana.dolsak@ffa.uni-lj.si (A.D.); tomaz.bratkovic@ffa.uni-lj.si (T.B.); larisa.mlinaric@gmail.com (L.M.); eva.ogorevc@ijs.si (E.O.); urban.svajger@ztm.si (U.Š.); stanislav.gobec@ffa.uni-lj.si (S.G.); 2 Blood Transfusion Centre of Slovenia, Šlajmerjeva 6, 1000 Ljubljana, Slovenia

**Keywords:** indoleamine 2,3-dioxygenase 1, selective inhibitors, isoxazolo[5,4-*d*]pyrimidin-4(5*H*)-one, immunomodulation, cancer

## Abstract

Indoleamine 2,3-dioxygenase 1 (IDO1) is a promising target in immunomodulation of several pathological conditions, especially cancers. Here we present the synthesis of a series of IDO1 inhibitors with the novel isoxazolo[5,4-*d*]pyrimidin-4(5*H*)-one scaffold. A focused library was prepared using a 6- or 7-step synthetic procedure to allow a systematic investigation of the structure-activity relationships of the described scaffold. Chemistry-driven modifications lead us to the discovery of our best-in-class inhibitors possessing *p*-trifluoromethyl (**23**), *p*-cyclohexyl (**32**), or *p*-methoxycarbonyl (**20**, **39**) substituted aniline moieties with IC_50_ values in the low micromolar range. In addition to hIDO1, compounds were tested for their inhibition of indoleamine 2,3-dioxygenase 2 and tryptophan dioxygenase, and found to be selective for hIDO1. Our results thus demonstrate a successful study on IDO1-selective isoxazolo[5,4-*d*]pyrimidin-4(5*H*)-one inhibitors, defining promising chemical probes with a novel scaffold for further development of potent small-molecule immunomodulators.

## 1. Introduction

Tryptophan (Trp) is the least abundant essential amino acid with significant and various roles in the human body. Since its only source is dietary intake, its plasma concentration must be precisely regulated. Most of the Trp is metabolized via the kynurenine (Kyn) pathway, with only 5% being used for protein synthesis, or converted into serotonin [1]. The first and rate-limiting step of Trp conversion to *N*-formylkynurenine, which spontaneously hydrolyses to Kyn, is catalyzed by one of three isoforms of heme-containing dioxygenases (indoleamine 2,3-dioxygenase 1 (IDO1), indoleamine 2,3-dioxygenase 2 (IDO2), and tryptophan dioxygenase (TDO)) [2]. TDO, which was first described in 1936 [3], is primarily responsible for the metabolism of dietary Trp and is therefore mainly located in the liver [4]. In contrast, IDO2 has only been known for 13 years, and its biological role has not yet been clarified in detail [5,6]. Among the three isoforms, the most studied one is IDO1, which is widely distributed in many tissues. It has been reported that its overexpression in various cancers correlates with poor disease prognosis [7]. IDO1, TDO, and IDO2 have been studied primarily for their pivotal role in cancer evasion and immunosuppression [8,9,10], yet Kyn and its metabolites are involved in several other pathological conditions (e.g., viral and bacterial infections-related immunosuppression [11], neurological disorders [12,13], and osteoporosis [14]).

**Figure 1:**
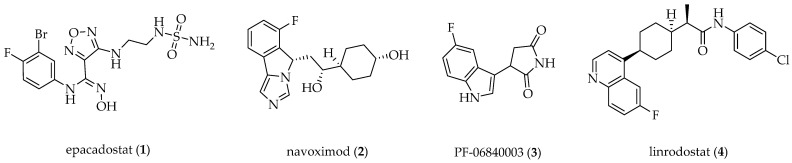
Structures of representative IDO1 inhibitors.

In the last decade, a considerable number of IDO1 inhibitors have been developed (thoroughly reviewed in our recent paper [15]), possessing several different scaffolds, i.e., hydroxyamidine (e.g., epacadostat **1**) [16,17], phenylimidazole (e.g., navoximod **2**) [18], indole (e.g., PF-06840003 **3**) [19], and 1-(4-arylcyclohex-1-yl)propanamide (e.g., linrodostat **4**) [20,21,22], which are presented in Figure 1. To date, however, none of the IDO inhibitors have successfully completed clinical trials. A design of dual or multiple inhibitors of isoforms could contribute to the improved clinical efficacy of Kyn pathway blockade [23]. A detailed discussion on differences and selectivity determinants of IDO1, IDO2, and TDO is presented in our recent review paper [15]. Compensatory up-regulation of IDO2 and TDO as a consequence of IDO1 inhibition is possible; however, the exact mechanisms have not been elucidated yet. Multitargeting has not been extensively researched yet, so little is known about possible benefits and/or negative side effects. One of the possible drawbacks of multitargeting could be (potentially toxic) accumulation of Trp if both IDO1 and TDO were inhibited since they represent the two enzymes physiologically important for Trp metabolism. Taken together, until some new revolutionary findings of multitargeting are uncovered, selective inhibition of IDO1 is still of major importance.

**Scheme 1:**
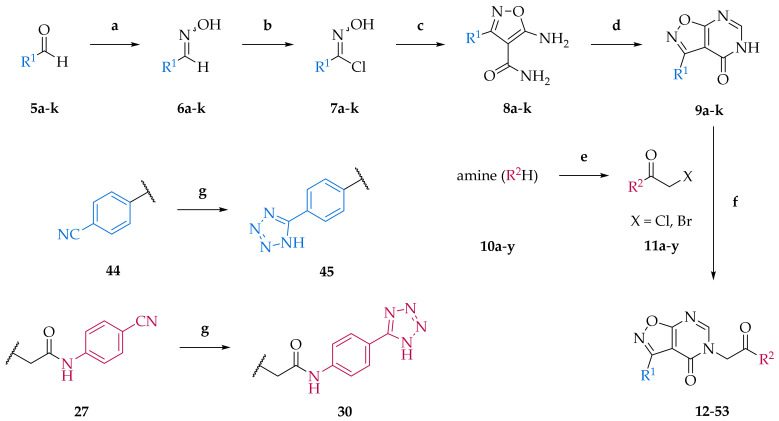
Synthetic procedure for the preparation of isoxazolo[5,4-*d*]pyrimidin-4(5*H*)-one derivatives. Reagents, conditions and yields for each step: (a) hydroxylamine hydrochloride, THF/EtOH/H_2_O (2/5/1, *v*/*v*), r.t., 30 min; 71–98%; (b) NCS, DMF, r.t., 18 h; (c) Procedure I: (i) NaOEt/EtOH, 2-cyanoacetamide, 50 °C; (ii) dropwise addition of hydroxyimidoyl chlorides **7a**-**k** at 0 °C; (iii) r.t., 30 min; then reflux, 18 h; or procedure II: (i) NaH, anh. DMF, 2-cyanoacetamide, r.t., 2 h; (ii) 70 °C, 18 h; 6–71%; (d) triethyl orthoformate, Ac_2_O, reflux, 18 h; 8–85%; (e) Procedure III: (i) chloroacetyl chloride, glacial acid, 10–15 °C, 30 min; (ii) NaOAc, H_2_O, rt, 1h; 24–100%; or Procedure IV: chloroacetyl chloride, DCM, 10–15 °C, 2 h; 48–100%; or Procedure V: bromoacetyl bromide in 1,4-dioxane, DMF, r.t., 18 h; 61–71%; (f) (i) R^2^COCH_2_X, NaH, BTEAC, Ar; (ii) KI, anh. MeCN, r.t., 18 h; 6–82%; (g) (i) NaN_3_, ZnBr_2_, H_2_O, reflux, 18 h; (ii) NaOH, 1 h; 15–80%. Abbreviations: THF, tetrahydrofuran; NCS, *N*-chlorosuccinimide; DMF, *N*,*N*-dimethylformamide; r.t., room temperature; DCM, dichloromethane; BTEAC, benzyltriethylammonium chloride.

Herein, we report the synthesis of selective IDO1 inhibitors with novel isoxazolo[5,4-*d*]pyrimidin-4(5*H*)-one scaffold identified in our hit compound **12** [24]. The aim of this study was to develop the appropriate synthetic procedures, synthesize a focused library of isoxazolo[5,4-*d*]pyrimidin-4(5*H*)-ones along with **12** (Scheme 1, Table 1), explore the structure-activity relationships (SARs) of synthesized compounds and determine the selectivity among three enzyme isoforms, i.e., IDO1, TDO, and IDO2. For the determination of inhibitory potency, hIDO1 was expressed in *Escherichia coli* and purified by immobilized-metal affinity chromatography (IMAC). The selectivity of the compounds for hIDO1 was evaluated by comparative determination of inhibitory potencies against hIDO2 and hTDO using newly established highly sensitive fluorescence assays. The fluorescence-based assay for the determination of IDO1 activity was established in 2013 [25]; however, it has not been used for TDO and IDO2 yet.

## 2. Results and Discussion

### 2.1. Chemistry

First of all, the appropriate synthetic procedures for the synthesis of the main isoxazolo[5,4-*d*]pyrimidin-4(5*H*)-one scaffold were established. To examine the chemical space around this scaffold and investigate the SAR, a focused library of substituted isoxazolo[5,4-*d*]pyrimidin-4(5*H*)-ones was prepared. Modifications were implemented in two positions, namely R^1^ and R^2^ (as depicted in Scheme 1), where different aromatic rings (R^1^) and amines (R^2^H) were introduced. Isoxazolo[5,4-*d*]pyrimidin-4(5*H*)-one scaffold was prepared in four synthetic steps according to previously published procedures [26,27,28,29]. Some steps were also optimized during the synthesis. Substitutions in positions R^1^ and R^2^ were introduced in the first or the last step of the synthesis, respectively.

In the first two steps, arylaldehydes (**5a**–**k**) were subjected to hydroxylamine hydrochloride to yield corresponding oximes (**6a**–**k**). The following treatment with *N*-chlorosuccinimide (NCS) afforded hydroxyimidoyl chlorides (**7a**–**k**). These nitrile oxide precursors, which were used immediately without further purification, easily underwent 1,3-dipolar cycloaddition in the third synthetic step. The published procedure [26] employed NaOEt as a strong base to convert 2-cyanoacetamide to a nucleophilic moiety. However, this required additional time to prepare fresh NaOEt in situ and in some cases low yields in the following reaction were obtained. Thus, we optimized and shortened reaction time by using NaH in DMF instead of NaOEt. The obtained 3-aryl-5-aminoisoxazole-4-carboxamides (**8a**–**k**) were further cyclized with triethyl orthoformate to yield aryl-isoxazolo[5,4-*d*]pyrimidin-4(5*H*)-ones (**9a**–**k**). Concurrently, 2-chloro or 2-bromoacetamides (**11a**–**y**) were prepared via three different procedures, based on their physicochemical properties (e.g., solubility, reactivity). In the last step, isoxazolo[5,4-*d*]pyrimidin-4(5*H*)-one core was subjected to *N*-alkylation to yield the final compounds **12**–**53**. To improve the reaction, benzyltriethylammonium chloride (BTEAC) as a phase-transfer catalyst, and potassium iodide (conversion to a better leaving group) were used. The compounds with cyano substituent (i.e., **27**, **44**) were reacted with NaN_3_ in the presence of ammonium chloride to prepare the corresponding tetrazolo substituted derivatives (i.e., **30**, **45**). Forty-two final compounds (**12**–**53**) were prepared via the described synthetic procedures. For twenty-five of them, modifications in the form of various 2-halogenoacetamides were introduced in the last step, ranging from aliphatic amines to differently substituted anilines. Instead of 4-fluorobenzaldehyde, which was present in compound **12**, several other arylaldehydes were introduced in the first synthetic step to yield additional sixteen final compounds for biological evaluation.

### 2.2. hIDO1 Expression

**Figure 2:**
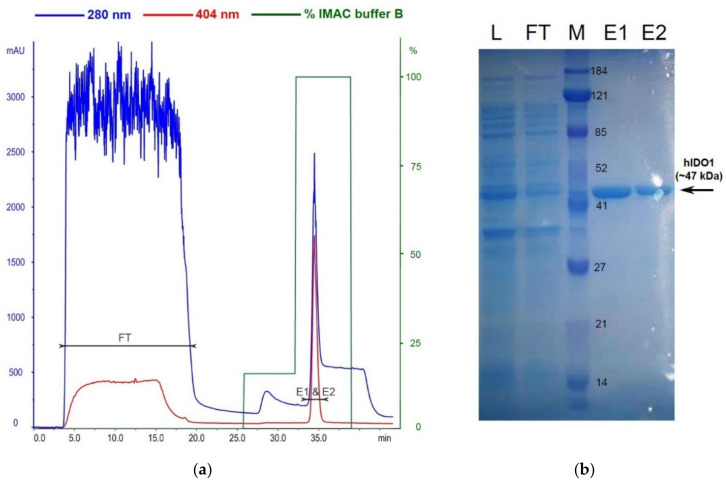
Recombinant hIDO1 purification: (**a**) IMAC chromatogram; (**b**) SDS-PAGE analysis of purified protein: L—bacterial lysate; FT—flow-through; M—protein standard; E1 & E2—imidazole-eluted fractions.

Expression and purification of hIDO1 was essentially performed as previously described [20], with minor modifications. Relatively high cell density prior to induction of recombinant protein expression was necessary for optimal yield. The glucose present in the induction medium suppresses the *lacUV5* promoter, allowing gentle induction, providing it is gradually consumed by the expression strain growing in the buffered medium at low temperature. The induction medium was supplemented with 5-aminolevulinic acid (heme precursor) to boost heme synthesis. The addition of strong reducing agents (i.e., tris(2-carboxyethyl)phosphine (TCEP) and 1,4-dithiothreitol) in all buffers during hIDO1 purification was essential to ensure high heme occupancy by preventing oxidation of cysteine residues (i.e., formation of disulfide bonds), thus retaining the enzyme in its native conformation. Single-step chromatographic purification afforded highly pure hIDO1 as confirmed by SDS-PAGE electrophoresis of eluted material (Figure 2).

### 2.3. Determination of Inhibitory Potency

All the synthesized compounds were evaluated for their inhibitory potencies against hIDO1 (Table 1) according to the published protocol [25]. Our synthesized starting compound **12** exhibited IDO1 inhibitory activity of 50 μM. To investigate SAR, two series of compounds were prepared, the first focusing on amine substitution, whereas the second series possessed various modifications of 4-fluorophenyl fragment. Regarding the first series, the inhibition of IDO1 was only achieved with bulkier substituents, which can be clearly seen by the active compound **15** (IC_50_ value of 88 µM), contrary to significantly less potent **13** and **14** with smaller amine substituents. Concerning aromatic amines, the appropriate substitution on aniline is mandatory for the potency, since **16** and **17** with aniline and *p*-fluoroaniline, respectively, showed only weak inhibition. Our aim to identify the preferred position and nature of aniline substitution encouraged us to prepare several aniline-substituted isomers (compounds **17**–**34**, Scheme 1, Table 1) On the trio of isomers **18**, **19** and **20**, substituted with methoxycarbonyl group, the *ortho* substitution corresponding to **18** was found less appropriate in comparison with *para* in **20**, which emerged as superior in potency with IC_50_ of 27 µM. The following observation was verified and confirmed with two additional sets of *meta* vs. *para* isomers (**22** vs. **23** and **24** vs. **25**). In accordance with obtained results, we therefore focused solely on *para* aniline substitution to further explore the chemical space around the aniline fragment. The introduction of an additional methylene group in the aniline moiety affording *p*-methoxycarbonyl substituted benzylamine fragment resulted in a decrease of inhibitory potency (**21** compared to **20**).

Most of the active compounds possess hydrogen bond acceptors incorporated in the *p-*aniline substituent. The only exceptions are compounds **22** and **23** with trifluoromethyl group, as well as **31** and **32** possessing lipophilic fragments (Table 1). The introduction of polar dimethylamino group (as in **28**) or acidic tetrazol moiety (as in **30**), which mimics the carboxyl group led to weak inhibition or inactive compounds, respectively. Substitution with methylsulfonyl (**26**), cyano (**27**), 3,4,5-trimethoxy (**34**), or longer butoxy group (**33**) also resulted in inactive compounds. Interestingly, the attachment of isoxazolo ring in compound **29** on the *para* position of aniline afforded a potent IDO1 inhibitor with IC_50_ value of 30 µM.

The next step was the replacement of the aniline ring with the naphthalene or tetrahydroisoquinoline moiety (Table 1, compounds **35**, **36**, and **37**). 2-naphthyl and tetrahydroquinoline substitutions (as in **12**) were more appropriate compared to 1-naphthyl and tetrahydroisoquinoline, respectively. With the second series of compounds, we tried to investigate the chemical space around the substitution at the other (left) side of the selected isoxazolo[5,4-*d*]pyrimidin-4(5*H*)-one scaffold (Table 2). Different modifications of 4-fluorophenyl fragment were introduced in the first step of the synthesis (Scheme 1), whereas the tetrahydroquinoline moiety as the R^2^ fragment, originating from **12**, was preserved in most of the compounds. Interestingly, in combination with tetrahydroquinoline as R^2^ substituent, the optimal R^1^ substituent was found to be the original 4-fluorophenyl fragment since none of the other aryl analogs seemed to improve the potency of **12**. However, the replacement of tetrahydroquinoline with *p*-nitroaniline or methyl ester of *p*-aminobenzoic acid in the R^2^ position also afforded potent inhibitors. Inhibitory potencies of **48** and **49**, possessing 3,4-dihalogenophenyl fragment, and **40** with phenyl fragment were comparable to their 4-fluorophenyl analog **25**. Another heteroaromatic substituent in the form of thiophene was found active, when combined with *p-*nitroaniline moiety as R^2^. Two compounds with different thiophene orientations were prepared, both **51** with tiophen-2-yl fragment retained the potency (IC_50_ of 53 µM) and **53** with tiophene-3-yl fragment (IC_50_ of 40 µM) exhibited superior potency than their analog **25**. Compound **39** with phenyl substituent as R^1^ and methyl ester of *p*-aminobenzoic acid in the R^2^ position retained a similar potency to its 4-fluorophenyl analog **20**. Taken together, the R^1^ position is more liable to modifications, when combined to *p*-substituted anilines with hydrogen bond acceptors as R^2^ substituents.

Additionally, to determine IDO1 selectivity, all final compounds were assayed for their inhibitory potencies towards hIDO2 and hTDO. For this purpose, two new protocols were developed based on the published fluorescence assay for hIDO1 [25]. Sodium ascorbate, methylene blue, and bovine catalase were used in the same concentrations as for hIDO1. 50 mM phosphate buffers with pH values of 7.5 and 7.0 were used for hIDO2 and hTDO, respectively, as has been already mentioned before [30,31,32]. First, the activities of hIDO2 and hTDO were determined at four different concentrations. Enzyme concentrations of 600 nM and 200 nM for hIDO2 and hTDO, respectively, exhibited sufficient enzyme activity that could be measured in the assay. Additionally, the effect of DMSO concentration on enzyme activity was investigated. Up to 5%, *v*/*v* of DMSO did not show any effect on the assay performance. The optimal hIDO2 and hTDO assay concentrations of the substrate *L*-Trp were determined to be 4 mM and 500 μM, respectively. To validate both assays, the potency of epacadostat (**1**) was determined, with IC_50_ values of 580 nM and 9620 nM for hIDO2 and hTDO, respectively, corresponding to those published in the literature (see Figure S1) [32,33]. All compounds tested were found to be selective for hIDO1, since none of them exhibited significant inhibition of hIDO2 or hTDO.

**Figure 3:**
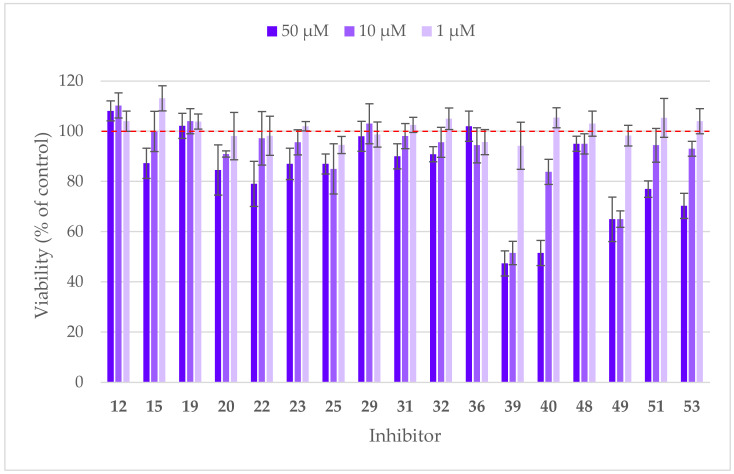
Cytotoxicity of active compounds on Hep G2 cell line was evaluated at three different concentrations. Data are represented as mean values ± standard deviation.

All active compounds were additionally evaluated on a cellular level, starting with the determination of cytotoxicity on Hep G2 cell line using the MTS assay (Figure 3), where inner salt of [3-(4,5-dimethylthiazol-2-yl)-5-(3-carboxymethoxyphenyl)-2-(4-sulfophenyl)-2*H*-tetrazolium is converted to formazan product, its quantity is directly proportional to the number of living cells. Since the majority of compounds were only partially soluble in a cell medium at concentrations above 100 μM, their effect on cell viability was measured at 50 μM, 10 μM, and 1 μM. Most of the compounds displayed little or no cytotoxicity against Hep G2 cells at 50 μM. Compounds **39** and **49** significantly impacted cell viability even at 10 μM, especially **39** was thus not considered as a perspective inhibitor despite its potent IDO1 inhibition in an enzymatic assay. The most promising compound **23** was further evaluated in the cell-based IDO1 inhibition assay on HeLa cells, where no activity was detected, possibly due to poor physicochemical properties, such as solubility in the culture medium. Taken together, the results obtained from cellular tests reminded us of the necessity to consider the physicochemical properties of compounds even at the early stages of design.

**Figure 4:**
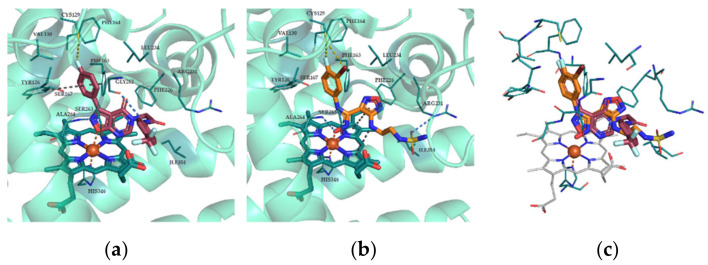
Comparison of predicted binding mode for **23**, crystal structure of IDO1 with **1** and superposition of **1** and **23**. (**a**) Compound **23** (purple) was docked into the active site of IDO1 (pdb code 5WN8, cyan) with protein ligand docking software GOLD 2020.0 (CCDC, UK). Possible interactions formed were additionally predicted with Protein-Ligand Interaction Profiler [35] and are: a coordinate bond (orange) with heme iron; H-bonds (blue) with Gly262 and Ala264; π-π stacking (grey) with Tyr126 and Phe163 and several hydrophobic interactions (not shown) with lipophilic milieu. 4-fluorophenyl ring of **23** was positioned similarly to 3-bromo-4-fluorophenyl ring of **1**; therefore, we additionally predicted another interaction (olive) between halogen and Cys129. (**b**) Crystal structure of **1** (orange) with IDO1 (cyan, 5WN8) with reported interactions: a coordinate bond (orange) with heme iron; H-bonds (blue) with Ala264 and Arg231; π-π stacking and cation-π interaction (both grey) between heme and **1**, and interaction between halogens of F and Br with Cys129. (**c**) Superposition of **1** (orange) and **23** (purple) shows similar binding into the active site of IDO1 (heme, grey; amino acid residues responsible for interactions, cyan).

To predict the binding mode of our inhibitors based on isoxazolo[5,4-*d*]pyrimidin-4(*5H*)-one scaffold we selected **23** as the most promising compound (since it showed potent hIDO1 activity with IC_50_ of 23 μM, no activity on hIDO2 and hTDO, and satisfying cytotoxicity profile) and docked it into the active site of IDO1 (Figure 4), where in addition to a coordinate bond between heme iron and the oxygen atom in the isoxazole ring, H-bonds, π-π stacking and hydrophobic interactions (Figure 4a) were also observed. To compare the binding mode of **23** to the one of the most potent inhibitors from clinical trials, the superposition of **23** and epacadostat (**1**) was also performed (Figure 4c). In accordance with interactions with Cys129 via fluorine and bromine atoms on the phenyl ring of **1**, which were observed in the literature [34], a similar interaction between fluorine atom on the phenyl ring of **23** and Cys129 can be predicted.

## 3. Materials and Methods

### 3.1. Chemistry and Chemical Characterization of Compounds

The reagents and solvents were purchased from commercial sources (Sigma-Aldrich, Acros Organics, Alfa Aesar, TCI) and used without further purification. Monitoring of the reactions was done by thin-layer chromatography on silica-gel plates (Merck DC Fertigplatten Kieselgel 60 GF_254_) and visualized under UV light and/or stained with the relevant reagents. Flash column chromatography was performed on Merck silica gel 60 (mesh size, 70–230), using the indicated solvents. Yields refer to the purified products and were not optimized. All of the melting points were determined on a Reichert hot-stage apparatus, and are uncorrected. ^1^H and ^13^C NMR spectra were recorded at 295 K in CDCl_3_, DMSO-*d_6_*, (CD_3_)_2_CO, or MeOD on a Bruker Avance III NMR spectrometer equipped with a Broadband decoupling inverse ^1^H probe. The coupling constants (*J*) are in Hz, and the splitting patterns are designated as s, singlet; br s, broad singlet; d, doublet; dd, double doublet; td, triple doublet; t, triplet; dt, double triplet; ddd, double of doublet of doublet; and m, multiplet. Mass spectra and high-resolution mass measurements were performed at the Faculty of Pharmacy, University of Ljubljana, on ADVION Expression CMSL mass spectrometer (Advion Inc., Ithaca, NY, USA) and Exactive^TM^ Plus Orbitrap mass spectrometer (Thermo Fisher Scientific Inc., Waltham, MA, USA), respectively. The analytical purities of the assayed compounds were ≥95%, unless stated otherwise, as determined by ^1^H NMR and HPLC analyses. HPLC analyses were run at 50 °C on a Thermo Scientific Dionex UltiMate 3000 System (Thermo Fisher Scientific Inc., Waltham, MA, USA) equipped with a quaternary pump and a multiple wavelength detector. An Agilent Extend-C18 column was used (4.6 mm × 150 mm, 3.6 μm), with a flow rate of 1.0 mL/min, injection volume of 5 μL, detection at 254 nm, and an eluent system of A, 0.1% aqueous TFA; B, CH3CN. The following gradients were applied: 0–12 min, 10% → 90% B; 12–14 min 90% B; 14–15 min, 90% → 10% B.

Analytical data of synthesized compounds as well as representative ^1^H and ^13^C NMR spectra are available in the Supplementary Materials related to this manuscript.

### 3.2. General Procedure for the Synthesis of Aldehyde Oximes (**6a**–**k**)

Appropriate aldehyde **5a**–**k** (1.0 equiv.) was added to a stirred solution of hydroxylamine × HCl (1.1 equiv.) in THF-EtOH-H_2_O (2:5:1, *v*/*v*), with the mixture being stirred at room temperature for approximately 30 min. THF and EtOH were then evaporated under reduced pressure. The remaining residue was extracted with Et_2_O, brine, and dried over anhydrous Na_2_SO_4_. Solvents were removed under reduced pressure to yield the product (**6a**–**k**), which was used in the next step without further purification.

### 3.3. General Procedure for the Synthesis of N-Hydroxyimidoyl Chlorides (**7a**–**k**)

Appropriate aldehyde oxime (**6a**–**k**) (1.0 equiv.) and NCS (1.0 equiv.) were dissolved in anhydrous DMF, then the reaction mixture was stirred at room temperature overnight (16 h). The next day, the mixture of n-Hex/Et_2_O (2:1, *v*/*v*) was added and extracted with H_2_O. The organic phase was washed with brine, dried over anhydrous Na_2_SO_4_, and the solvents were removed under reduced pressure to yield the *N*-hydroxyimidoyl chloride (**7a**–**k**), which was immediately used in the next step without any purification.

### 3.4. General Procedure for the Synthesis of 5-Aminoisoxazole-4-Carboxamides (**8a**–**k**)

#### 3.4.1. Procedure I

A fresh solution of NaOEt in EtOH, prepared at room temperature from sodium (1.5 equiv.) and absolute EtOH (~40 equiv.), was added dropwise to a stirred solution of 2-cyanoacetamide (1.0 equiv.) in absolute EtOH at 50 °C. The resulting suspension was then cooled in an ice bath, and the solution of *N*-hydroxyimidoyl chloride (**7a**–**k**) (1.0 equiv.) in EtOH was added dropwise. The mixture was stirred for an additional 30 min at 0 °C and then refluxed for 18 h. After completion of the reaction, EtOH was removed under reduced pressure. If possible, the resulting solid was washed with H_2_O and recrystallized from MeOH to yield a crystalline solid (**8a**–**k**). Otherwise, the solid was dissolved in EtOAc, washed with brine, and dried over anhydrous Na_2_SO_4_. EtOAc was removed under reduced pressure and the remaining solid was purified using flash column chromatography with gradient EtOAc/n-Hex as an eluent.

#### 3.4.2. Procedure II

NaH (1.2 equiv.) was added to a stirred solution of 2-cyanoacetamide (1.0 equiv.) in anhydrous DMF. After 15 min, a solution of appropriate *N*-hydroxyimidoyl chloride (**7a**–**k**) in anhydrous DMF was added dropwise. The reaction mixture was stirred under an inert atmosphere for 2 h at room temperature and then at 70 °C for 18 h. After completion of the reaction H_2_O was added and extracted with EtOAc. Combined organic phases were washed with brine, dried over anhydrous Na_2_SO_4_, and the solvents were removed under reduced pressure to yield 5-aminoisoxazole-4-carboxamides (**8a**–**k**), which were used in the next step without any purification.

### 3.5. General Procedure for the Synthesis of Isoxazolo[5,4-d]pyrimidin-4(5H)-ones (**9a**–**k**)

Isoxazolo[5,4-*d*]pyrimidin-4(5*H*)-ones (**9a**–**k**) were synthesized from appropriate 5-aminoisoxazole-4-carboxamide (**8a**–**k**) (1.0 equiv.) and triethylorthoformate (1.0 equiv.) dissolved in Ac_2_O and refluxed for 18 h. After completion, the reaction mixture was cooled down and stored in the fridge long enough for the precipitate to form, which was then collected by filtration and used in the next step without further purification.

### 3.6. General Procedures for the N-Acylation (Preparation of Alkyl Halogenides **11a**–**y**)

#### 3.6.1. Procedure III

To a solution of appropriate aromatic amine **10d**–**e**, **10i**–**y** (1.0 equiv.) in glacial acid (14.0 equiv.) chloroacetyl chloride (1.3 equiv.) was added dropwise. The reaction mixture was stirred at 10–15 °C for 30 min. After the addition of sodium acetate (3.7 equiv.) solution in H_2_O, the suspension was stirred at room temperature for 1.5 h. If the precipitate was formed, it was collected by filtration and used in the next step without further purification. Otherwise, EtOAc was added to the reaction mixture and extracted with H_2_O, brine, and dried over anhydrous Na_2_SO_4_. Then the solvents were evaporated under reduced pressure to yield a brown oily residue.

#### 3.6.2. Procedure IV

To a solution of aliphatic amine **10a**–**c** (1.0 equiv.) in DCM, chloroacetyl chloride (1.3 equiv.) was added dropwise. The reaction mixture was stirred at 10–15 °C for 2 h. After completion, the reaction mixture was extracted with saturated NaHCO_3_ and brine. The organic phase was dried over anhydrous Na_2_SO_4_ and the solvents were removed under reduced pressure to yield an oily residue.

#### 3.6.3. Procedure V

To a solution of appropriate aromatic amine **10f**–**h** (1.0 equiv.) in anhydrous DMF on the ice bath and under argon atmosphere bromoacetyl bromide (1.1 equiv.) in 1,4-dioxane (6 mL) was added dropwise. The reaction mixture was then stirred overnight at room temperature. The resulting precipitate was collected by filtration and used in the next step without further purification.

### 3.7. General Procedure for the Synthesis of Final Compounds (**12**–**53**)

To a solution of appropriate isoxazolo[5,4-*d*]pyrimidin-4(5*H*]-one (**9a**–**k**) (1.0 eqiv.) in anhydrous MeCN (10 mL) and anhydrous DMF (3 mL), NaH (60% dispersion in mineral oil, 1.7 equiv.) and BTEAC (10%, m/m) were added and the reaction mixture was stirred at 0 °C for 15 min. KI (1.1 equiv.) and the selected alkyl halogenide (**11a**–**y**, 1.3 equiv.) were added at room temperature and the reaction mixture was stirred for 18 h. After completion, the solvent was removed under reduced pressure, and the product was purified by flash column chromatography with EtOAc/n-Hex as the eluent.

### 3.8. Cloning, Expression and Isolation of rhIDO1

Synthetic codon-optimized gene encoding human IDO1 residues 6-403 His-tagged at the N-terminus (gBlock, IDT) was PCR amplified and subcloned into pET28 plasmid between the *Nco*I and *Xho*I restriction sites. A single bacterial colony of *Escherichia coli* NiCo21 harboring the expression vector was inoculated into 10 mL TB medium with 30 µg/mL kanamycin and the culture was grown overnight at 37 °C with vigorous shaking. The next day, the culture was diluted 1:100 in 200 mL TB medium with 30 µg/mL kanamycin and grown at 37 °C until OD_600_ reached ~2.5, at which point the cells were harvested by centrifugation and resuspended in 500 mL pre-warmed medium composed of 1 × M9 salts, 0.5% (wt/vol) D-glucose, 0.5% (wt/vol) casamino acids, 1× trace minerals, 1 mM MgSO_4_, 0.3 mM CaCl_2_, 1 µg/mL thiamine, 1 µg/mL biotin, 100 µg/mL L-tryptophan, 30 µg/mL kanamycin, and 1 mM aminolevulinic acid. The culture in a 3L baffled flask was shaken at 250 rpm at 37 °C for 45 min, and cooled on ice for 15 min. The chilled culture was supplemented with 0.5 mM isopropyl β-D-1-thiogalactopyranoside to induce recombinant protein expression, and incubated at 20 °C for 28 h, shaking at 250 rpm. The cells were harvested by centrifugation, washed with cold TBS buffer, and frozen at −80 °C.

The frozen cell paste was suspended in 10 volumes of cold IMAC buffer A (50 mM Tris·HCl, pH 7.4; 300 mM KCl, 25 mM imidazole, 5% glycerol, 1 mM TCEP) and disrupted by sonication (30% amplitude, 5 s pulses followed by 10 s pause) over 2 min. The lysate was supplemented with 10 µg/mL DNase I, incubated on ice for 20 min, and centrifuged at 10,000× *g* for 10 min at 4 °C. The supernatant was loaded on a 1 mL HiTrap IMAC column connected to ÄKTAexplorer 10 FPLC system (GE Healthcare, Wauwatosa, WI, USA) pre-equilibrated with IMAC buffer A at 0.5 mL/min. Absorbance was monitored at 280 nm and 404 nm. The column was washed at 1 mL/min with 15 mL IMAC buffer A, followed by 10 mL 87.3% IMAC buffer A + 12.7% IMAC buffer B (50 mM Tris·HCl, pH 7.4; 300 mM KCl, 300 mM imidazole, 5% glycerol, 1 mM TCEP), and the intensely red recombinant protein was eluted with IMAC buffer B. Eluates were immediately supplemented with 2 mM EDTA, combined and concentrated to 1 mL by ultrafiltration. Elution buffer was exchanged for 20 mM Tris·HCl, pH 8.0; 150 mM NaCl, 1 mM EDTA, 1 mM TCEP using a 5 mL HiTrap desalting column (GE Healthcare, Wauwatosa, WI, USA). The final rhIDO1 solution (3.5 mg/mL) was supplemented with 2 mM dithiotreitol, aliquoted and frozen at −80 °C. SDS-PAGE analysis was performed to confirm a high level of rhIDO1 purity.

### 3.9. hIDO1 Inhibitory Potency Evaluation

The activity of the compounds on hIDO1 was determined according to the previously reported fluorescence protocol [25]. Recombinant hIDO1 was prepared in-house as described above, and bovine catalase was purchased from Sigma-Aldrich (Sigma-Aldrich, St. Louis, MO, USA). Briefly, the reagents needed were dissolved in 50 mM potassium phosphate buffer (pH 6.5, 0.01%, *v*/*v* Tween 20), the reaction was performed in flat-bottomed, black 96-well microplates. After the addition of 10 μL 100 mM sodium ascorbate neutralized with an equimolar amount of 1 M NaOH, 10 μL 100 μM methylene blue, 10 μL 1 mg/mL bovine catalase, 2 μL of DMSO (negative control) or solution of a compound in DMSO, 50 μL 20 nM rhIDO1 and 18 μL 444 μML-Trp were added and the microplate was incubated for 30 min at 37 °C. The reaction was quenched with the addition of 100 μL 400 mM piperidine solution in MilliQ water and the formation of the examined fluorophore began following incubation at 65 °C for 20 min. The production of NFK-derived fluorophore (λ_EX_ = 400 nm, λ_EM_ = 500 nm) was measured using a microplate reader (Synergy H4; BioTek Instruments, Inc., Winooski, VT, USA) after 60 min post-incubation at room temperature. In the preliminary testing compounds were tested at 100 μM with activities determined as residual activities—RA, which were calculated to % of inhibition, presented in Table 1 and Table 2 (% of inhibition = 100% − RA). For compounds with RA values lower than 50% (higher inhibition than 50% at 100 μM), IC_50_ values were then calculated from determined RAs at 7 different concentrations with the help of GraphPad Prism 8 software (GraphPad Software, San Diego, CA, USA). All determinations were carried out in duplicates, with IC_50_ values defined with at least two independent determinations.

### 3.10. hIDO2 Inhibitory Potency Evaluation

Activity of the compounds on hIDO2 was determined according to the previously reported fluorescence protocol for hIDO1 [25] and reported absorbance protocols for hIDO2 [30,36] with several modifications. Recombinant hIDO2 was purchased from Vinci-Biochem (Vinci-Biochem, Srl, Vinci, Italy), bovine catalase was purchased from Sigma-Aldrich (Sigma-Aldrich, St. Louis, MO, USA). Prior to the measurement of activity for compounds, the enzyme activity was determined and optimal assay enzyme concentration was selected. The amounts of reagents needed to perform the assay were optimized. Briefly, the reagents needed were dissolved in 50 mM potassium phosphate buffer (pH 7.5, 0.01%, *v*/*v* Tween 20), the reaction was performed in black 384-well microplates. After the addition of 1 μL of 100 mM sodium ascorbate neutralized with an equimolar amount of 1M NaOH, 1 μL of 100 μM methylene blue, 1 μL of 1 mg/mL bovine catalase, 0.5 μL of DMSO (negative control) or solution of a compound in DMSO, 5 μL of 1.2 mM hIDO2 and 1.5 μL of 26.7 mM L-Trp were added and the microplate was incubated for 60 min at 37 °C. The reaction was quenched with the addition of 10 μL of 400 mM piperidine solution in MilliQ water and the formation of the examined fluorophore began following incubation at 65 °C for 20 min. The production of NFK-derived fluorophore (λ_EX_ = 400 nm, λ_EM_ = 500 nm) was measured using a microplate reader (Synergy H4; BioTek Instruments, Inc., Winooski, VT, USA) after 60 min post-incubation at room temperature. In the preliminary testing compounds were tested at 100 μM with activities determined as residual activities—RA, which were calculated to% of inhibition, presented in Table 1 and Table 2. (% of inhibition = 100% − RA). All determinations were carried out in duplicates.

### 3.11. hTDO Inhibitory Potency Evaluation

The activity of the compounds on hTDO was determined according to the previously reported fluorescence protocol for hIDO1 [25] and reported absorbance protocols for hTDO [30] with several modifications. Recombinant hTDO was purchased from Vinci-Biochem (Vinci-Biochem, Vinci, Italy), bovine catalase was purchased from Sigma-Aldrich (Sigma-Aldrich, St. Louis, MO, USA). Prior to the measurement of activity for compounds, the enzyme activity was determined, and optimal assay enzyme concentration was selected. The amounts of reagents needed to perform the assay were optimized. Briefly, the reagents needed were dissolved in 50 mM potassium phosphate buffer (pH 7.0, 0.01%, *v*/*v* Tween 20), the reaction was performed in black 384-well microplates. After the addition of 1 μL of 100 mM sodium ascorbate neutralized with equimolar amount of 1 M NaOH, 1 μL of 100 μM methylene blue, 1 μL of 1 mg/mL bovine catalase, 0.5 μL of DMSO (negative control) or solution of a compound, 5 μL of 300 nM hTDO and 1.5 μL of 3.3 mM L-Trp were added and the microplate was incubated for 60 min at 37 °C. The reaction was quenched with the addition of 10 μL of 400 mM piperidine solution in MilliQ water and the formation of the examined fluorophore began following incubation at 65 °C for 20 min. The production of NFK-derived fluorophore (λ_EX_ = 400 nm, λ_EM_ = 500 nm) was measured using a microplate reader (Synergy H4; BioTek Instruments, Inc., Winooski, VT, USA) after 60 min post-incubation at room temperature. In the preliminary testing compounds were tested at 100 μM with activities determined as residual activities—RA, which were calculated to% of inhibition, presented in Table 1 and Table 2 (% of inhibition = 100% − RA). All determinations were carried out in duplicates.

### 3.12. Cytotoxicity

Cytotoxicity of the compounds was determined on HepG2 cell line using the MTS cell proliferation assay (CellTiter 96 AQ_ueous_ One Solution Cell Proliferation Assay, Promega, Madison, WI, USA). The cells were seeded in 96-well plates (3 × 10^4^ cells/well) and maintained in Dulbecco’s Modified Eagle’s Medium, supplemented with 10% fetal bovine serum (both from Thermo Fisher Scientific, Waltham, MA, USA) and 100 µg/mL gentamicin (Sigma-Aldrich, St. Louis, MO, USA). Following overnight incubation in a humidified chamber at 37 °C and 5% CO_2_ cells were treated with the compounds of interest at final concentrations of 1 µM, 10 µM, 50 µM, 100 µM, 150 µM, and 200 µM. After 24h incubation under the same conditions, 100 µL of the medium from each well were collected and mixed with 10 µL of MTS solution. Following 3h incubation, the absorbance (λ_ABS_ = 490 nm) was measured using a microplate reader (Synergy H4; BioTek Instruments, Inc., Winooski, VT, USA). The results are expressed as means of triplicates ± standard deviation of three independent experiments.

### 3.13. Cell-Based IDO1 Inhibitory Assay

HeLa cells were seeded in 96-well plates (2 × 10^4^ cells/well) and maintained in Dulbecco’s modified Eagle’s medium, supplemented with 10% fetal bovine serum (both from Thermo Fisher Scientific, Waltham, MA, USA) and 100 µg/mL gentamicin (Sigma-Aldrich, St. Louis, MO, USA). Following overnight incubation in a humidified chamber at 37 °C and 5% CO_2_ cells were treated with human IFN-γ (50 ng/mL final concentration). After 24h incubation cells were treated with compounds (epacadostat at 3 μM to 0.3 nM was used as a positive control) in a total volume of 200 μL culture medium per well. Following overnight incubation, 140 μL of the supernatant from each well were transferred to a new 96-well plate, mixed with 10 μL of 6.1 N trichloroacetic acid and incubated at 50 °C for 30 min to hydrolize *N*-formylkynurenine, produced by uninhibited IDO1, to kynurenine. The reaction mixture was then centrifuged for 10 min at 2500 rpm to remove sediments. 100 μL of supernatant from each well were transferred to a new 96-well plate and mixed with 100 μL of 2%, *w*/*v p*-dimethylaminobenzaldehyde in acetid acid. The absorbance (λ_ABS_ = 480 nm) of the yellow color derivate formed after 10 min was measured using a microplate reader (Synergy H4; BioTek Instruments, Inc., Winooski, VT, USA).

### 3.14. Molecular Docking

Binding mode of compound **23** into the active site of IDO1 was predicted using the protein-ligand docking software GOLD 2020.0 (CCDC, Cambridge, UK). Prior to the docking, crystal structure 5WN8, retrieved from the Protein Data Bank, was visually examined in PyMOL 2.4.0 (Schrödinger, Munich, Germany) and subjected to crystal structure refinement (removal of water molecules and the addition of hydrogen atoms). The parameters used for docking are: the binding site was defined as all amino acid residues within 10 Å of the bound ligand, with residues being kept rigid during the docking process; coordination to the ferrous atom with default coordination geometry was included; the scoring function used for the docking was CHEMPLP, with search efficiency set to 200%. Docking poses of ligands were minimized using the MM2 force field implemented in Chem3D (PerkinElmer, Waltham, MA, USA).

## 4. Conclusions

In summary, a series of isoxazolo[5,4-*d*]pyrimidin-4(5*H*)-one-based selective IDO1 inhibitors was synthesized in the optimized 6- or 7-step synthetic procedure. Forty-two final compounds were obtained and key information about SARs of isoxazolo[5,4-*d*]pyrimidin-4(5*H*)-one scaffold were provided. The chemistry-driven optimization afforded modifications at two positions, on the left-hand (R^1^) and right-hand (R^2^) site of the central heterocyclic core. In general, the optimal R^1^ substituent was found to be 4-fluorophenyl, whereas most of the potent inhibitors contained *p*-substituted aniline as the R^2^ substituent, with **20**, **23**, and **32** as best-in-class inhibitors with IC_50_ values between 22 µM and 27 µM. The optimization of the fluorescence-based assay on hIDO2 and hTDO enabled the screening of all compounds against these two enzymes. None of the synthesized IDO1 inhibitors showed any significant activity in hIDO2 and hTDO assay. Therefore, we propose that our micromolar hIDO1-selective inhibitors with a novel chemical scaffold represent appealing chemical probes for further development of potent immunomodulators.

## Figures and Tables

**Table 1:** Inhibitory potencies of synthesized compounds **12**–**37** with 3-(4-fluorophenyl)isoxazolo[5,4-*d*]pyrimidin-4(5*H*)-one scaffold against IDO1, IDO2, and TDO.

3-(4-fluorophenyl)isoxazolo[5,4-d]pyrimidin-4(5H)-one scaffold				
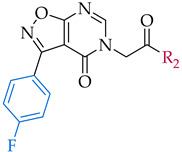				
Compound	R^2^	% of Inhibition @ 100 µM or IC_50_^a,b^		
		IDO1	IDO2	TDO
**12**	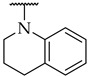	**IC_50_,** **50 ± 1 μM**	n.a. ^c^	n.a.
**13**	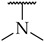	21 ± 5%	n.a.	n.a.
**14**	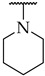	27 ± 4%	n.a.	n.a.
**15**	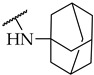	**IC_50_,** **88 ± 4 µM**	n.a.	n.a.
**16**	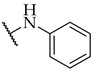	15 ± 4%	28 ± 9%	n.a.
**17**	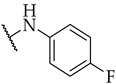	33 ± 2%	n.a.	n.a.
**18**	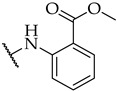	32 ± 4%	15 ± 1%	n.a.
**19**	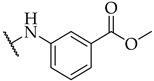	**IC_50_,** **55 ± 2 µM**	n.a.	n.a.
**20**	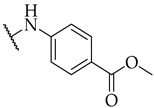	**IC_50_,** **27 ± 1 µM**	n.a.	n.a.
**21**	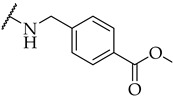	27 ± 3%	n.a.	n.a.
**22**	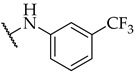	**IC_50_,** **55 ± 2 µM**	n.a.	n.a.
**23**	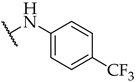	**IC_50_,** **23 ± 1 µM**	n.a.	n.a.
**24**	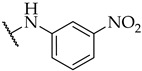	30 ± 2%	39 ± 5%	25 ± 8%
**25**	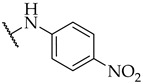	**IC_50_,** **98 ± 5 µM**	28 ± 0%	24 ± 7%
**26**	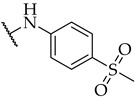	11 ± 2%	n.a.	n.a.
**27**	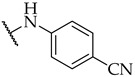	11 ± 4%	n.a.	17 ± 2%
**28**	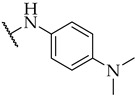	31 ± 3%	n.a.	18 ± 4%
**29**	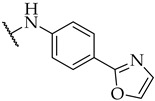	**IC_50_,** **30 ± 1 µM**	n.a.	29 ± 3%
**30**	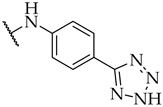	n.a.	n.a.	17 ± 2%
**31**	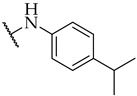	**IC_50_,** **87 ± 3 µM**	n.a.	n.a.
**32**	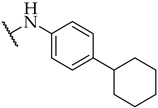	**IC_50_,** **22 ± 1 µM**	n.a.	n.a.
**33**	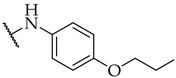	n.a.	n.a.	22 ± 4%
**34**	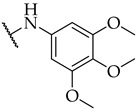	n.a.	n.a.	n.a.
**35**	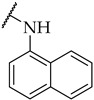	20 ± 6%	n.a.	n.a.
**36**	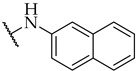	**IC_50_,** **46 ± 2 µM**	13 ± 2%	n.a.
**37**	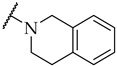	17 ± 5%	n.a.	n.a.

^a^ IC_50_ values are the means of at least two independent determinations, presented as mean ± SD; ^b^ Positive control: epacadostat (**1**), IC_50_ values obtained for IDO1, IDO2, and TDO were 53, 580, and 9620 nM, respectively; ^c^ n.a., not active.

**Table 2:** Inhibitory potencies of synthesized compounds **38**–**53** with 3-arylisoxazolo[5,4-*d*]pyrimidin-4(5*H*)-one scaffold against IDO1, IDO2, and TDO.

3-arylisoxazolo[5,4-d]pyrimidin-4(5H)-one scaffold					
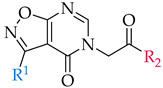					
Compound	R^1^	R^2^	% of Inhibition @ 100 µM or IC_50_^a,b^		
			IDO1	IDO2	TDO
**38**	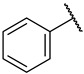	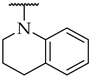	n.a. ^c^	16 ± 4%	n.a.
**39**	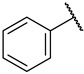	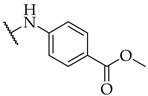	**IC_50_,** **22 ± 2 µM**	11 ± 7%	n.a.
**40**	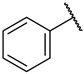	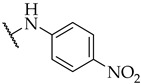	**IC_50_,** **127 ± 5 µM**	16 ± 5%	n.a.
**41**	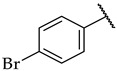	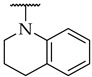	32 ± 1%	n.a.	n.a.
**42**	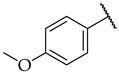	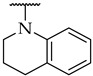	15 ± 4%	n.d. ^d^	n.d.
**43**	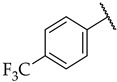	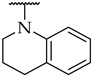	n.a.	n.d.	n.d.
**44**	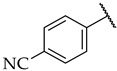	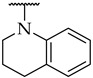	n.a.	n.a.	n.a.
**45**	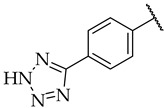	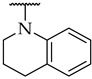	n.a.	n.a.	n.a.
**46**	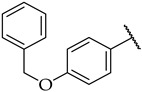	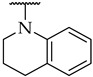	14 ± 3%	n.a.	n.a.
**47**	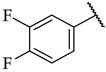	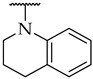	34 ± 1%	n.a.	n.a.
**48**	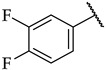	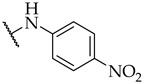	**IC_50_,** **75 ± 4 µM**	n.a.	10 ± 6%
**49**	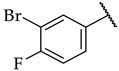	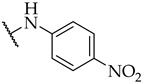	**IC_50_,** **96 ± 5 µM**	n.a.	10 ± 2%
**50**	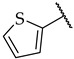	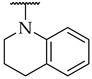	n.a.	n.a.	n.a.
**51**	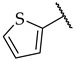	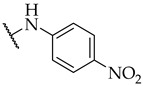	**IC_50_,** **53 ± 3 µM**	n.a.	n.a.
**52**	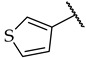	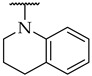	23 ± 5%	n.a.	n.a.
**53**	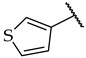	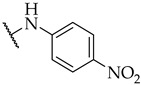	**IC_50_,** **40 ± 2 µM**	21 ± 6%	n.a.

^a^ IC_50_ values are the means of at least two independent determinations, presented as mean ± SD; ^b^ Positive control: epacadostat (**1**), IC_50_ values obtained for IDO1, IDO2, and TDO were 53, 580, and 9620 nM, respectively; ^c^ n.a., not active; ^d^ n.d., not determined.

## Data Availability

Data sharing not applicable.

## Supplementary Materials

The following are available online at https://www.mdpi.com/1424-8247/14/3/265/s1, Analytical data for synthesized compounds; Representative 1H NMR, 13C NMR and HPLC spectra for compounds **20**, **23**, **32**, **39,** and **53**; Determination of inhibitory potencies—Figure S1: Inhibitory potencies of epacadostat on hIDO1, hIDO2, and hTDO, Figure S2: Inhibitory potencies of most potent compounds **20**, **23**, **29**, **32,** and **39**.
