# Supplementary material for: Novel Selective IDO1 Inhibitors with Isoxazolo[5,4-d]pyrimidin-4(5H)-one Scaffold
Source: Pharmaceuticals (Basel). 2021 Mar 15;14(3):265. doi: 10.3390/ph14030265 (PMC8001472; doi:10.3390/ph14030265)
Supplement: Supplementary file 1 [file pharmaceuticals-14-00265-s001.pdf]

pharmaceuticals

Supplementary material on:

New selective IDO1 inhibitors with isoxazolo[5,4-*d*]pyrimidin-4(5*H*)-one scaffold

Ana Dolšak [1], Tomaž Bratkovič [1], Larisa Mlinarič [1], Eva Ogorevc [1], Urban Švajger [1,2], Stanislav Gobec [1], Matej Sova [1,*]

[1] University of Ljubljana, Faculty of Pharmacy, Aškerčeva 7, 1000 Ljubljana, Slovenia
[2] Blood Transfusion Centre of Slovenia, Šlajmerjeva 6, 1000 Ljubljana, Slovenia; urban.svajger@ztm.si
* Correspondence: matej.sova@ffa.uni-lj.si; Tel.: +386-1-4769577

Contents

1. Analytical data for synthesized compounds 2
  1.1 Aldehyde oximes (**6a-k**): 2
  1.2 5-aminoisoxazole-4-carboxamides (**8a-k**): 2
  1.3 Isoxazolo[5,4-d]pyrimidin-4(5H)-ones (**9a-k**): 3
  1.4 N-alkylants (**11a-y**): 4
  1.5 Final compounds (**12-53**): 7
2. Representative ^{1}H NMR, ^{13}C NMR and HPLC spectra 14
  2.1 Compound 20 14
  2.2 Compound 23 16
  2.3 Compound 32 18
  2.4 Compound 39 20
  2.5 Compound 53 22
3. Determination of inhibitory potencies 24
  3.1 Positive control 24
  3.2 Inhibitory potencies for the most potent compounds (graphs for IC_{50} calculations) 25
References 26

1. Analytical data for synthesized compounds

1.1 *Aldehyde oximes (**6a-k**):*

*4-fluorobenzaldehyde oxime (**6a**):* Yield: 88%; white solid; Mp 76 – 78 °C (lit. 85 – 87 °C [1]); ^{1}H NMR (400 MHz, DMSO-d_6): δ (ppm) = 7.21 – 7.27 (m, 2H), 7.61 – 7.67 (m, 2H), 8.15 (s, 1H), 11.24 (s, 1H); Rf = 0.48 (DCM/MeOH, 20:1, v/v).

*Benzaldehyde oxime (**6b**):* Yield: 98%; pale yellow oil; ^{1}H NMR (400 MHz, $CDCl_3$): δ (ppm) = 7.37 – 7.41 (m, 3H), 7.57 – 7.60 (m, 2H), 8.17 (s, 1H), 1H is exchanged as reported[1]; MS (ESI+) *m/z* calc. for C_7H_8NO $[M+H]^+$ 122.1, found 122.1; Rf = 0.55 (EtOAc/n-Hex, 1:2, v/v).

*4-bromobenzaldehyde oxime (**6c**):* Yield: 94%; white solid; Mp 95 – 97 °C (lit. 106 °C[1]); ^{1}H NMR (400 MHz, $CDCl_3$): δ (ppm) = 7.41 – 7.45 (m, 2H), 7.49 – 7.52 (m, 2H), 8.08 (s, 1H), 1H is exchanged; MS (ESI-) *m/z* calc. for C_7H_5BrNO $[M-H]^-$ 198.0, found 197.8; Rf = 0.64 (EtOAc/n-Hex, 2:1, v/v).

*4-methoxybenzaldehyde oxime (**6d**):* Yield: 95%; pale yellow oil; ^{1}H NMR (400 MHz, $CDCl_3$): δ (ppm) = 3.83 (s, 3H), 6.87 – 6.93 (m, 2H), 7.46 – 7.55 (m, 2H), 8.08 (s, 1H), 1H is exchanged as reported[1]; MS (ESI+) *m/z* calc. for $C_8H_{10}NO_2$ $[M+H]^+$ 152.1, found 152.0; Rf = 0.52 (EtOAc/n-Hex, 1:2, v/v).

*4-(trifluoromethyl)benzaldehyde oxime (**6e**):* Yield: 94%; white solid; Mp 88 – 90 °C; ^{1}H NMR (400 MHz, $CDCl_3$): δ (ppm) = 7.60 – 7.65 (m, 2H), 7.66 – 7.71 (m, 2H), 8.17 (s, 1H), 8.61 (s, 1H) as reported[1]; MS (ESI-) *m/z* calc. for $C_8H_5F_3NO$ $[M-H]^-$ 188.0, found 187.9; Rf = 0.56 (EtOAc/n-Hex, 1:2, v/v).

*4-((hydroxyimino)methyl)benzonitrile (**6f**):* Yield: 88%; white solid; Mp 104 – 106 °C; ^{1}H NMR (400 MHz, DMSO-d_6): δ (ppm) = 7.75 – 7.79 (m, 2H), 7.84 – 7.89 (m, 2H), 8.24 (s, 1H), 11.74 (s, 1H) as reported[1]; MS (ESI-) *m/z* calc. for $C_8H_5N_2O$ $[M-H]^-$ 145.0, found 144.9; Rf = 0.53 (EtOAc/n-Hex, 2:1, v/v).

*4-(benzyloxy)benzaldehyde oxime (**6g**):* Yield: 95%; white solid; Mp 92 – 94 °C (lit. 105 – 106 °C[2]; ^{1}H NMR (400 MHz, DMSO-d_6): δ (ppm) = 5.15 (s, 2H), 7.05 – 7.08 (m, 2H), 7.31 (s, 1H), 7.33 – 7.35 (m, 1H), 7.37 – 7.41 (m, 2H), 7.44 – 7.47 (m, 2H), 7.92 – 7.96 (m, 2H), 11.40 (br s, 1H); MS (ESI+) *m/z* calc. for $C_{14}H_{14}NO_2$ $[M+H]^+$ 228.1, found 227.9; MS (ESI+) *m/z* calc. for $C_{14}H_{13}NO_2Na$ $[M+Na]^+$ 250.1, found 249.9; Rf = 0.65 (EtOAc/n-Hex, 2:1, v/v).

*3,4-difluorobenzaldehyde oxime (**6h**):* Yield: 97%; white solid; Mp 61 – 64 °C; ^{1}H NMR (400 MHz, $CDCl_3$): δ (ppm) = 7.18 (dt, J_1 = 8.2 Hz, J_2 = 9.8 Hz, 1H), 7.25 – 7.29 (m, 1H), 7.46 (ddd, J_1 = 2.0 Hz, J_2 = 7.7 Hz, J_3 = 10.9 Hz, 1H), 8.06 (s, 1H), 1H is exchanged; MS (ESI-) m/z calc. for $C_7H_4F_2NO$ $[M-H]^-$ 156.0, found 155.8; Rf = 0.66 (EtOAc/n-Hex, 2:1, v/v).

*3-bromo-4-fluorobenzaldehyde oxime (**6i**):* Yield: 96%; white solid; Mp 78 – 79 °C; ^{1}H NMR (400 MHz, $CDCl_3$): δ (ppm) = 7.12 (t, J = 8.4 Hz, 1H), 7.48 (ddd, J_1= 2.1 Hz, J_2 = 4.7 Hz, J_3 = 8.5 Hz, 1H), 7.80 (dd, J_1 = 2.1 Hz, J_2 = 6.6 Hz, 1H), 8.05 (s, 1H), 1H is exchanged; MS (ESI-) *m/z* calc. for C_7H_4BrFNO $[M-H]^-$ 215.9, found 215.8; Rf = 0.28 (EtOAc/n-Hex, 2:1, v/v).

*Thiophene-2-carbaldehyde oxime (**6j**):* Yield: 71%; yellow solid; Mp 119 – 121 °C (lit. 124 – 128 °C[3]; ^{1}H NMR (400 MHz, $CDCl_3$): δ (ppm) = 7.11 (dd, J_1 = 3.7 Hz, J_2 = 5.1 Hz, 1H), 7.40 (dd, J_1 = 1.2 Hz, J_2 = 3.7 Hz, 1H), 7.58 (td, J_1 = 1.0 Hz, J_2 = 5.1 Hz, 1H), 7.73 (s, 1H), 8.38 (br s, 1H); MS (ESI+): *m/z* calc. for C_5H_6NOS $[M+H]^+$ 128.0, found 128.0; Rf = 0.37 (EtOAc/n-Hex, 1:2, v/v).

*Thiophene-3-carbaldehyde oxime (**6k**):* Yield: 82%; pale brown solid; Mp 100 – 102 °C (lit. 113 – 114 °C[4]); ^{1}H NMR (400 MHz, $CDCl_3$): δ (ppm) = 7.33 (dd, J_1 = 3.0 Hz, J_2 = 5.1 Hz, 1H), 7.46 (s, 1H), 7.50 (dd, J_1 = 1.2 Hz, J_2 = 5.1 Hz, 1H), 8.14 (dd, J_1 = 1.0 Hz, J_2 = 3.0 Hz, 1H), 1H from OH is exchanged; MS (ESI+) *m/z* calc. for C_5H_6NOS $[M+H]^+$ 128.0, found 127.9; Rf = 0.56 (EtOAc/n-Hex, 2:1, v/v).

1.2 *5-aminoisoxazole-4-carboxamides (**8a-k**):*

*5-amino-3-(4-fluorophenyl)isoxazole-4-carboxamide (**8a**):* Yield: 34%; pale orange solid; Mp 176 – 178 °C; ^{1}H NMR (400 MHz, DMSO-d_6): δ (ppm) = 7.32 – 7.38 (m, 2H), 7.58 – 7.63 (m, 2H), 7.65 (br s, 2H), 2H from NH_2 are exchanged as reported[5]; MS (ESI+) *m/z* calc. for $C_{10}H_8FN_3O_2Na^+$ $[M+Na]^+$ 244.05; found 244.18; Rf = 0.13 (EtOAc/n-Hex, 1:1, v/v).

*5-amino-3-phenylisoxazole-4-carboxamide (**8b**):* Yield: 22%; yellow solid; Mp 155 – 158 °C (lit. 182 – 185 °C[6]); ^{1}H NMR (400 MHz, DMSO-d_6): δ (ppm) = 5.50 (br s, 2H), 7.53 – 7.56 (m, 5H), 7.66 (s, 2H); MS (ESI-) *m/z* calc. for $C_{10}H_8N_3O_2$ $[M-H]^-$ 202.1, found 202.1; Rf = 0.42 (EtOAc/n-Hex, 4:1, v/v).

*5-amino-3-(4-bromophenyl)isoxazole-4-carboxamide (**8c**):* Yield: 13%; brown solid; Mp 99 – 101 °C; ^{1}H NMR (400 MHz, DMSO-*d*$_6$): δ (ppm) = 5.75 (s, 2H), 7.58 – 7.63 (m, 2H), 8.13 – 8.17 (m, 2H), 8.47 (s, 2H); MS (ESI-) *m/z* calc. for $C_{10}H_7BrN_3O_2$ [M-H]$^-$ 280.0, found 279.8; MS (ESI+) *m/z* calc. for $C_{10}H_8BrN_3O_2Na$ [M+Na]$^+$ 304.0, found 303.9; Rf = 0.28 (DCM/EtOAc, 7:10, v/v).

*5-amino-3-(4-methoxyphenyl)isoxazole-4-carboxamide (**8d**):* Yield: 71%; yellow oil; ^{1}H NMR (400 MHz, DMSO-*d*$_6$): δ (ppm) = 3.81 (s, 3H), 7.05 – 7.10 (m, 2H), 7.42 – 7.53 (m, 2H), 7.64 (s, 2H), 2H are exchanged; MS (ESI+) *m/z* calc. for $C_{11}H_{12}N_3O_3$ [M+H]$^+$ 234.1, found 234.0; Rf = 0.45 (EtOAc/n-Hex, 4:1, v/v).

*5-amino-3-(4-(trifluoromethyl)phenyl)isoxazole-4-carboxamide (**8e**):* Yield: 27%; orange oil; ^{1}H NMR (400 MHz, DMSO-*d*$_6$): δ (ppm) = 6.51 (br s, 2H), 7.70 (s, 2H), 7.76 – 7.80 (m, 2H), 7.84 – 7.88 (m, 2H); MS (ESI-) *m/z* calc. for $C_{11}H_7F_3N_3O_2$ [M-H]$^-$ 270.0, found 269.9; Rf = 0.53 (EtOAc/n-Hex, 4:1, v/v).

*5-amino-3-(4-cyanophenyl)isoxazole-4-carboxamide (**8f**):* Yield: 6%; pale brown solid; Mp 173 – 175 °C; ^{1}H NMR (400 MHz, DMSO-*d*$_6$): δ (ppm) = 6.54 (br s, 2H), 7.68 (s, 2H), 7.73 – 7.76 (m, 2H), 7.95 – 7.98 (m, 2H); MS (ESI-) *m/z* calc. for $C_{11}H_7N_4O_2$ [M-H]$^-$ 227.1, found 226.9; MS (ESI+) *m/z* calc. for $C_{11}H_8N_4O_2Na$ [M+Na]$^+$ 251.1, found 251.1; Rf = 0.28 (EtOAc/n-Hex, 2:1, v/v).

*5-amino-3-(4-(benzyloxy)phenyl)isoxazole-4-carboxamide (**8g**):* Yield: 15%; pale brown solid; Mp 136 – 139 °C; ^{1}H NMR (400 MHz, DMSO-*d*$_6$): δ (ppm) = 5.17 (s, 2H), 7.14 – 7.18 (m, 2H), 7.32 – 7.37 (m, 1H), 7.38 – 7.43 (m, 2H), 7.47 – 7.50 (m, 4H), 7.64 (s, 2H), 2H from NH_2 are exchanged; MS (ESI-): *m/z* calc. for $C_{17}H_{14}N_3O_3$ [M-H]$^-$ 308.1, found 308.0; MS (ESI+) *m/z* calc. for $C_{17}H_{15}N_3O_3Na$ [M+Na]$^+$ 332.1, found 332.2; MS (ESI+) *m/z* calc. for $C_{18}H_{19}N_3O_4Na$ [M+Na+MeOH]$^+$ 364.1, found 364.1; Rf = 0.33 (EtOAc/n-Hex, 2:1, v/v).

*5-amino-3-(3,4-difluorophenyl)isoxazole-4-carboxamide (**8h**):* Yield: 26%; pale brown solid; Mp 138 – 140 °C; ^{1}H NMR (400 MHz, DMSO-*d*$_6$): δ (ppm) = 7.38 – 7.43 (m, 1H), 7.57 (dt, J_1 = 8.4 Hz, J_2 = 10.7 Hz, 1H), 7.65 (ddd, J_1 = 2.1 Hz, J_2 = 3.5 Hz, J_3 = 11.4 Hz, 1H), 7.67 (s, 2H), 2H from NH_2 are exchanged; MS (ESI-): *m/z* calc. for $C_{10}H_6F_2N_3O_2$ [M-H]$^-$ 231.8, found 237.8; MS (ESI+) *m/z* calc. for $C_{10}H_7F_2N_3O_2Na$ [M+Na]$^+$ 262.0, found 261.9; MS (ESI+) *m/z* calc. for $C_{11}H_{11}F_2N_3O_3Na$ [M+Na+MeOH]$^+$ 294.1, found 293.9; Rf = 0.38 (EtOAc/n-Hex, 2:1, v/v).

*5-amino-3-(3-bromo-4-fluorophenyl)isoxazole-4-carboxamide (**8i**):* Yield: 15%; brown solid; Mp 132 – 134 °C; ^{1}H NMR (400 MHz, DMSO-*d*$_6$): δ (ppm) = 6.45 (br s, 2H), 7.50 (t, *J* = 8.7 Hz, 1H), 7.58 – 7.63 (m, 1H), 7.66 (s, 2H), 7.87 (dd, J_1 = 2.0 Hz, J_2 = 6.8 Hz, 1H); MS (ESI-): *m/z* calc. for $C_{10}H_6BrFN_3O_2$ [M-H]$^-$ 298.0, found 297.8; MS (ESI+): *m/z* calc. for $C_{10}H_8BrFN_3O_2$ [M+H]$^+$ 300.0, found 299.9; MS (ESI+) *m/z* calc. for $C_{10}H_7BrFN_3O_2Na$ [M+Na]$^+$ 322.0, found 321.9; MS (ESI+) *m/z* calc. for $C_{11}H_{11}BrFN_3O_2Na$ [M+Na+MeOH]$^+$ 354.0, found 353.8; Rf = 0.31 (DCM/EtOAc, 7:10, v/v).

*5-amino-3-(thiophen-2-yl)isoxazole-4-carboxamide (**8j**):* Yield: 24%; yellow solid; Mp 135 – 138 °C; ^{1}H NMR (400 MHz, DMSO-*d*$_6$): δ (ppm) = 6.68 (br s, 2H), 7.20 – 7.23 (m, 1H), 7.58 – 7.66 (m, 3H), 7.73 – 7.76 (m, 1H); MS (ESI-) *m/z* calc. for $C_8H_6N_3O_2S$ [M-H]$^-$ 208.0, found 208.0; Rf = 0.38 (EtOAc/n-Hex, 4:1, v/v).

*5-amino-3-(thiophen-3-yl)isoxazole-4-carboxamide (**8k**):* Yield: 38%; brown solid; Mp 165 – 169 °C; ^{1}H NMR (400 MHz, DMSO-*d*$_6$): δ (ppm) = 7.34 (dd, J_1 = 1.3 Hz, J_2 = 5.0 Hz, 1H), 7.74 (dd, J_1 = 2.9 Hz, J_2 = 5.0 Hz, 1H), 7.97 (dd, J_1= 1.3 Hz, J_2= 2.9 Hz, 1H), 4H from NH_2 and $CONH_2$ are exchanged; MS (ESI+) *m/z* calc. for $C_8H_7N_3NaO_2S$ [M+Na]$^+$ 232.0, found 231.9; *m/z* calc. for $C_9H_{11}N_3NaO_3S$ [M+Na+MeOH]$^+$ 264.0, found 263.9; Rf = 0.22 (EtOAc/n-Hex, 2:1, v/v).

1.3 *Isoxazolo[5,4-d]pyrimidin-4(5H)-ones (**9a-k**):*

*3-(4-fluorophenyl)isoxazolo[5,4-d]pyrimidin-4(5H)-one (**9a**):* Yield: 57%; pale yellow solid; Mp 233 - 237 °C; ^{1}H NMR (400 MHz, DMSO-*d*$_6$): δ (ppm) = 7.40 – 7.46 (m, 2H), 8.35 – 8.40 (m, 2H), 8.46 (s, 1H), 13.19 (s, 1H) as reported[5]; MS (ESI-) *m/z* calc. for $C_{11}H_5FN_3O_2^-$ [M-H]$^-$ 230.04; found 230.20; Rf = 0.18 (EtOAc/n-Hex, 1:1, v/v).

*3-phenylisoxazolo[5,4-d]pyrimidin-4(5H)-one (**9b**):* Yield: 68%; pale yellow solid; Mp 216 – 218 °C; ^{1}H NMR (400 MHz, DMSO-*d*$_6$): δ (ppm) = 7.55 – 7.59 (m, 3H), 8.26 – 8.30 (m, 2H), 8.45 (s, 1H), 13.14 (s, 1H) as reported[7]; MS (ESI-) *m/z* calc. for $C_{11}H_6N_3O_2$ [M-H]$^-$ 212.0, found 211.9; Rf = 0.46 (EtOAc/n-Hex, 4:1, v/v).

3-(4-bromophenyl)isoxazolo[5,4-d]pyrimidin-4(5H)-one (***9c***)*:* Yield: 8%; pale brown solid; Mp 175 – 177 °C; ^{1}H NMR (400 MHz, DMSO-*d$_6$*): δ (ppm) = 7.78 – 7.81 (m, 2H), 8.24 – 8.28 (m, 2H), 8.47 (s, 1H), 13.20 (s, 1H); MS (ESI-): *m/z* calc. for $C_{11}H_5BrN_3O_2$ [M-H]$^-$ 290.0, found 289.8; Rf = 0.31 (EtOAc/n-Hex, 2:1, v/v).

3-(4-methoxyphenyl)isoxazolo[5,4-d]pyrimidin-4(5H)-one (***9d***)*:* Yield: 36%; pale yellow solid; Mp 174 – 176 °C; ^{1}H NMR (400 MHz, DMSO-*d$_6$*): δ (ppm) = 3.84 (s, 3H), 7.08 – 7.12 (m, 2H), 8.32 – 8.36 (m, 3H), 12.69 (br s, 1H) as reported[7]; MS (ESI-) *m/z* calc. for $C_{12}H_8N_3O_3$ [M-H]$^-$ 242.1, found 241.9; MS (ESI+) *m/z* calc. for $C_{12}H_{10}N_3O_3$ [M+H]$^+$ 244.1, found 244.0; Rf = 0.46 (EtOAc/n-Hex, 4:1, v/v).

3-(4-(trifluoromethyl)phenyl)isoxazolo[5,4-d]pyrimidin-4(5H)-one (***9e***)*:* Yield: 13%; orange solid; Mp 146 – 149 °C; ^{1}H NMR (400 MHz, DMSO-*d$_6$*): δ (ppm) = 7.91 – 7.96 (m, 2H), 8.36 (s, 1H), 8.57 – 8.62 (m, 2H), 1H is exchanged; MS (ESI-) *m/z* calc. for $C_{12}H_5F_3N_3O_2$ [M-H]$^-$ 280.0, found 279.9; Rf = 0.53 (EtOAc/n-Hex, 4:1, v/v).

4-(4-oxo-4,5-dihydroisoxazolo[5,4-d]pyrimidin-3-yl)benzonitrile (***9f***)*:* Yield: 21%; white solid; Mp 200 – 201 °C; ^{1}H NMR (400 MHz, DMSO-*d$_6$*): δ (ppm) = 8.05 – 8.08 (m, 2H), 8.47 – 8.50 (m, 2H), 8.49 (s, 1H), 13.26 (s, 1H); MS (ESI-): *m/z* calc. for $C_{12}H_5N_4O_2$ [M-H]$^-$ 237.0, found 236.9; Rf = 0.33 (EtOAc/n-Hex, 2:1, v/v).

3-(4-(benzyloxy)phenyl)isoxazolo[5,4-d]pyrimidin-4(5H)-one (***9g***)*:* Yield: 23%; white solid; Mp 139 – 140 °C; ^{1}H NMR (400 MHz, DMSO-*d$_6$*): δ (ppm) = 5.21 (s, 2H), 7.18 – 7.21 (m, 2H), 7.34 – 7.37 (m, 1H), 7.39 – 7.43 (m, 2H), 7.47 – 7.50 (m, 2H), 8.26 – 8.30 (m, 2H), 8.44 (s, 1H), 13.12 (s, 1H); MS (ESI-): *m/z* calc. for $C_{18}H_{12}N_3O_3$ [M-H]$^-$ 318.1, found 318.0; Rf = 0.37 (EtOAc/n-Hex, 2:1, v/v).

3-(3,4-difluorophenyl)isoxazolo[5,4-d]pyrimidin-4(5H)-one (***9h***)*:* Yield: 10%; yellow solid; Mp 146 – 149 °C; ^{1}H NMR (400 MHz, DMSO-*d$_6$*): δ (ppm) = 7.68 (dt, J_1 = 8.6 Hz, J_2 = 10.6 Hz, 1H), 8.18 – 8.23 (m, 1H), 8.46 (ddd, J_1 = 2.1 Hz, J_2 = 7.8 Hz, J_3 = 10.6 Hz, 1H), 8.49 (s, 1H), 13.25 (s, 1H); MS (ESI-): *m/z* calc. for $C_{11}H_4F_2N_3O_2$ [M-H]$^-$ 248.0, found 247.8; Rf = 0.53 (EtOAc/n-Hex, 2:1, v/v).

3-(3-bromo-4-fluorophenyl)isoxazolo[5,4-d]pyrimidin-4(5H)-one (***9i***)*:* Yield: 32%; white solid; Mp 166 – 169 °C; ^{1}H NMR (400 MHz, DMSO-*d$_6$*): δ (ppm) = 7.61 (t, *J* = 8.7 Hz, 1H), 8.34 (ddd, J_1 = 2.2 Hz, J_2 = 4.8 Hz, J_3 = 8.7 Hz, 1H), 8.48 (s, 1H), 8.74 (dd, J_1 = 2.2 Hz, J_2 = 6.8 Hz, 1H), 13.16 (s, 1H); MS (ESI-): *m/z* calc. for $C_{11}H_4FBrN_3O_2$ [M-H]$^-$ 307.9, found 307.8; Rf = 0.22 (EtOAc/n-Hex, 2:1, v/v).

3-(thiophen-2-yl)isoxazolo[5,4-d]pyrimidin-4(5H)-one (***9j***)*:* Yield: 85%; pale yellow solid; Mp 212 – 214 °C; ^{1}H NMR (400 MHz, DMSO-*d$_6$*): δ (ppm) = 7.29 (dd, J_1 = 3.7 Hz, J_2 = 5.1 Hz, 1H), 7.85 (dd, J_1 = 1.2 Hz, J_2 = 5.1 Hz, 1H), 8.46 (s, 1H), 8.59 (dd, J_1 = 1.2 Hz, J_2 = 3.7 Hz, 1H), 13.20 (br s, 1H); MS (ESI-): *m/z* calc. for $C_9H_4N_3O_2S$ [M-H]$^-$ 218.0, found 217.9; Rf = 0.50 (EtOAc/n-Hex, 4:1, v/v).

3-(thiophen-3-yl)isoxazolo[5,4-d]pyrimidin-4(5H)-one (***9k***)*:* Yield: 59%; pale brown solid; Mp 202 – 204 °C; ^{1}H NMR (400 MHz, DMSO-*d$_6$*): δ (ppm) = 7.77 – 7.79 (m, 2H), 8.45 (s, 1H), 8.97 (dd, J_1 = 1.4 Hz, J_2 = 2.8 Hz, 1H), 13.18 (s, 1H); MS (ESI-): *m/z* calc. for $C_9H_5N_3O_2S$ [M-H]$^-$ 218.0, found 217.8; Rf = 0.44 (EtOAc/n-Hex, 2:1, v/v).

*1.4 N-alkylants (**11a-y**):*

2-chloro-N,N-dimethylacetamide (***11a***)*:* Yield: quantitative; brown oil; ^{1}H NMR (400 MHz, $CDCl_3$): δ (ppm) = 2.98 (s, 3H), 3.09 (s, 3H), 4.07 (s, 2H); MS (ESI+) *m/z* calc. for $C_4H_9ClNO^+$ [M+H]$^+$ 122.0, found 122.1.

2-chloro-1-(piperidin-1-yl)ethan-1-one (***11b***)*:* Yield: 48%; brown oil; ^{1}H NMR (400 MHz, $CDCl_3$): δ (ppm) = 1.52 – 1.59 (m, 2H), 1.59 – 1.69 (m, 4H), 3.40 – 3.45 (m, 2H), 3.52 – 3.57 (m, 2H), 4.05 (s, 2H) as reported[8]; Rf = 0.45 (EtOAc/n-Hex; 2:1; v/v).

N-(adamantan-2-yl)-2-chloroacetamide (***11c***)*:* Yield: 68%; pale brown crystals; Mp 95 – 97 °C; ^{1}H NMR (400 MHz, $CDCl_3$): δ (ppm) = 1.68 – 1.70 (m, 6H), 2.00 – 2.03 (m, 6H), 2.08 – 2.12 (m, 3H), 3.93 (s, 2H), 6.22 (s, 1H) as reported[9]; MS (ESI+) *m/z* calc. for $C_{12}H_{19}ClNO^+$ [M+H]$^+$ 228.1, found 227.9; *m/z* calc. for $C_{12}H_{17}ClNO^-$ [M+H]$^+$ 226.1, found 225.8.

2-chloro-N-phenylacetamide (***11d***)*:* Yield: quantitative; pale yellow solid; Mp 120 – 122 °C; ^{1}H NMR (400 MHz, DMSO-d$_6$): δ (ppm) = 4.26 (s, 2H), 7.06 – 7.10 (m, 1H), 7.30 – 7.35 (m, 2H), 7.57 – 7.61 (m, 2H), 10.41 (s, 1H) as reported[10]; MS (ESI+): *m/z* calc. for C_8H_9ClNO [M+H]$^+$ 170.0, found none; Rf = 0.38 (EtOAc/n-Hex, 1:2, v/v).

2-chloro-N-(4-fluorophenyl)acetamide (***11e****)*: Yield: 63%; silver solid, Mp 110 – 112 °C; ^{1}H NMR (400 MHz, $CDCl_3$): δ (ppm) = 4.20 (s, 2H), 7.03 – 7.09 (m, 2H), 7.49 – 7.54 (m, 2H), 8.21 (s, 1H) as reported[10]; MS (ESI+/-): *m/z* calc for C_8H_6ClFNO [M-H]$^-$ 186.0, found 185.8; Rf = 0.50 (EtOAc/n-Hex, 1:1, v/v).

Methyl 2-(2-bromoacetamido)benzoate (***11f****)*: Yield: 64%; white solid; Mp 76 – 78 °C; ^{1}H NMR (400 MHz, DMSO-d_6): δ (ppm) = 3. 87 (s, 3H), 4.23 (s, 2H), 7.25 (ddd, J_1 = 1.2, J_2 = 7.4, J_3 = 7.9 Hz, 1H), 7.62 – 7.67 (m, 1H), 7.93 – 7.96 (m, 1H), 8.23 (dd, J_1 = 1.1, J_2 = 8.4 Hz, 1H), 11.07 (s, 1H) as reported[11]; MS (ESI+) *m/z* calc. For $C_{10}H_{11}BrNO_3^+$ [M+H]$^+$ 271.99, found 272.1; Rf = 0.66 (EtOAc/n-Hex; 1:1; v/v).

Methyl 3-(2-bromoacetamido)benzoate (***11g****)*: Prior to the acylation, esterification of starting material 3-aminobenzoic acid (10.0 g, 72.9 mmol, 1.0 equiv.) was performed in anhydrous MeOH (60 mL) at 0 °C, to which $SOCl_2$ (10.6 mL, 145.8 mmol, 2.0 equiv.) was added drop-wise. Reaction mixture was stirred 15 min at 0 °C, 15 min at room temperature and refluxed for 3 hours. After completion, the solvents were removed under reduced pressure. The addition of Et_2O to the almost dry residue afforded the precipitation of a product m*ethyl 3-aminobenzoate*, which was collected by filtration. Yield: quantitative; white solid; Mp 106 – 108 °C; ^{1}H NMR (400 MHz, DMSO-*d6*): δ (ppm) = 3.87 (s, 3H), 7.54 – 7.61 (m, 2H), 7.84 – 7.87 (m, 2H); MS (ESI-) *m/z* calc. for $C_8H_8NO_2$ [M-H]$^-$ 150.1, found 149.8; Rf = 0.35 (EtOAc/n-Hex; 1:2; v/v). *Methyl 3-aminobenzoate* (3.0 g, 20.0 mmol, 1.0 equiv.) was subjected to procedure V to yield m*ethyl 3-(2-bromoacetamido)benzoate* (***11g****)*. Yield: 61%; white solid; Mp 83 – 85 °C; ^{1}H NMR (400 MHz, $CDCl_3$): δ (ppm) = 3.93 (s, 3H), 4.05 (s, 2H), 7.45 (t, J_1 = 8.0 Hz, 1H), 7.83 – 7.86 (m, 1H), 7.91 (ddd, J_1 = 1.0 Hz, J_2 = 2.2 Hz, J_3 = 8.1 Hz, 1H), 8.05 (t, J = 1.9 Hz, 1H), 8.26 (br s, 1H); MS (ESI+) *m/z* calc. for $C_{10}H_{11}BrNO_3^+$ [M+H]$^+$ 272.0, found 272.1 as reported[12]; *m/z* calc. for $C_{10}H_{10}BrNNaO_3^+$ [M+Na]$^+$ 293.97, found 294.0. Rf = 0,50 (EtOAc/n-Hex; 1:1; v/v).

Methyl 4-(2-bromoacetamido)benzoate (***11h****)*: Prior to the acylation, esterification of starting material 4-aminobenzoic acid (10.0 g, 72.9 mmol, 1.0 equiv.) was done according to the described procedure above. *Methyl 4-aminobenzoate:* Yield: quantitative; white solid; Mp 143 – 145 °C; ^{1}H NMR (400 MHz, DMSO-*d6*): δ (ppm) = 3.75 (s, 3H), 6.12 (br s, 2H), 6.72 – 6.77 (m, 2H), 7.69 – 7.73 (m, 2H); MS (ESI-) *m/z* calc. for $C_8H_8NO_2$ [M-H]$^-$ 150.1, found 149.7; MS (ESI+) *m/z* calc. for $C_8H_{10}NO_2$ [M+H]$^+$ 152.1, found 152.2; Rf = 0.30 (EtOAc/n-Hex; 1:2; v/v). *Methyl 4-aminobenzoate* (3.0 g, 20.0 mmol, 1.0 equiv.) was subjected to procedure V to yield *methyl 4-(2-bromoacetamido)benzoate* (***11h****)*: Yield: 71%; white solid; Mp 122 – 124 °C; ^{1}H NMR (400 MHz, $CDCl_3$): δ (ppm) = 3.91 (s, 3H), 4.04 (s, 2H), 7.62 – 7.67 (m, 2H), 8.03 – 8.07 (m, 2H), 8.26 (br s, 1H) as reported[13]; MS (ESI+) *m/z* calc. for $C_{10}H_{11}BrNO_3^+$ [M+H]$^+$ 272.0, found 272.1; Rf = 0,47 (EtOAc/n-Hex; 1:1; v/v).

Methyl 4-((2-chloroacetamido)methyl)benzoate (***11i****)*: Yield: 24%; brown oil; ^{1}H NMR (400 MHz, $CDCl_3$): δ (ppm) = 3.92 (s, 3H), 4.14 (s, 2H), 4.56 (d, J = 6.0 Hz, 2H), 6.94 (br s, 1H), 7.34 – 7.37 (m, 2H), 8.01 – 8.04 (m, 2H) as reported[14]; MS (ESI+) *m/z* calc. for $C_{11}H_{12}ClNNaO_3$ [M+Na]$^+$ 264.0; found 263.8; *m/z* calc. for $C_{12}H_{16}ClNNaO_4$ [M+Na+MeOH]$^+$ 296.1; found 295.9; Rf = 0.42 (EtOAc/n-Hex; 2:1, v/v).

2-chloro-N-(3-(trifluoromethyl)phenyl)acetamide (***11j****)*: Yield: 82%; white solid; Mp 65 – 68 °C; ^{1}H NMR (400 MHz, $CDCl_3$): δ (ppm) = 4.22 (s, 2H), 7.42 – 7.46 (m, 1H), 7.47 – 7.52 (m, 1H), 7.75 – 7.80 (M, 1H), 7.84 – 7.87 (m, 1H), 8.33 (br s, 1H) as reported[15]; MS (ESI-) *m/z* calc. for $C_9H_6ClF_3NO$ [M-H]$^-$ 236.0; found 235.7; Rf = 0.67 (DCM/EtOAc; 10:7, v/v).

2-chloro-N-(4-(trifluoromethyl)phenyl)acetamide (***11k****)*: Yield: 90%; white crystals; Mp 125 – 128 °C; ^{1}H NMR (400 MHz, $CDCl_3$): δ (ppm) = 4.22 (s, 2H), 7.61 – 7.64 (m, 2H), 7.69 – 7.71 (m, 2H), 8.36 (s, 1H) as reported[10]; MS (ESI-): *m/z* calc. for $C_9H_6ClF_3NO$ [M-H]$^-$ 236.0, found 235.8; Rf = 0.81 (EtOAc/n-Hex, 2:1, v/v).

2-chloro-N-(3-nitrophenyl)acetamide (***11l****)*: Yield: 82%; light brown crystals; Mp 89 – 90 °C; ^{1}H NMR (400 MHz, DMSO-*d6*): δ (ppm) = 4.32 (s, 2H), 7.64 (t, J = 8.2 Hz, 1H), 7.92 (ddd, J_1 = 0.8 Hz, J_2 = 2.0 Hz, J_3 = 8.1 Hz, 1H), 7.96 (ddd, J_1 = 0.9 Hz, J_2 = 2.3 Hz, J_3 = 8.2 Hz, 1H), 8.61 (t, J = 2.2 Hz, 1H), 10.84 (s, 1H) as reported[16]; MS (ESI-) *m/z* calc. for $C_8H_6ClN_2O_3$ [M-H]$^-$ 213.0, found 212.9; Rf = 0.64 (DCM/EtOAc, 10:7, v/v).

2-chloro-N-(4-nitrophenyl)acetamide (***11m****)*: Yield: 74%; brown solid; Mp 130 – 133 °C; ^{1}H NMR (400 MHz, DMSO-*d6*): δ (ppm) = 4.34 (s, 2H), 7.82 – 7.86 (m, 2H), 8.22 – 8.26 (m, 2H), 10.90 (s, 1H); MS (ESI-): *m/z* calc. for $C_8H_6ClN_2O_3$ [M-H]$^-$ 213.0, found 212.8; Rf = 0.30 (EtOAc/n-Hex, 1:1, v/v).

2-chloro-N-(4-(methylsulfonyl)phenyl)acetamide (***11n***)*:* Yield: 89%; light brown solid; Mp 136 – 137 °C; ^{1}H NMR (400 MHz, $CDCl_3$): δ (ppm) = 3.06 (s, 3H), 4.24 (s, 2H), 7.77 – 7.81 (m, 2H), 7.93 – 7.97 (m, 2H), 8.45 (s, 1H); MS (ESI-) *m/z* calc. for $C_9H_9ClNO_3S$ [M-H]$^-$ 246.0, found 246.0; MS (ESI+) *m/z* calc. for $C_9H_{10}ClNO_3SNa$ [M+Na]$^+$ 270.0, found 270.0; Rf = 0.25 (EtOAc/n-Hex, 2:1, v/v).

2-chloro-N-(4-cyanophenyl)acetamide (***11o***)*:* Yield: 73%; yellow solid; Mp 160 – 163 °C (lit. 184 – 187 °C[17]); ^{1}H NMR (400 MHz, $CDCl_3$): δ (ppm) = 4.22 (s, 2H), 7.65 – 7.68 (m, 2H), 7.70 – 7.73 (m, 2H), 8.39 (s, 1H); MS (ESI-) *m/z* calc. for $C_9H_6ClN_2O$ [M-H]$^-$ 193.0, found 193.0; Rf = 0.20 (EtOAc/n-Hex, 1:2, v/v).

2-chloro-N-(4-(dimethylamino)phenyl)acetamide (***11p***)*:* Yield: 65%; black crystals; Mp 124 – 126 °C; ^{1}H NMR (400 MHz, $CDCl_3$): δ (ppm) = 2.93 (s, 6H), 4.17 (s, 2H), 6.69 – 6.73 (m, 2H), 7.35 – 7.39 (m, 2H), 8.09 (s, 1H); MS (ESI+): *m/z* calc. for $C_{10}H_{13}ClN_2NaO$ [M+Na]$^+$ 235.1, found 234.9; *m/z* calc. for $C_{10}H_{13}ClKN_2O$ [M+K]$^+$ 251.0, found 251.0; *m/z* calc. for $C_{11}H_{17}ClN_2NaO_2$ [M+Na+MeOH]$^+$ 267.1, found 266.9; Rf = 0.44 (EtOAc/n-Hex, 2:1, v/v).

2-chloro-N-(4-(oxazol-2-yl)phenyl)acetamide (***11q***)*:* Yield: 88%; yellow solid; Mp 150 – 153 °C; ^{1}H NMR (400 MHz, DMSO-d_6): δ (ppm) = 4.28 (s, 2H), 7.60 – 7.61 (m, 1H), 7.70 – 7.71 (m, 4H), 8.41 – 8.42 (m, 1H), 10.52 (s, 1H); MS (ESI-) *m/z* calc. for $C_{11}H_8ClN_2O_2$ [M-H]$^-$ 235.0, found 235.0; MS (ESI+) *m/z* calc. for $C_{11}H_{10}ClN_2O_2$ [M+H]$^+$ 237.0, found 237.1; Rf = 0.30 (EtOAc/n-Hex, 1:1, v/v).

2-chloro-N-(4-isopropylphenyl)acetamide (***11r***)*:* Yield: 93%; brown solid, Mp 120 – 123 °C (lit. 141 – 143 °C[18]); ^{1}H NMR (400 MHz, $CDCl_3$): δ (ppm) = 1.18 (d, *J* = 6.9 Hz, 6H), 2.84 (hept, *J* = 6.9 Hz, 1H), 4.22 (s, 2H), 7.18 – 7.21 (m, 2H), 7.47 – 7.50 (m, 2H), 10.20 (s, 1H); MS (ESI-) *m/z* calc. for $C_{11}H_{13}ClNO$ [M-H]$^-$ 210.1, found 210.0; MS (ESI+) *m/z* calc. for $C_{11}H_{15}ClNO$ [M+H]$^+$ 212.1, found 212.0; *m/z* calc. for $C_{11}H_{14}ClNO$ [M+Na]$^+$ 234.1, found 234.1; *m/z* calc. for $C_{12}H_{18}ClNO_2Na$ [M+Na]$^+$ 266.1, found 266.1; Rf = 0.70 (EtOAc/n-Hex, 2:1, v/v).

2-chloro-N-(4-cyclohexylphenyl)acetamide (***11s***)*:* Yield: 90%; pale orange crystals; Mp 114 – 118 °C; ^{1}H NMR (400 MHz, $CDCl_3$): δ (ppm) = 1.19 – 1.29 (m, 1H), 1.36 – 1.41 (m, 4H), 1.71 – 1.76 (m, 1H), 1.80 – 1.87 (m, 4H), 2.45 – 2.52 (m, 1H), 4.19 (s, 2H), 7.18 – 7.21 (m, 2H), 7.42 – 7.46 (m, 2H), 8.19 (s, 1H); MS (ESI+/-): *m/z* calc. for $C_{14}H_{18}ClNNaO$ [M+Na]$^+$ 274.1, found 274.0; *m/z* calc. for $C_{15}H_{22}ClNNaO_2$ [M+Na+MeOH]$^+$ 306.1, found 306.0; *m/z* calc. for $C_{14}H_{17}ClNO$ [M-H]$^-$ 250.1, found 249.9. Rf = 0.79 (EtOAc/n-Hex, 2:1, v/v).

N-(4-butoxyphenyl)-2-chloroacetamide (***11t***)*:* Yield: 97%; brown crystals; Mp 111 – 113 °C; ^{1}H NMR (400 MHz, $CDCl_3$): δ (ppm) = 0.97 (t, *J* = 7.4 Hz, 3H), 1.44 – 1.53 (m, 2H), 1.72 – 1.79 (m, 2H), 3.95 (t, *J* = 6.5 Hz, 2H), 4.19 (s, 2H), 6.86 – 6.90 (m, 2H), 7.40 – 7.44 (m, 2H), 8.13 (s, 1H). MS (ESI+/-): *m/z* calc. for $C_{12}H_{16}ClNNaO_2$ [M+Na]$^+$ 264.1, found 264.0; *m/z* calc. for $C_{13}H_{20}ClNNaO_3$ [M+Na+MeOH]$^+$ 296.1, found 296.0; *m/z* calc. for $C_{12}H_{15}ClNO_2$ [M-H]$^-$ 240.1, found 239.8. Rf = 0.64 (EtOAc/n-Hex, 2:1, v/v).

2-chloro-N-(3,4,5-trimethoxyphenyl)acetamide (***11u***)*:* Yield: 81%; white crystals; Mp 95 – 99 °C; ^{1}H NMR (400 MHz, $CDCl_3$): δ (ppm) = 3.82 (s, 3H), 3.85 (s, 6H), 4.18 (s, 2H), 6.83 (s, 2H), 8.17 (s, 1H) as reported[19]. MS (ESI+/-): *m/z* calc. for $C_{11}H_{14}ClNNaO_4$ [M+Na]$^+$ 282.1, found 281.9; *m/z* calc. for $C_{12}H_{18}ClNNaO_5$ [M+Na+MeOH]$^+$ 314.1, found 314.0; *m/z* calc. for $C_{11}H_{13}ClNO_4$ [M-H]$^-$ 258.1, found 257.8. Rf = 0.40 (EtOAc/n-Hex, 2:1, v/v).

2-chloro-N-(naphthalen-1-yl)acetamide (***11v***)*:* Yield: 82%; violet solid; Mp 143 – 145 °C; ^{1}H NMR (400 MHz, $CDCl_3$): δ (ppm) = 4.36 (s, 2H), 7.51 (t, *J* = 8.0 Hz, 1H), 7.52-7.60 (m, 2H), 7.74 – 7.78 (m, 1H), 7.86 – 7.92 (m, 2H), 7.98 – 8.02 (m, 1H), 8.78 (br s, 1H) as reported [10]; MS (ESI-) *m/z* calc. for $C_{12}H_9ClNO$ [M-H]$^-$ 218.0, found 218.1; MS (ESI+) *m/z* calc. for $C_{12}H_{11}ClNO$ [M+H]$^+$ 220.1, found 220.0; *m/z* calc. for $C_{12}H_{10}ClNONa$ [M+Na]$^+$ 242.0, found 242.0; Rf = 0.23 (EtOAc/n-Hex, 1:2, v/v).

2-chloro-N-(naphthalen-2-yl)acetamide (***11w***)*:* Yield: 95%; white solid; Mp 102 – 104 °C; ^{1}H NMR (400 MHz, $CDCl_3$): δ (ppm) = 4.26 (s, 2H), 7.42 – 7.53 (m, 3H), 7.79 – 7.86 (m, 3H), 8.22 (d, *J* = 1.8 Hz, 1H), 8.39 (s, 1H) as reported[10]; MS (ESI-) *m/z* calc. for $C_{12}H_9ClNO$ [M-H]$^-$ 218.0, found 218.1; MS (ESI+) *m/z* calc. for $C_{12}H_{10}ClNONa$ [M+Na]$^+$ 242.0, found 242.1; Rf = 0.64 (EtOAc/n-Hex, 2:1, v/v).

2-chloro-1-(3,4-dihydroquinolin-1(2H)-yl)ethan-1-one (***11x***)*:* Yield: quantitative; brown oil; ^{1}H NMR (400 MHz, $CDCl_3$): δ (ppm) = 1.96 – 2.03 (m, 2H), 2.72 – 2.76 (m, 2H), 3.83 (t, *J* = 6.7 Hz, 2H), 4.23 (s, 2H), 7.15 – 7.24 (m, 4H) as reported[20]; MS (ESI+) *m/z* calc. for $C_{11}H_{13}ClNO^+$ [M+H]$^+$ 210.1, found 210.1; Rf = 0.46 (EtOAc/n-Hex; 1:1; v/v).

*2-chloro-1-(3,4-dihydroisoquinolin-2(1H)-yl)ethan-1-one (**11y**):* Yield: 52%; yellow oil; according to NMR the compound is a mixture of isomers in ratio isomer A : isomer B = 2 : 1.3; ^{1}H NMR (400 MHz, $CDCl_3$): δ (ppm) = 2.89 (t, *J* = 6.0 Hz, 2H – isomer B), 2.97 (t, *J* = 5.9 Hz, 2H – isomer A), 3.75 (t, *J* = 5.9 Hz, 2H – isomer A), 3.84 (t, *J* = 6.0 Hz, 2H – isomer B), 4.16 (s, 2H – isomer A), 4.17 (s, 2H – isomer B), 4.69 (s, 2H – isomer B), 4.74 (s, 2H – isomer A), 7.10 – 7.23 (m, 4H – mixture of both isomers) as reported[21]; MS (ESI+): *m/z* calc. for $C_{11}H_{13}ClNO$ $[M+H]^+$ 210.1, found 210.0; *m/z* calc. for $C_{13}H_{16}ClN_2O$ $[M+MeCN+H]^+$ 251.1, found 251.0; Rf = 0.42 (EtOAc/n-hexane, 1:1, v/v).

*1.5 Final compounds (**12-53**):*

*5-(2-(3,4-dihydroquinolin-1(2H)-yl)-2-oxoethyl)-3-(4-fluorophenyl)isoxazolo[5,4-d]pyrimidin-4(5H)-one (**12**):* Yield: 46 %; white solid; Mp 192 – 193 °C; ^{1}H NMR (400 MHz, DMSO-d_6): δ (ppm) = 1.86 – 2.00 (m, 2H), 2.76 (t, *J* = 6.3 Hz, 2H), 3.72 – 3.81 (m, 2H), 5.12 (s, 2H), 7.10 – 7.32 (m, 3H), 7.37 – 7.43 (m, 2H), 7.47 – 7.67 (m, 1H), 8.25 – 8.34 (m, 2H), 8.71 (s, 1H); ^{13}C NMR (100 MHz, DMSO-d_6): δ (ppm) = 23.30, 26.20, 43.24, 48.39, 99.33, 115.99 (d, J_{C-F} = 21.9 Hz), 123.43 (d, J_{C-F} = 3.2 Hz), 124.13, 126.09, 128.84, 131.07 (d, J_{C-F} = 8.9 Hz), 137.55, 155.54, 156.43, 158.36, 163.77 (d, J_{C-F} = 249.2 Hz), 165.54, 175.45; HRMS (ESI+) m/z calc. for $C_{22}H_{18}FN_4O_5$ $[M+H]^+$ 405.1358, found 405.1357; HPLC purity 98.57 % at 254 nm (t_R = 10.080 min); Rf = 0.31 (EtOAc/n-Hex; 1:1, v/v).

*2-(3-(4-fluorophenyl)-4-oxoisoxazolo[5,4-d]pyrimidin-5(4H)-yl)-N,N-dimethylacetamide (**13**):* Yield: 45 %; white solid; Mp = 215 – 217 °C; ^{1}H NMR (400 MHz, DMSO-d_6): δ (ppm) = 2.87 (s, 3H), 3.10 (s, 3H), 5.00 (s, 2H), 7.38 – 7.45 (m, 2H), 8.30 – 8.35 (m, 2H), 8.67 (s, 1H); ^{13}C NMR (100 MHz, DMSO-d_6): δ (ppm) = 35.27, 35.85, 47.08, 99.32, 115.99 (d, J_{C-F} = 21.9 Hz), 123.48 (d, J_{C-F} = 3.1 Hz), 131.09 (d, J_{C-F} = 8.8 Hz), 155.57, 156.45, 158.37, 163.78 (d, J_{C-F} = 249.2 Hz), 165.61, 175.45; HRMS (ESI+) *m/z* calc. for $C_{15}H_{14}FN_4O_3$ $[M+H]^+$ 317.1044, found 317.1043; HPLC purity 99.10 % at 254 nm (t_R = 6.910 min); Rf = 0.12 (EtOAc/n-Hex; 1:1; v/v).

*3-(4-fluorophenyl)-5-(2-oxo-2-(piperidin-1-yl)ethyl)isoxazolo[5,4-d]pyrimidin-4(5H)-one (**14**):* Yield: 18 %; white solid; Mp 174 – 176 °C; ^{1}H NMR (400 MHz, DMSO-d_6): δ (ppm) 1.42 – 1.50 (m, 2H), 1.56 – 1.67 (m, 4H), 3.41 – 3.47 (m, 2H), 3.48 – 3.53 (m, 2H), 5.01 (s, 2H), 7.39 – 7.46 (m, 2H), 8.30 – 8.36 (m, 2H), 8.68 (s, 1H); ^{13}C NMR (100 MHz, DMSO-d_6): δ (ppm) = 23.84, 25.17, 25.90, 42.72, 45.19, 47.06, 99.30, 116.00 (d, J_{C-F} = 21.9 Hz), 123.49 (d, J_{C-F} = 3.1 Hz), 131.10 (d, J_{C-F} = 8.9 Hz), 155.64, 156.47, 158.38, 163.77 (d, J_{C-F} = 249.1 Hz), 163.89, 175.45; HRMS (ESI+) *m/z* calc. for $C_{18}H_{18}FN_4O_3$ $[M+H]^+$ 357.1357, found 357.1351; HPLC purity 98.96 % at 254 nm (t_R = 9.137 min); Rf = 0.15 (EtOAc/n-Hex; 2:1, v/v).

*N-(adamantan-1-yl)-2-(3-(4-fluorophenyl)-4-oxoisoxazolo[5,4-d]pyrimidin-5(4H)-yl)acetamide (**15**):* Yield: 18 %; white crystals; Mp 204 – 205 °C; ^{1}H NMR (400 MHz, DMSO-d_6): δ (ppm) = 1.59 – 1.62 (m, 6H), 1.91 – 1.93 (m, 6H), 1.98 – 2.02 (m, 3H), 4.67 (s, 2H), 7.40 – 7.46 (m, 2H), 7.94 (s, 1H), 8.30 – 8.35 (m, 2H), 8.68 (s, 1H); ^{13}C NMR (100 MHz, DMSO-d_6): δ (ppm) 28.77, 35.99, 41.02, 48.42, 51.30, 99.27, 116.01 (d, J_{C-F} = 22.0 Hz), 123.55 (d, J_{C-F} = 3.2 Hz), 131.11 (d, J_{C-F} = 8.8 Hz), 155.77, 156.47, 158.37, 163.77 (d, J_{C-F} = 249.1 Hz), 164.66, 175.43; HRMS (ESI+) *m/z* calc. for $C_{23}H_{24}N_4O_3F$ $[M+H]^+$ 423.1827, found 423.1824; HPLC purity 96.06 % at 254 nm (t_R = 11.633 min); Rf = 0.41 (EtOAc/n-Hex, 1:1, v/v).

*2-(3-(4-fluorophenyl)-4-oxoisoxazolo[5,4-d]pyrimidin-5(4H)-yl)-N-phenylacetamide (**16**):* Yield: 55 %; white solid; Mp 203 – 205 °C; ^{1}H NMR (400 MHz, DMSO-d_6): δ (ppm) 4.95 (s, 2H), 7.05 – 7.10 (m, 1H), 7.30 – 7.35 (m, 2H), 7.38 – 7.44 (m, 2H), 7.55 – 7.60 (m, 2H), 8.30 – 8.35 (m, 2H), 8.80 (s, 1H), 10.50 (s, 1H); ^{13}C NMR (100 MHz, DMSO-d_6): δ (ppm) 49.12, 99.37, 115.98 (d, J_{C-F} = 21.9 Hz), 119.08, 123.43 (d, J_{C-F} = 3.2 Hz), 123.71, 128.92, 131.08 (d, J_{C-F} = 8.8 Hz), 138.46, 155.63, 156.55, 158.37, 163.75 (d, J_{C-F} = 249.1 Hz), 164.71, 175.48; HRMS (ESI-) *m/z* calc. for $C_{19}H_{12}FN_4O_3^-$ $[M-H]^-$ 363.0892, found 363.0893; HPLC purity 97.04 % at 254 nm (t_R = 8.907 min); Rf = 0.47 (EtOAc/n-Hex, 2:1, v/v).

*N-(4-fluorophenyl)-2-(3-(4-fluorophenyl)-4-oxoisoxazolo[5,4-d]pyrimidin-5(4H)-yl)acetamide (**17**):* Yield: 14 %; white solid; Mp 206 – 209 °C; ^{1}H NMR (400 MHz, DMSO-d_6): δ (ppm) = 4.94 (s, 2H), 7.14 – 7.20 (m, 2H), 7.38 – 7.44 (m, 2H), 7.57 – 7.62 (m, 2H), 8.30 – 8.35 (m, 2H), 8.79 (s, 1H), 10.56 (s, 1H); ^{13}C NMR (100 MHz, $CDCl_3$): δ (ppm) = 49.06, 99.38, 115.53 (d, J_{C-F} = 22.3 Hz), 115.99 (d, J_{C-F} = 21.9 Hz), 120.89 (d, J_{C-F} = 7.9 Hz), 123.44 (d, J_{C-F} = 3.1 Hz), 131.09 (d, J_{C-F} = 8.9 Hz), 134.85 (d, J_{C-F} = 2.6 Hz), 155.62, 156.55, 158.20 (d, J_{C-F} = 240.1 Hz), 158.38, 163.75 (d, J_{C-F} = 249.1 Hz), 164.67, 175.48; HRMS (ESI+) *m/z* calc. for $C_{19}H_{13}O_3N_4F_2$ 383.0950 $[M+H]^+$, found 383.0945; HPLC purity 96.38 % at 254 nm (t_R = 9.053 min); Rf = 0.50 (EtOAc/DCM, 10:7, v/v).

*Methyl 2-(2-(3-(4-fluorophenyl)-4-oxoisoxazolo[5,4-d]pyrimidin-5(4H)-yl)acetamido)benzoate (**18**):* Yield: 19 %; red solid; Mp 212 – 214 °C; ^{1}H NMR (400 MHz, DMSO-d_6): δ (ppm) = 3.81 (s, 3H), 5.02 (s, 2H), 7.25 (ddd, J_1 = 1.2, J_2 = 7.4, J_3 = 8.0 Hz, 1H), 7.39 – 7.45 (m, 2H), 7.62 (ddd, J_1 = 1.6, J_2 = 7.4, J_3 = 8.3 Hz, 1H), 7.90 (dd, J_1 = 1.6, J_2 = 7.9 Hz, 1H), 8.07 (dd, J_1 = 0.9, J_2 = 8.5 Hz, 1H), 8.30 – 8.35 (m, 2H), 8.84 (s, 1H), 10.83 (s, 1H); ^{13}C NMR (100 MHz, DMSO-d_6): δ (ppm) = 49.63, 52.44, 99.51, 116.04 (d, $J_{C\text{-}F}$ = 22.0 Hz), 119.31, 122.00, 123.44 (d, $J_{C\text{-}F}$ = 3.3 Hz), 124.18, 130.60, 131.10 (d, $J_{C\text{-}F}$ = 8.8 Hz), 133.85, 138.21, 155.54, 156.56, 158.42, 163.79 (d, $J_{C\text{-}F}$ = 249.1 Hz), 165.17, 167.34, 175.50; HRMS (ESI-) *m/z* calc. for $C_{21}H_{14}FN_4O_5^-$ [M-H]$^-$ 421.0954, found 421.0956; HPLC purity 95.16 % at 254 nm (t_R = 10.003 min); Rf = 0.36 (EtOAc/n-Hex; 1:1, v/v).

*Methyl 3-(2-(3-(4-fluorophenyl)-4-oxoisoxazolo[5,4-d]pyrimidin-5(4H)-yl)acetamido)benzoate (**19**):* Yield: 6 %; white solid; Mp 225 – 227 °C; ^{1}H NMR (400 MHz, DMSO-d_6): δ (ppm) = 3.84 (s, 3H), 4.97 (s, 2H), 7.38 – 7.44 (m, 2H), 7.49 (t, J = 7.9 Hz, 1H), 7.66 – 7.69 (m, 1H), 7.80 (ddd, J_1 = 1.0, J_2 = 2.1, J_3 = 8.1 Hz, 1H), 8.28 (t, J = 1.8 Hz, 1H), 8.30 – 8.35 (m, 2H), 8.81 (s, 1H), 10.75 (s, 1H); ^{13}C NMR (100 MHz, DMSO-d_6): δ (ppm) = 49.22, 52.26, 99.41, 116.00 (d, $J_{C\text{-}F}$ = 22.2 Hz), 119.60, 123.45 (d, $J_{C\text{-}F}$ = 3.2 Hz), 123.55, 124.34, 129.51, 130.27, 131.10 (d, $J_{C\text{-}F}$ = 8.8 Hz), 138.83, 155.61, 156.58, 158.39, 163.78 (d, $J_{C\text{-}F}$ = 249.2 Hz), 165.15, 165.95, 175.51; HRMS (ESI-) *m/z* calc. for $C_{21}H_{14}FN_4O_5^-$ [M-H]$^-$ 421.0954, found 421.0955; HPLC purity 96.65 % at 254 nm (t_R = 9.627 min); Rf = 0.16 (EtOAc/n-Hex; 1:1, v/v).

*Methyl 4-(2-(3-(4-fluorophenyl)-4-oxoisoxazolo[5,4-d]pyrimidin-5(4H)-yl)acetamido)benzoate (**20**):* Yield: 27 %; white solid; Mp 234 – 236 °C; ^{1}H NMR (400 MHz, DMSO-d_6): δ (ppm) = 3.82 (s, 3H), 4.99 (s, 2H), 7.38 – 7.44 (m, 2H), 7.70 – 7.74 (m, 2H), 7.93 – 7.96 (m, 2H), 8.30 – 8.35 (m, 2H), 8.80 (s, 1H), 10.86 (s, 1H); ^{13}C NMR (100 MHz, DMSO-d_6): δ (ppm) = 49.31, 51.69, 99.40, 116.00 (d, $J_{C\text{-}F}$ = 22.0 Hz), 118.61, 123.43 (d, $J_{C\text{-}F}$ = 3.1 Hz), 124.48, 130.47, 131.09 (d, $J_{C\text{-}F}$ = 8.9 Hz), 142.78, 155.58, 156.55, 158.39, 163.77 (d, $J_{C\text{-}F}$ = 249.2 Hz), 165.41, 165.74, 175.50; HRMS (ESI-) *m/z* calc. for $C_{21}H_{14}FN_4O_5^-$ [M-H]$^-$ 421.0954, found 421.0955; HPLC purity 95.73 % at 254 nm (t_R = 8.963 min); Rf = 0.14 (EtOAc/n-Hex; 1:1, v/v).

*Methyl 4-((2-(3-(4-fluorophenyl)-4-oxoisoxazolo[5,4-d]pyrimidin-5(4H)-yl)acetamido)methyl)benzoate (**21**):* Yield: 6 %; white solid; Mp 165 – 167 °C; ^{1}H NMR (400 MHz, DMSO-d_6): δ (ppm) = 3.84 (s, 3H), 4.41 (d, J = 5.8 Hz, 2H), 4.82 (s, 2H), 7.41 – 7.47 (m, 4H), 7.91 – 7.93 (m, 2H), 8.31 – 8.36 (m, 2H), 8.77 (s, 1H), 8.95 (t, J = 5.9 Hz, 1H); ^{13}C NMR (100 MHz, DMSO-d_6): δ (ppm) = 42.07, 48.64, 52.14, 99.47, 116.04 ($J_{C\text{-}F}$ = 21.9 Hz), 123.51 ($J_{C\text{-}F}$ = 3.2 Hz), 127.48, 128.30, 129.25, 131.10 ($J_{C\text{-}F}$ = 8.8 Hz), 144.59, 155.60, 156.61, 158.39, 163.81 ($J_{C\text{-}F}$ = 249.3 Hz), 166.09, 166.31, 175.51; HRMS (ESI+) *m/z* calc. for $C_{22}H_{18}FN_4O_5$ [M+H]+ 437.1256, found 437.1253; HPLC purity 91.37 % at 254 nm (t_R = 8.427 min); Rf = 0.30 (EtOAc/DCM; 10:7, v/v).

*2-(3-(4-fluorophenyl)-4-oxoisoxazolo[5,4-d]pyrimidin-5(4H)-yl)-N-(3-(trifluoromethyl)phenyl)acetamide (**22**):* Yield: 76 %; white solid; Mp 195 – 200°C; ^{1}H NMR (400 MHz, DMSO-d_6): δ (ppm) = 4.98 (s, 2H), 7.38 – 7.43 (m, 2H), 7.43 – 7.46 (m, 1H), 7.59 (t, J = 8.0 Hz, 1H), 7.75 (ddd, J_1 = 0.5 Hz, J_2 = 1.1 Hz, J_3 = 8.3 Hz, 1H), 8.08 – 8.09 (m, 1H), 8.30 – 8.35 (m, 2H), 8.81 (s, 1H), 10.88 (s, 1H); ^{13}C NMR (400 MHz, DMSO-d_6): δ (ppm) = 49.25, 99.42, 115.17 (q, $J_{C\text{-}F}$ = 4.5 Hz), 116.02 (d, $J_{C\text{-}F}$ = 22.0 Hz), 120.16 (q, $J_{C\text{-}F}$ = 4.4 Hz), 122.70, 123.40 (d, $J_{C\text{-}F}$ = 3.0 Hz), 124.04 (q, $J_{C\text{-}F}$ = 272.0 Hz), 129.63 (q, $J_{C\text{-}F}$ = 31.9 Hz), 130.31, 131.11 (d, $J_{C\text{-}F}$ = 8.9 Hz), 139.20, 155.61, 156.58, 158.41, 163.79 (d, $J_{C\text{-}F}$ = 249.1 Hz), 165.46, 175.52; HRMS (ESI+) *m/z* calc. for $C_{20}H_{13}F_4N_4O_3$ [M+H]$^+$ 433.0918, found 433.0918; HPLC purity 98.66 % at 254 nm (t_R = 10.190 min); Rf = 0.14 (EtOAc/n-Hex; 1:1, v/v).

*2-(3-(4-fluorophenyl)-4-oxoisoxazolo[5,4-d]pyrimidin-5(4H)-yl)-N-(4-(trifluoromethyl)phenyl)acetamide (**23**):* Yield: 14 %; white crystals; Mp 225 – 227 °C; ^{1}H NMR (400 MHz, DMSO-d_6): δ (ppm) = 4.99 (s, 2H), 7.35 – 7.45 (m, 2H), 7.67 – 7.83 (m, 4H), 8.28 – 8.37 (m, 2H), 8.81 (s, 1H), 10.88 (s, 1H); ^{13}C NMR (100 MHz, DMSO-d_6): δ (ppm) = 49.26, 99.39, 115.98 (d, $J_{C\text{-}F}$ = 22.0 Hz), 119.09, 123.42 (d, $J_{C\text{-}F}$ = 3.2 Hz), 123.75 (q, $J_{C\text{-}F}$ = 32.2 Hz), 124.28 (q, $J_{C\text{-}F}$ = 271.2 Hz), 126.28 (q, $J_{C\text{-}F}$ = 3.8 Hz), 131.07 (d, $J_{C\text{-}F}$ = 8.8 Hz), 141.97 (q, $J_{C\text{-}F}$ = 1.4 Hz), 155.57, 156.54, 158.37, 163.76 (d, $J_{C\text{-}F}$ = 249.2 Hz), 165.46, 175.25; HRMS (ESI-): *m/z* calc. for $C_{20}H_{11}F_4N_4O_3$ [M-H]$^-$ 431.0773, found 431.0772; HPLC purity 97.58 % at 254 nm (t_R = 10.233 min); Rf = 0.45 (EtOAc/n-Hex, 2:1, v/v).

*2-(3-(4-fluorophenyl)-4-oxoisoxazolo[5,4-d]pyrimidin-5(4H)-yl)-N-(3-nitrophenyl)acetamide (**24**):* Yield: 10 %; light brown solid; Mp 232 – 235°C; ^{1}H NMR (400 MHz, DMSO-d_6): δ (ppm) = 4.99 (s, 2H), 7.38 – 7.44 (m, 2H), 7.65 (t, J = 8.2 Hz, 1H), 7.89 (ddd, J_1 = 0.8 Hz, J_2 = 2.1 Hz, J_3 = 8.2 Hz, 1H), 7.95 (ddd, J_1 = 0.8 Hz, J_2 = 2.3 Hz, J_3 = 8.2 Hz, 1H), 8.29 – 8.34 (m, 2H), 8.59 (t, J = 2.2 Hz, 1H), 8.80 (s, 1H), 11.02 (s, 1H); ^{13}C NMR (100 MHz, DMSO-d_6): δ (ppm) = 49.33, 99.47, 113.35, 116.06 (d, $J_{C\text{-}F}$ = 22.0 Hz), 118.41, 123.46 (d, $J_{C\text{-}F}$ = 3.1 Hz), 125.19, 130.56, 131.15 (d, $J_{C\text{-}F}$ = 8.9 Hz), 139.53, 148.07, 155.62, 156.62, 158.46, 163.83 (d, $J_{C\text{-}F}$ = 249.2

Hz), 165.68, 175.55; HRMS (ESI-) *m/z* calc. for $C_{19}H_{11}FN_5O_5$ [M-H]$^-$ 408.0750, found 408.0751; HPLC purity 96.54 % at 254 nm (t_R = 9.083 min); Rf = 0.34 (EtOAc/n-Hex; 2:1, v/v).

*2-(3-(4-fluorophenyl)-4-oxoisoxazolo[5,4-d]pyrimidin-5(4H)-yl)-N-(4-nitrophenyl)acetamide (**25**):* Yield: 39 %; yellow solid; Mp 200 – 203 °C; ^{1}H NMR (400 MHz, DMSO-d_6): δ (ppm) = 5.01 (s, 2H), 7.39 – 7.45 (m, 2H), 7.81 – 7.85 (m, 2H), 8.24 – 8.27 (m, 2H), 8.30 – 8.35 (m, 2H), 8.81 (s, 1H), 11.13 (s, 1H); ^{13}C NMR (100 MHz, DMSO-d_6): δ (ppm) = 49.46, 99.46, 116.06 (d, $J_{C\text{-}F}$ = 22.5 Hz), 119.03, 123.43, 125.22, 131.14 ($J_{C\text{-}F}$ = 8.6 Hz), 142.66, 144.53, 155.59, 156.60, 163.52, 163.84 (d, $J_{C\text{-}F}$ = 248.5 Hz), 165.90, 175.54; HRMS (ESI+) *m/z* calc. for $C_{19}H_{13}O_5N_5F$ 410.0895 [M+H]$^+$, found 410.0894; HPLC purity 98.20 % at 254 nm (t_R = 9.180 min); Rf = 0.40 (EtOAc/DCM, 10:7, v/v).

*2-(3-(4-fluorophenyl)-4-oxoisoxazolo[5,4-d]pyrimidin-5(4H)-yl)-N-(4-(methylsulfonyl)phenyl)acetamide (**26**):* Yield: 35 %; white crystals; Mp 234 – 238 °C; ^{1}H NMR (400 MHz, DMSO-d_6): δ (ppm) = 3.17 (s, 3H), 5.00 (s, 2H), 7.39 – 7.44 (m, 2H), 7.95 – 7.83 (m, 2H), 7.88 – 7.91 (m, 2H), 8.30 – 8.35 (m, 2H), 8.81 (s, 1H), 10.97 (s, 1H); ^{13}C NMR (100 MHz, DMSO-d_6): δ (ppm) = 43.78, 49.35, 99.43, 116.02 ($J_{C\text{-}F}$ = 21.8 Hz), 119.05, 123.43 ($J_{C\text{-}F}$ = 3.2 Hz), 128.45, 131.10 ($J_{C\text{-}F}$ = 8.9 Hz), 135.25, 142.91, 155.59, 156.58, 158.40, 163.79 ($J_{C\text{-}F}$ = 249.2 Hz), 165.65,175.51; HRMS (ESI+) *m/z* calc. for $C_{20}H_{16}O_5N_4FS$ 443.0820 [M+H]$^+$, found 443.0819; HPLC purity 98.56 % at 254 nm (t_R = 7.707 min); Rf = 0.20 (EtOAc/DCM, 10:7, v/v).

*N-(4-cyanophenyl)-2-(3-(4-fluorophenyl)-4-oxoisoxazolo[5,4-d]pyrimidin-5(4H)-yl)acetamide (**27**)*: Yield: 53 %; yellow solid; Mp > 250 °C; ^{1}H NMR (400 MHz, DMSO-d_6): δ (ppm) = 4.99 (s, 2H), 7.38 – 7.44 (m, 2H), 7.74 – 7.77 (m, 2H), 7.80 – 7.83 (m, 2H), 8.29 – 8.35 (m, 2H), 8.80 (s, 1H), 10.95 (s, 1H); ^{13}C NMR (100 MHz, DMSO-d_6): δ (ppm) = 49.32, 99.38, 105.57, 115.99 (d, $J_{C\text{-}F}$ = 21.9 Hz), 118.89, 119.21, 123.4 (d, $J_{C\text{-}F}$ = 3.1 Hz), 131.08 (d, $J_{C\text{-}F}$ = 8.8 Hz), 133.50, 142.57, 155.55, 156.52, 158.37, 163.76 (d, $J_{C\text{-}F}$ = 249.2 Hz), 165.63, 175.47; HRMS (ESI+) *m/z* calc. for $C_{20}H_{11}O_3N_5F$ 388.0851 [M-H]$^-$, found 388.0846; HPLC purity 97.36 % at 254 nm (t_R = 8.673 min); Rf = 0.40 (EtOAc/ n-Hex; 9:1; v/v).

*N-(4-(dimethylamino)phenyl)-2-(3-(4-fluorophenyl)-4-oxoisoxazolo[5,4-d]pyrimidin-5(4H)-yl)acetamide (**28**):* Yield: 82 %; green crystals; Mp 249 – 250 °C; ^{1}H NMR (400 MHz, DMSO-d_6): δ (ppm) = 2.84 (s, 6H), 4.90 (s, 2H), 6.67 – 6.71 (m, 2H), 7.37 – 7.44 (m, 4H), 8.30 – 8.36 (m, 2H), 8.78 (s, 1H), 10.20 (s, 1H); ^{13}C NMR (100 MHz, DMSO-d_6): δ (ppm) = 40.43, 48.96, 99.41, 112.67, 116.02 ($J_{C\text{-}F}$ = 21.9 Hz), 120.54, 123.50 ($J_{C\text{-}F}$ = 3.1 Hz), 128.21, 131.14($J_{C\text{-}F}$ = 8.9 Hz), 147.31, 155.70, 156.60, 158.43, 163.79, 163.80 ($J_{C\text{-}F}$ = 249.2 Hz), 175.52; HRMS (ESI+): *m/z* calc. for $C_{21}H_{19}FN_5O_3$ [M+H]$^+$ 408.1466, found 408.1458; HPLC purity 99.70 % at 254 nm (t_R = 5.897 nm); Rf = 0.20 (EtOAc/n-Hex, 2:1, v/v).

*2-(3-(4-fluorophenyl)-4-oxoisoxazolo[5,4-d]pyrimidin-5(4H)-yl)-N-(4-(oxazol-2-yl)phenyl)acetamide (**29**):* Yield: 15 %; pale yellow solid; Mp 237 – 239 °C; ^{1}H NMR (400 MHz, DMSO-d_6): δ (ppm) = 4.97 (s, 2H), 7.38 – 7.44 (m, 2H), 7.60 (s, 1H), 7.70 (s, 4H), 8.31 – 8.36 (m, 2H), 8.41 (s, 1H), 8.80 (s, 1H), 10.69 (s, 1H); ^{13}C NMR (100 MHz, DMSO-d_6): δ (ppm) = 49.19, 99.39, 115.97 (d, $J_{C\text{-}F}$ = 22.0 Hz), 119.45, 121.25, 122.83, 123.43 (d, $J_{C\text{-}F}$ = 3.1 Hz), 124.91, 131.08 (d, $J_{C\text{-}F}$ = 9.0 Hz), 138.70, 150.33, 151.50, 155.60, 156.55, 158.37, 163.76 (d, $J_{C\text{-}F}$ = 249.1 Hz), 164.95, 175.49; HRMS (ESI+) *m/z* calc. for $C_{22}H_{15}O_4N_5F$ 432.1114 [M+H]$^+$, found 432.1113; HPLC purity 94.76 % at 254 nm (t_R = 8.283 min); Rf = 0.30 (EtOAc/DCM, 1:2, v/v).

*N-(4-(2H-tetrazol-5-yl)phenyl)-2-(3-(4-fluorophenyl)-4-oxoisoxazolo[5,4-d]pyrimidin-5(4H)-yl)acetamide (**30**):* To a solution of **27** (1.0 equiv.) in anhydrous DMF NH_4Cl (3.0 equiv.) and NaN_3 (3.0 equiv.) were added. The reaction mixture was stirred under inert atmosphere at 110 °C for 72 hours. 1 M HCl was added dropwise to a cooled reaction mixture until the formation of precipitate. The product was collected by filtration under reduced pressure and recrystallized from EtOH. Yield: 80 %; pale yellow solid; Mp 236 – 240 °C; ^{1}H NMR (400 MHz, DMSO-d_6): δ (ppm) = 5.00 (s, 2H), 7.38 – 7.44 (m, 2H), 7.79 – 7.82 (m, 2H), 7.99 – 8.03 (m, 2H), 8.30 – 8.35 (m, 2H), 8.82 (s, 1H), 10.88 (s, 1H), 1H from NH is exchanged; ^{13}C NMR (100 MHz, DMSO-d_6): δ (ppm) = 49.31, 99.43, 116.04 (d, $J_{C\text{-}F}$ = 22.0 Hz), 119.51, 123.46 (d, $J_{C\text{-}F}$ = 2.9 Hz), 127.99, 131.13 (d, $J_{C\text{-}F}$ = 8.8 Hz), 141.01, 155.64, 156.60, 158.42, 162.36; 163.80 (d, $J_{C\text{-}F}$ = 249.2 Hz), 165.33, 175.53; HRMS (ESI+) *m/z* calc. for $C_{20}H_{12}O_3N_8F$ 431.1022 [M-H]$^-$, found 431.1015; HPLC purity 95.87 % at 254 nm (t_R = 7.203 min).

*2-(3-(4-fluorophenyl)-4-oxoisoxazolo[5,4-d]pyrimidin-5(4H)-yl)-N-(4-isopropylphenyl)acetamide (**31**):* Yield: 10 %; white solid; Mp 198 – 202 °C; ^{1}H NMR (400 MHz, DMSO-d_6): δ (ppm) = 1.17 (d, *J* = 6.9 Hz, 6H),2.83 (q, *J* = 6.9 Hz, 1H), 4.94 (s, 2H), 7.17 – 7.21 (m, 2H), 7.38 – 7.44 (m, 2H), 7.47 – 7.51 (m, 2H), 8.30 – 8.35 (m, 2H), 8.80 (s, 1H), 10.48 (s, 1H); ^{13}C NMR (100 MHz, DMSO-d_6): δ (ppm) = 23.95, 32.88, 49.11, 99.40,

116.02 (d, J_{C-F} = 21.9 Hz), 119.19, 123.48 (d, J_{C-F} = 3.1 Hz), *125.29*, 126.64, *126.79*, 131.12 (d, J_{C-F} = 8.8 Hz), 136.27, 143.78, 155.68, 156.58, 158.40, 163.78 (d, J_{C-F} = 249.1 Hz), *164.02*, 164.51, 175.51; HRMS (ESI+) *m/z* calc. for $C_{22}H_{20}O_3N_4F$ 407.1514 [M+H]$^+$, found 407.1507; HPLC purity 95.52 % at 254 nm (t_R = 10.597 min); Rf = 0.20 (EtOAc/n-Hex, 1:1, v/v).

*N-(4-cyclohexylphenyl)-2-(3-(4-fluorophenyl)-4-oxoisoxazolo[5,4-d]pyrimidin-5(4H)-yl)acetamide (**32**):* Yield: 31 %; white crystals; Mp 204 – 205 °C; ^{1}H NMR (400 MHz, DMSO-*d6*): δ (ppm) = 1.15 – 1.25 (m, 1H), 1.29 – 1.41 (m, 4H), 1.65 – 1.71 (m, 1H), 1.72 – 1.82 (m, 4H), 2.41 – 2.47 (m, 1H), 4.93 (s, 2H), 7.14 – 7.19 (m, 2H), 7.37 – 7.44 (m, 2H), 7.45 – 7.49 (m, 2H), 8.30 – 8.35 (m, 2H), 8.79 (s, 1H), 10.41 (s, 1H); ^{13}C NMR (100 MHz, DMSO-*d6*): δ (ppm) = 25.58, 26.34, 34.01, 43.15, 49.07, 99.36, 115.96 (d, J_{C-F} = 21.9 Hz), 119.16, 123.45 (d, J_{C-F} = 3.1 Hz), 126.95, 131.07 (d, J_{C-F} = 8.8 Hz), 136.23, 143.02, 155.62, 156.53, 158.36, 163.75 (d, J_{C-F} = 249.1 Hz), 164.45, 175.48; HRMS (ESI-): *m/z* calc. for $C_{25}H_{22}FN_4O_3$ [M-H]$^-$ 445.1681, found 445.1686; HRMS (ESI+): *m/z* calc. for $C_{25}H_{24}FN_4O_3$ [M+H]$^+$ 447.1827, found 447.1831; HPLC purity 100.00 % at 254 nm (t_R = 11.980 nm); Rf = 0.43 (EtOAc/n-Hex, 2:1, v/v).

*N-(4-butoxyphenyl)-2-(3-(4-fluorophenyl)-4-oxoisoxazolo[5,4-d]pyrimidin-5(4H)-yl)acetamide (**33**):* Yield: 32 %; white crystals; Mp 171 – 173 °C; ^{1}H NMR (400 MHz, DMSO-*d6*): δ (ppm) = 0.92 (t, *J* = 7.4 Hz, 3H), 1.37 – 1.46 (m, 2H), 1.63 – 1.70 (m, 2H), 3.92 (t, *J* = 6.5 Hz, 2H), 4.91 (s, 2H), 6.87 – 6.90 (m, 2H), 7.38 – 7.44 (m, 2H), 7.45- 7.49 (m, 2H), 8.30 – 8.35 (m, 2H), 8.79 (s, 1H), 10.33 (s, 1H); ^{13}C NMR (100 MHz, DMSO-*d6*): δ (ppm) = 13.76, 18.81, 30.85, 49.06, 67.33, 99.45, 114.63, 116.03 (d, J_{C-F} = 21.8 Hz), 120.72, 123.51 (d, J_{C-F} = 3.0 Hz), 131.16 (d, J_{C-F} = 8.8 Hz), 131.52, 155.02, 155.69, 156.63, 158.45, 163.83 (d, J_{C-F} = 249.4 Hz), 164.26, 175.55; HRMS (ESI+/-): *m/z* calc. for $C_{23}H_{22}FN_4O_4$ [M+H]$^+$ 437.1620, found 437.1615; *m/z* calc. for $C_{23}H_{20}FN_4O_4$ [M-H]$^-$ 435.1474, found 435.1473; HPLC purity 97.23 % at 254 nm (t_R = 10.770 min); Rf = 0.62 (EtOAc/n-Hex, 2:1, v/v).

*2-(3-(4-fluorophenyl)-4-oxoisoxazolo[5,4-d]pyrimidin-5(4H)-yl)-N-(3,4,5-trimethoxyphenyl)acetamide (**34**):* Yield: 74 %; white crystals; Mp 219 – 221 °C; ^{1}H NMR (400 MHz, DMSO-*d6*): δ (ppm) = 3.61 (s, 3H), 3.72 (s, 6H), 4.92 (s, 2H), 6.95 (s, 2H), 7.40 – 7.44 (m, 2H), 8.31 – 8.35 (m, 2H), 8.79 (s, 1H), 10.48 (s, 1H); ^{13}C NMR (100 MHz, DMSO-*d6*): δ (ppm) = 49.23, 55.79, 60.24, 96.84, 99.49, 116.08 (J_{C-F} = 21.9 Hz), 123.53 (J_{C-F} = 3.0 Hz), 131.20 (J_{C-F} = 8.8 Hz), 133.74, 134.69, 152.93, 155.69, 156.65, 158.50, 163.88 (J_{C-F} = 249.3 Hz), 164.72, 175.59; HRMS (ESI+): *m/z* calc. for $C_{22}H_{20}O_6N_4F$ [M+H]$^+$ 455.1361, found 455.1360; HRMS (ESI-): *m/z* calc. for $C_{22}H_{18}O_6N_4F$ [M-H]$^-$ 453.1216, found 453.1215; HPLC purity 97.94 % at 254 nm (t_R = 8.473 nm); Rf = 0.14 (EtOAc/n-Hex, 2:1, v/v).

*2-(3-(4-fluorophenyl)-4-oxoisoxazolo[5,4-d]pyrimidin-5(4H)-yl)-N-(naphthalen-1-yl)acetamide (**35**):* Yield: 12 %; white solid; Mp 210 – 215 °C; ^{1}H NMR (400 MHz, DMSO-*d6*): δ (ppm) = 5.14 (s, 2H), 7.41 – 7.47 (m, 2H), 7.50 (t, *J* = 7.9 Hz, 1H), 7.54 – 7.62 (m, 2H), 7.68 (dd, J_1 = 0.7 Hz, J_2 = 7.3 Hz, 1H), 7.80 (d, *J* = 8.3 Hz, 1H), 7.95 – 7.97 (m, 1H), 8.16 – 8.18 (m, 1H), 8.34 – 8.38 (m, 2H), 8.87 (s, 1H), 10.47 (s, 1H); ^{13}C NMR (100 MHz, DMSO-*d6*): δ (ppm) = 49.12, 99.48, 116.05 (J_{C-F} = 21.8 Hz), 121.63, 122.69, 123.51 (J_{C-F} = 2.9 Hz), 125.61, 125.75, 126.05, 126.23, 127.64, 128.24, 131.11 (J_{C-F} = 8.7 Hz), 132.81, 133.74, 155.72, 156.70, 158.41, 163.80 (J_{C-F} = 249.4 Hz), 165.75, 175.54; HRMS (ESI+) *m/z* calc. for $C_{23}H_{16}O_3N_4F$ 415.1212 [M+H]$^+$, found 415.1191; HPLC purity 94.64 % at 254 nm (t_R = 9.467 min); Rf = 0.54 (EtOAc/DCM, 1:1, v/v).

*2-(3-(4-fluorophenyl)-4-oxoisoxazolo[5,4-d]pyrimidin-5(4H)-yl)-N-(naphthalen-2-yl)acetamide (**36**):* Yield: 63 %; white solid; Mp 180 – 184 °C; ^{1}H NMR (400 MHz, DMSO-*d6*): δ (ppm) = 5.02 (s, 2H), 7.38 – 7.44 (m, 3H), 7.47 (ddd, J_1 = 1.4 Hz, J_2 = 6.9 Hz, J_3 = 8.2 Hz, 1H), 7.80 (dd, J_1 = 0.5 Hz, J_2 = 8.2 Hz, 1H), 7.85 (dd, J_1 = 0.5 Hz, J_2 = 7.9 Hz, 1H), 7.90 (d, *J* = 8.9 Hz, 1H), 8.27 (d, *J* = 1.9 Hz, 1H), 8.31 – 8.36 (m, 2H), 8.84 (s, 1H), 10.73 (s, 1H); ^{13}C NMR (100 MHz, DMSO-*d6*): δ (ppm) = 49.31, 99.47, 115.51, 116.02 (J_{C-F} = 22.0 Hz), 119.72, 123.49 (J_{C-F} = 3.0 Hz), 124.93, 126.64, 127.42, 127.57, 128.72, 129.99, 131.14 (J_{C-F} = 8.8 Hz), 133.43, 136.09, 155.68, 156.66, 158.44, 163.83 (J_{C-F} = 249.3 Hz), 165.12, 175.57; HRMS (ESI+) *m/z* calc. for $C_{23}H_{16}O_3N_4F$ 415.1212 [M+H]$^+$, found 415.1206; HPLC purity 97.10 % at 254 nm (t_R = 10.027 min); Rf = 0.50 (EtOAc/DCM, 1:2, v/v).

*5-(2-(3,4-dihydroisoquinolin-2(1H)-yl)-2-oxoethyl)-3-(4-fluorophenyl)isoxazolo[5,4-d]pyrimidin-4(5H)-one (**37**):* Yield: 19 %; white solid; Mp 201 – 203 °C; according to NMR the compound is a mixture of isomers in ratio isomer A : isomer B = 2.0 : 1.3; ^{1}H NMR (400 MHz, DMSO-*d6*): δ (ppm) = 2.81 (t, *J* = 5.9 Hz, 2H, isomer B), 2.98 (t, *J* = 5.8 Hz, 2H, isomer A), 3.70 (t, *J* = 5.9 Hz, 2H, isomer B), 3.82 (t, *J* = 5.9 Hz, 2H, isomer A), 4.63 (s, 2H, isomer A), 4.81 (s, 2H, isomer B), 5.12 (s, 2H, isomer A), 5.14 (s, 2H, isomer B), 7.19 – 7.27 (m, 4H, mixture of both isomers), 7.38 – 7.45 (m, 2H, mixture of both isomers), 8.30 – 8.35 (m,

2H, mixture of both isomers), 8.70 (s, 1H, isomer B), 8.71 (s, 1H, isomer A); ^{13}C NMR (100 MHz, DMSO-*d*$_6$): δ (ppm) = 28.49, 42.04, 44.04 (45.51)*, 47.30, 99.38, 116.03 (d, $J_{C\text{-}F}$ = 21.9 Hz), 123.49 (d, $J_{C\text{-}F}$ = 2.9 Hz), 126.31, 126.36 (126.48)*, 126.65 (126.78)*, 128.48 (128.66)*, 131.13 (d, $J_{C\text{-}F}$ = 8.6 Hz), 132.67, 133.18, 134.43 (134.62)*, 155.62, 156.51, 158.42, 163.79 (d, $J_{C\text{-}F}$ = 249.0 Hz), 164.78, 175.48; * - 2nd isomer. HRMS (ESI+) *m/z* calc. for $C_{22}H_{18}N_4O_3F$ $[M+H]^+$ 405.1357, found 405.1354; HPLC purity 100.00 % at 254 nm (t_R = 9.550 min); Rf = 0.21 (EtOAc/n-hexane, 1:1, v/v).

*5-(2-(3,4-dihydroquinolin-1(2H)-yl)-2-oxoethyl)-3-phenylisoxazolo[5,4-d]pyrimidin-4(5H)-one (**38**):* Yield: 39 %; white solid; Mp 210 – 212 °C; ^{1}H NMR (400 MHz, DMSO-*d*$_6$): δ (ppm) = 1.87 – 1.97 (m, 2H), 2.77 (t, *J* = 6.3 Hz, 2H), 3.73 – 3.80 (m, 2H), 5.12 (s, 2H), 7.13 – 7.30 (m, 3H), 7.53 – 7.63 (m, 4H), 8.18 – 8.24 (m, 2H), 8.71 (s, 1H); ^{13}C NMR (100 MHz, DMSO-*d*$_6$): δ (ppm) = 23.32, 26.21, 48.42, 99.41, 124.16, 126.89, 128.56, 128.87, 131.21, 137.57, 155.51, 156.39, 159.33, 165.60, 175.46; HRMS (ESI-) *m/z* calc. for $C_{22}H_{17}N_4O_3$ $[M\text{-}H]^-$ 385.1307, found 385.1306; HPLC purity 94.00 % at 254 nm (t_R = 9.750 min); Rf = 0.53 (EtOAc/n-Hex; 2:1, v/v).

*Methyl 4-(2-(4-oxo-3-phenylisoxazolo[5,4-d]pyrimidin-5(4H)-yl)acetamido)benzoate (**39**):* Yield: 41 %; white solid; Mp 200 – 202 °C; ^{1}H NMR (400 MHz, DMSO-*d*$_6$): δ (ppm) = 3.82 (s, 3H), 4.99 (s, 2H), 7.53 – 7.58 (m, 3H), 7.70 – 7.74 (m, 2H), 7.92 – 7.95 (m, 2H), 8.21 – 8.25 (m, 2H), 8.80 (s, 1H), 10.86 (s, 1H); ^{13}C NMR (100 MHz, DMSO-*d*$_6$): δ (ppm) = 51.96, 99.46, 118.62, 124.47, 126.88, 128.55, 128.84, 130.48, 131.20, 142.79, 155.52, 156.50, 159.33, 165.45, 165.74, 175.49; HRMS (ESI-) *m/z* calc. for $C_{21}H_{15}N_4O_5$ $[M\text{-}H]^-$ 403.1048, found 403.1048; HPLC purity 98.54 % at 254 nm (t_R = 8.647 min); Rf = 0.30 (EtOAc/n-Hex, 2:1, v/v).

*N-(4-nitrophenyl)-2-(4-oxo-3-phenylisoxazolo[5,4-d]pyrimidin-5(4H)-yl)acetamide (**40**):* Yield: 72 %; white solid; Mp 197 – 199 °C; ^{1}H NMR (400 MHz, DMSO-*d*$_6$): δ (ppm) = 5.01 (s, 2H), 7.53 – 7.59 (m, 3H), 7.81 – 7.85 (m, 2H), 8.21 – 8.27 (m, 4H), 8.81 (s, 1H), 11.13 (s, 1H); ^{13}C NMR (100 MHz, DMSO-*d*$_6$): δ (ppm) = 49.38, 99.45, 118.97, 125.17, 126.84, 128.53, 128.84, 131.21, 142.60, 144.49, 155.49, 156.47, 159.32, 165.86, 175.47; HRMS (ESI-) *m/z* calc. for $C_{19}H_{12}O_5N_5$ $[M\text{-}H]^-$ 390.0844, found 390.0846; HPLC purity 98.74 % at 254 nm (t_R = 8.983 min); Rf = 0.08 (EtOAc/n-Hex; 1:2, v/v).

*3-(4-bromophenyl)-5-(2-(3,4-dihydroquinolin-1(2H)-yl)-2-oxoethyl)isoxazolo[5,4-d]pyrimidin-4(5H)-one (**41**):* Yield: 53 %; white solid; Mp 184 – 186 °C; ^{1}H NMR (400 MHz, DMSO-*d*$_6$): δ (ppm) = 1.87 – 2.00 (m, 2H), 2.77 (t, *J* = 6.0 Hz, 2H), 3.71 – 3.82 (m, 2H), 5.12 (s, 2H), 7.03 – 7.36 (m, 3H), 7.45 – 7.65 (m, 1H), 7.77 – 7.81 (m, 2H), 8.15 – 8.22 (m, 2H), 8.72 (s, 1H); ^{13}C NMR (100 MHz, DMSO-*d*$_6$): δ (ppm) = 23.33, 26.22, 43.35, 48.45, 99.42, 124.17, 124.99, 126.15, 126.24, 128.89, 130.53, 132.01, 132.58, 137.58, 155.64, 156.42, 158.51, 165.57, 175.55; HRMS (ESI+): *m/z* calc. for $C_{22}H_{18}BrN_4O_3$ $[M+H]^+$ 465.0557, found 465.0556; HPLC purity 98.99 % at 254 nm (t_R = 11.153 min); Rf = 0.29 (EtOAc/n-Hex, 1:1, v/v).

*5-(2-(3,4-dihydroquinolin-1(2H)-yl)-2-oxoethyl)-3-(4-methoxyphenyl)isoxazolo[5,4-d]pyrimidin-4(5H)-one (**42**):* Yield: 12 %; white solid; Mp 171 – 178 °C; ^{1}H NMR (400 MHz, DMSO-*d*$_6$): δ (ppm) = 1.89 – 1.98 (m, 2H), 2.77 (t, *J* = 6.3 Hz, 2H), 3.73 – 3.82 (m, 2H), 5.12 (s, 2H), 7.09 – 7.13 (m, 2H), 7.14 – 7.31 (m, 3H), 7.46 – 7.64 (m, 1H), 8.18 – 8.25 (m, 2H), 8.69 (s, 1H); ^{13}C NMR (100 MHz, DMSO-*d*$_6$): δ (ppm) = 23.32, 26.21, 43.74, 48.42, 55.40, 99.29, 114.29, 119.15, 124.16, 126.22, 128.86, 130.20, 137.58, 155.38, 156.51, 158.86, 161.48, 165.64, 175.36; HRMS (ESI-) *m/z* calc. for $C_{23}H_{19}N_4O_4$ $[M\text{-}H]^-$ 415.1412, found 415.1413; HPLC purity 92.29 % at 254 nm (t_R = 10.407 min); Rf = 0.45 (EtOAc/n-Hex; 2:1, v/v).

*5-(2-(3,4-dihydroquinolin-1(2H)-yl)-2-oxoethyl)-3-(4-(trifluoromethyl)phenyl)isoxazolo[5,4-d]pyrimidin-4(5H)-one (**43**):* Yield: 14 %; white solid; Mp 173 – 176 °C; ^{1}H NMR (400 MHz, DMSO-*d*$_6$): δ (ppm) = 1.88 – 2.00 (m, 2H), 2.77 (t, *J* = 6.3 Hz, 2H), 3.72 – 3.84 (m, 2H), 5.05 – 5.25 (s, 2H), 7.12 – 7.32 (m, 3H), 7.45 – 7.63 (m, 1H), 7.94 – 7.98 (m, 2H), 8.41 – 8.47 (m, 2H), 8.74 (s, 1H); ^{13}C NMR (100 MHz, DMSO-*d*$_6$): δ (ppm) = 23.31, 26.21, 43.63, 48.43, 99.54, 118.57, 123.93 (q, $J_{C\text{-}F}$ = 272.4 Hz), 124.15, 125.75, 125.86 (q, $J_{C\text{-}F}$ = 3.4 Hz), 128.87, 129.42, 130.86 (q, $J_{C\text{-}F}$ = 1.3 Hz), 131.06 (q, $J_{C\text{-}F}$ = 32.0 Hz), 137.46, 155.75, 156.33, 158.30, 161.86, 165.52, 175.61; HRMS (ESI-) *m/z* calc. for $C_{23}H_{16}F_3N_4O_3$ $[M\text{-}H]^-$ 453.1180, found 453.1180; HPLC purity 98.49 % at 254 nm (t_R = 11.877 min); Rf = 0.53 (EtOAc/n-Hex; 2:1, v/v).

*4-(5-(2-(3,4-dihydroquinolin-1(2H)-yl)-2-oxoethyl)-4-oxo-4,5-dihydroisoxazolo[5,4-d]pyrimidin-3-yl)benzonitrile (**44**):* Yield: 18 %; white solid; Mp 202 – 204 °C; ^{1}H NMR (400 MHz, DMSO-*d*$_6$): δ (ppm) = 1.89 – 2.00 (m, 2H), 2.77 (t, *J* = 6.4 Hz, 2H), 3.72 – 3.81 (m, 2H), 5.13 (s, 2H), 7.10 – 7.33 (m, 3H), 7.47 – 7.62 (m, 1H), 8.04 – 8.08 (m, 2H), 8.39 – 8.44 (m, 2H), 8.74 (s, 1H); ^{13}C NMR (100 MHz, DMSO-*d*$_6$): δ (ppm) = 23.31, 26.21, 44.07, 48.45, 99.56, 113.61, 118.35, 124.15, 126.15, 128.88, 129.31, 131.26, 132.88, 137.55, 155.78,

156.33, 158.17, 165.50, 175.65; HRMS (ESI+): *m/z* calc. for $C_{23}H_{18}N_5O_3$ $[M+H]^+$ 412.1404, found 412.1396; HPLC purity 99.68 % at 254 nm (t_R = 9.317 min); Rf = 0.60 (EtOAc/n-Hex, 2:1, v/v).

*3-(4-(1H-tetrazol-5-yl)phenyl)-5-(2-(3,4-dihydroquinolin-1(2H)-yl)-2-oxoethyl)isoxazolo[5,4-d]pyrimidin-4(5H)-one (**45**):* To a solution of **44** (1.0 equiv.) in H_2O (5 mL) NaN_3 (1.5 equiv.) and $ZnBr_2$ (1.0 equiv.) were added. The reaction mixture was stirred at reflux for 18 hours, then extraction with 4 M HCl and EtOAc was performed. Combined organic phases were dried over Na_2SO_4, the solvent was removed under reduced pressure. Crude product was resuspended in 0.2 M NaOH and stirred for 1 hour. The formed precipitate was collected by filtration under reduced pressure, acidified with HCl and purified with flash column chromatography with EtOAc/n-Hex as the eluent to yield **45**. Yield: 15 %; white solid; Mp 216 – 220 °C; ^{1}H NMR (400 MHz, DMSO-d_6): δ (ppm) = 1.88 – 1.98 (m, 2H), 2.76 (t, J = 6.5 Hz, 2H), 3.72 – 3.80 (m, 2H), 5.16 (s, 2H), 7.10 – 7.30 (m, 3H), 7.48 – 7.66 (m, 1H), 8.13 – 8.17 (m, 2H), 8.24 – 8.28 (m, 2H), 8.76 (d, J = 2.2 Hz, 1H), 1H is exchanged; ^{13}C NMR (100 MHz, DMSO-d_6): δ (ppm) = 23.39, 26.31, 43.46, 48.55, 99.53, 124.25, 125.44, 125.47, 126.04, 126.33, 128.86, 128.95, 134.91, 134.99, 137.64, 155.58, 156.56, 159.32, 160.25, 165.76, 175.54; HRMS (ESI+): *m/z* calc. for $C_{23}H_{19}N_8O_3$ $[M+H]^+$ 455.1575, found 455.1570; HPLC purity 95.98 % at 254 nm (t_R = 7.480 min); Rf = 0.05 (EtOAc/n-Hex, 2:1, v/v).

*3-(4-(benzyloxy)phenyl)-5-(2-(3,4-dihydroquinolin-1(2H)-yl)-2-oxoethyl)isoxazolo[5,4-d]pyrimidin-4(5H)-one (**46**):* Yield: 22 %; white solid; Mp 200 – 202 °C; ^{1}H NMR (400 MHz, DMSO-d_6): δ (ppm) = 1.90 – 1.99 (m, 2H), 2.77 (t, J = 6.4 Hz, 2H), 3.74 – 3.80 (m, 2H), 5.11 (s, 2H), 5.20 (s, 2H), 7.16 – 7.21 (m, 2H), 7.19 – 7.30 (m, 3H), 7.32 – 7.36 (m, 1H), 7.38 – 7.43 (m, 2H), 7.46 – 7.50 (m, 2H), 7.51 – 7.62 (m, 1H), 8.19 – 8.23 (m, 2H), 8.68 (s, 1H); ^{13}C NMR (100 MHz, DMSO-d_6): δ (ppm) = 23.31, 26.20, 43.40, 48.39, 69.41, 99.29, 115.10, 119.35, 124.15, 126.12, 127.61, 127.83, 128.00, 128.51, 128.59, 128.85, 130.20, 136.65, 137.58, 155.37, 156.50, 158.82, 160.56, 165.62, 175.36; HRMS (ESI+): *m/z* calc. for $C_{29}H_{25}N_4O_4$ $[M+H]^+$ 493.1870, found 493.1864; HPLC purity 95.04 % at 254 nm (t_R = 11.433 min); Rf = 0.60 (EtOAc/n-Hex, 2:1, v/v).

*3-(3,4-difluorophenyl)-5-(2-(3,4-dihydroquinolin-1(2H)-yl)-2-oxoethyl)isoxazolo[5,4-d]pyrimidin-4(5H)-one (**47**):* Yield: 48 %; white solid; Mp 187 – 189 °C; ^{1}H NMR (400 MHz, DMSO-d_6): δ (ppm) = 1.90 – 1.98 (m, 2H), 2.77 (t, J = 6.5 Hz, 2H), 3.72 – 3.82 (m, 2H), 5.14 (s, 2H), 7.10 – 7.33 (m, 3H), 7.49 – 7.62 (m, 1H), 7.67 (dt, J_1 = 8.6 Hz, J_2 = 10.6 Hz, 1H), 8.11 – 8.18 (m, 1H), 8.32 – 8.42 (m, 1H), 8.73 (s, 1H); ^{13}C NMR (100 MHz, DMSO-d_6): δ (ppm) = 23.30, 26.20, 43.43, 48.43, 99.37, 117.98 (d, J_{C-F} = 19.7 Hz), 118.40 (d, J_{C-F} = 17.7 Hz), 124.14, 124.33 (dd, J_{C-F} = 3.7 Hz, J_{C-F} = 7.2 Hz), 125.89 (dd, J_{C-F} = 3.5 Hz, J_{C-F} = 7.1 Hz), 126.13, 128.87, 137.55, 149.42 (dd, J_{C-F} = 13.0 Hz, J_{C-F} = 246.2 Hz), 151.21 (dd, J_{C-F} = 12.5 Hz, J_{C-F} = 251.4 Hz), 155.70, 156.47, 157.57, 165.50, 175.55; HRMS (ESI+): *m/z* calc. for $C_{22}H_{17}F_2N_4O_3$ $[M+H]^+$ 423.1263, found 423.1253; HPLC purity 100.00 % at 254 nm (t_R = 10.503 min); Rf = 0.38 (EtOAc/n-Hex, 1:1, v/v).

*2-(3-(3,4-difluorophenyl)-4-oxoisoxazolo[5,4-d]pyrimidin-5(4H)-yl)-N-(4-nitrophenyl)acetamide (**48**):* Yield: 18 %; white solid; Mp 199 – 202 °C; ^{1}H NMR (400 MHz, DMSO-d_6): δ (ppm) = 5.03 (s, 2H), 7.67 (dt, J_1 = 8.6 Hz, J_2 = 10.5 Hz, 1H), 7.81 – 7.85 (m, 2H), 8.14 – 8.18 (m, 1H), 8.24 – 8.27 (m, 2H), 8.39 (ddd, J_1 = 2.1 Hz, J_2 = 7.8 Hz, J_3 = 11.8 Hz, 1H), 8.83 (s, 1H), 11.13 (s, 1H); ^{13}C NMR (100 MHz, DMSO-d_6): δ (ppm) = 49.35, 99.42, 117.98 (d, J_{C-F} = 19.9 Hz), 118.40 (d, J_{C-F} = 17.7 Hz), 118.99, 124.29 (dd, J_{C-F} = 3.9 Hz, J_{C-F} = 7.0 Hz), 125.17, 125.89 (dd, J_{C-F} = 3.6 Hz, J_{C-F} = 7.2 Hz), 142.62, 144.46, 149.40 (dd, J_{C-F} = 12.8 Hz, J_{C-F} = 245.7 Hz), 151.20 (dd, J_{C-F} = 13.1 Hz, J_{C-F} = 250.4 Hz), 155.69, 156.56, 157.59, 165.75, 175.56; HRMS (ESI-): *m/z* calc. for $C_{19}H_{10}F_2N_5O_5$ $[M-H]^-$ 426.0656, found 426.0656; HPLC purity 99.36 % at 254 nm (t_R = 9.567 min); Rf = 0.29 (EtOAc/n-Hex, 1:1, v/v).

*2-(3-(3-bromo-4-fluorophenyl)-4-oxoisoxazolo[5,4-d]pyrimidin-5(4H)-yl)-N-(4-nitrophenyl)acetamide (**49**):* Yield: 78 %; white solid; Mp 226 – 228 °C; ^{1}H NMR (400 MHz, DMSO-d_6): δ (ppm) = 5.03 (s, 2H), 7.60 (t, J = 8.7 Hz, 1H), 7.81 – 7.85 (m, 2H), 8.23 – 8.28 (m, 2H), 8.29 (ddd, J_1 = 2.2 Hz, J_2 = 4.8 Hz, J_3 = 8.8 Hz, 1H), 8.70 (dd, J_1 = 2.2 Hz, J_2 = 6.7 Hz, 1H), 8.83 (s, 1H), 11.15 (s, 1H); ^{13}C NMR (100 MHz, DMSO-d_6): δ (ppm) = 49.36, 99.47, 108.62 (d, J_{C-F} = 21.5 Hz), 117.51 (d, J_{C-F} = 22.9 Hz), 119.03, 125.03 (d, J_{C-F} = 3.9 Hz), 125.19, 129.92 (d, J_{C-F} = 8.2 Hz), 133.94, 142.66, 144.49, 155.70, 156.61, 157.37, 159.93 (d, J_{C-F} = 250.2 Hz), 165.81, 175.55; HRMS (ESI+): *m/z* calc. for $C_{19}H_{12}FBrN_5O_5$ $[M+H]^+$ 488.0000, found 487.9996; HPLC purity 97.13 % at 254 nm (t_R = 10.177 min); Rf = 0.57 (EtOAc/n-Hex, 2:1, v/v).

*5-(2-(3,4-dihydroquinolin-1(2H)-yl)-2-oxoethyl)-3-(thiophen-2-yl)isoxazolo[5,4-d]pyrimidin-4(5H)-one (**50**):* Yield: 38 %; white solid; Mp 202 – 205 °C; ^{1}H NMR (400 MHz, DMSO-d_6): δ (ppm) = 1.89 – 2.02 (m, 2H), 2.78 (t, J = 6.4 Hz, 2H), 3.74 – 3.83 (m, 2H), 5.13 (s, 2H), 7.07 – 7.30 (m, 3H), 7.28 (dd, J_1 = 3.7 Hz, J_2 = 5.0 Hz, 1H), 7.45 – 7.67 (m, 1H), 7.87 (dd, J_1 = 1.1 Hz, J_2 = 5.0 Hz, 1H), 8.47 – 8.54 (m, 1H), 8.71 (s, 1H); ^{13}C

NMR (100 MHz, DMSO-d_6): δ (ppm) = 23.32, 26.21, 43.83, 48.45, 98.88, 124.17, 125.71, 127.81, 128.30, 128.88, 128.97, 130.76, 133.41, 137.59, 144.08, 154.36, 155.62, 156.28, 165.57, 175.30; HRMS (ESI-) *m/z* calc. for $C_{20}H_{15}N_4O_3S$ $[M-H]^-$ 391.0870, found 391.0872; HPLC purity 95.33 % at 254 nm (t_R = 9.647 min); Rf = 0.45 (EtOAc/n-Hex; 2:1, v/v).

*N-(4-nitrophenyl)-2-(4-oxo-3-(thiophen-2-yl)isoxazolo[5,4-d]pyrimidin-5(4H)-yl)acetamide (**51**):* Yield: 15 %; pale yellow solid; Mp 238l – 240 °C; ^{1}H NMR (400 MHz, DMSO-d_6): δ (ppm) = 5.03 (s, 2H), 7.26 (dd, J_1 = 3.8 Hz, J_2 = 5.0 Hz, 1H), 7.81 – 7.85 (m, 2H), 7.86 (dd, J_1 = 1.2 Hz, J_2 = 5.0 Hz, 1H), 8.23 – 8.28 (m, 2H), 8.51 (dd, J_1 = 1.2 Hz, J_2 = 3.8 Hz, 1H), 8.80 (s, 1H), 11.15 (s, 1H); ^{13}C NMR (100 MHz, DMSO-d_6): δ (ppm) = 49.34, 98.92, 118.99, 125.17, 127.75, 128.25, 130.74, 133.40, 142.61, 144.47, 154.35, 155.60, 156.35, 165.81, 175.30; HRMS (ESI-) *m/z* calc. for n$C_{17}H_{10}O_5N_5S$ $[M-H]^-$ 396.0408, found 396.0405; HPLC purity 91.46 % at 254 nm (t_R = 8.867 min); Rf = 0.08 (EtOAc/n-Hex, 1:2, v/v).

*5-(2-(3,4-dihydroquinolin-1(2H)-yl)-2-oxoethyl)-3-(thiophen-3-yl)isoxazolo[5,4-d]pyrimidin-4(5H)-one (**52**):* Yield: 14 %; slightly yellow solid; Mp 182 – 186 °C; ^{1}H NMR (400 MHz, DMSO-d_6): δ (ppm) = 1.91 – 1.99 (m, 2H), 2.78 (t, J = 6.5 Hz, 2H), 3.74 – 3.81 (m, 2H), 5.13 (s, 2H), 7.10 – 7.31 (m, 3H), 7.56 (s, 1H), 7.75 – 7.78 (m, 2H), 8.70 (s, 1H), 8.89 (s, 1H); ^{13}C NMR (100 MHz, DMSO-d_6): δ (ppm) = 23.29, 26.18, 43.33, 48.40, 99.20, 124.13, 125.83, 126.14, 127.61, 128.08, 128.84, 130.59, 137.57, 138.90, 144.36, 154.75, 155.45, 156.51, 165.56, 175.19; HRMS (ESI+) *m*/z calc. for $C_{20}H_{17}N_4O_3S$ $[M+H]^+$ 393.1027, found 393.1013; HPLC purity 95.13 % at 254 nm (t_R = 9.773 min); Rf = 0.61 (EtOAc/n-Hex, 2:1, v/v).

*N-(4-nitrophenyl)-2-(4-oxo-3-(thiophen-3-yl)isoxazolo[5,4-d]pyrimidin-5(4H)-yl)acetamide (**53**):* Yield: 21 %; slightly yellow solid; Mp 231 – 233 °C; ^{1}H NMR (400 MHz, DMSO-d_6): δ (ppm) = 5.03 (s, 2H), 7.75 – 7.78 (m, 2H), 7.81 – 7.85 (m, 2H), 8.23 – 8.27 (m, 2H), 8.80 (s, 1H), 8.90 (dd, J_1 = 1.5 Hz, J_2 = 2.5 Hz, 1H), 11.15 (s, 1H); ^{13}C NMR (100 MHz, DMSO-d_6): δ (ppm) = 49.42, 99.31, 119.00, 125.22, 125.83, 127.59, 128.16, 130.73, 142.63, 144.51, 154.80, 155.50, 156.65, 165.88, 175.25; HRMS (ESI+) *m*/z calc. for $C_{17}H_{10}N_5O_5S$ $[M-H]^-$ 396.0408, found 396.0410; HPLC purity 96.54 % at 254 nm (t_R = 8.770 min); Rf = 0.39 (EtOAc/n-Hex, 2:1, v/v).

2. Representative ^{1}H NMR, ^{13}C NMR and HPLC spectra

2.1 Compound 20

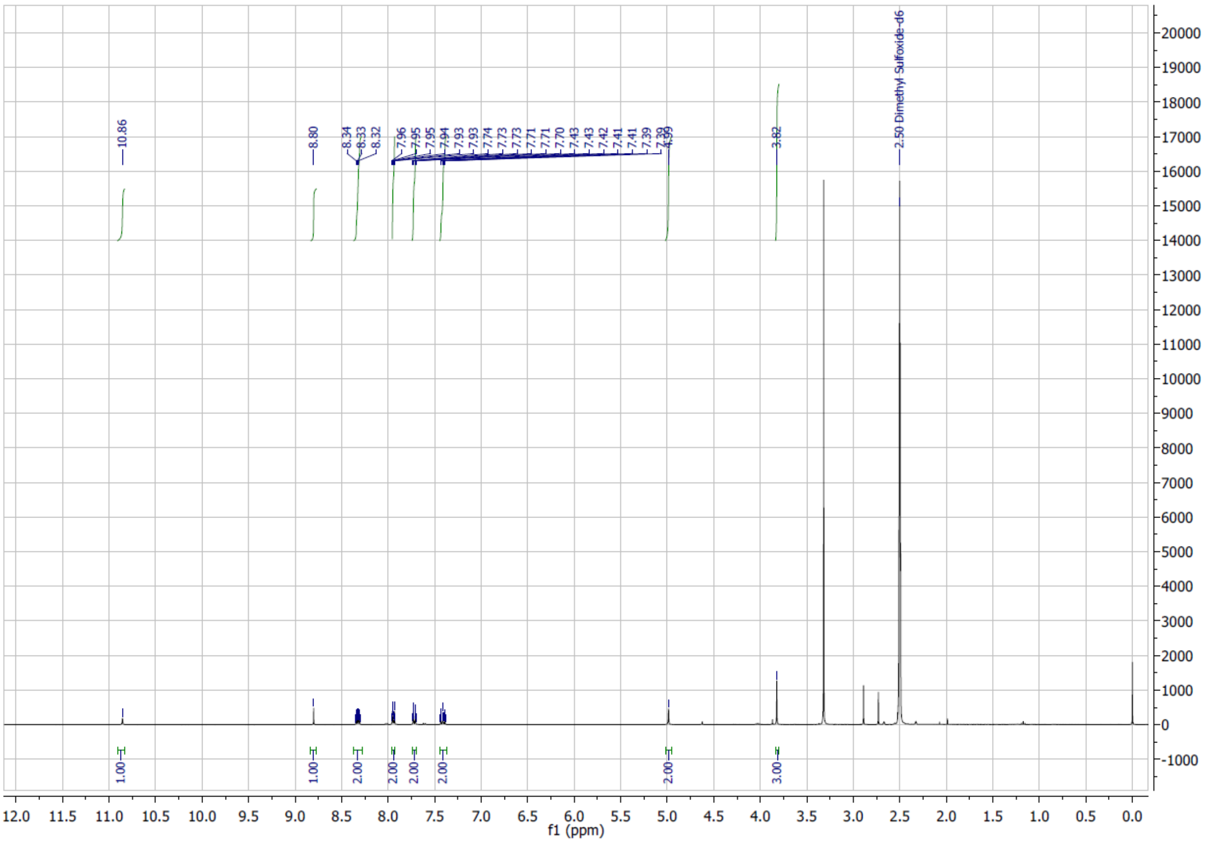

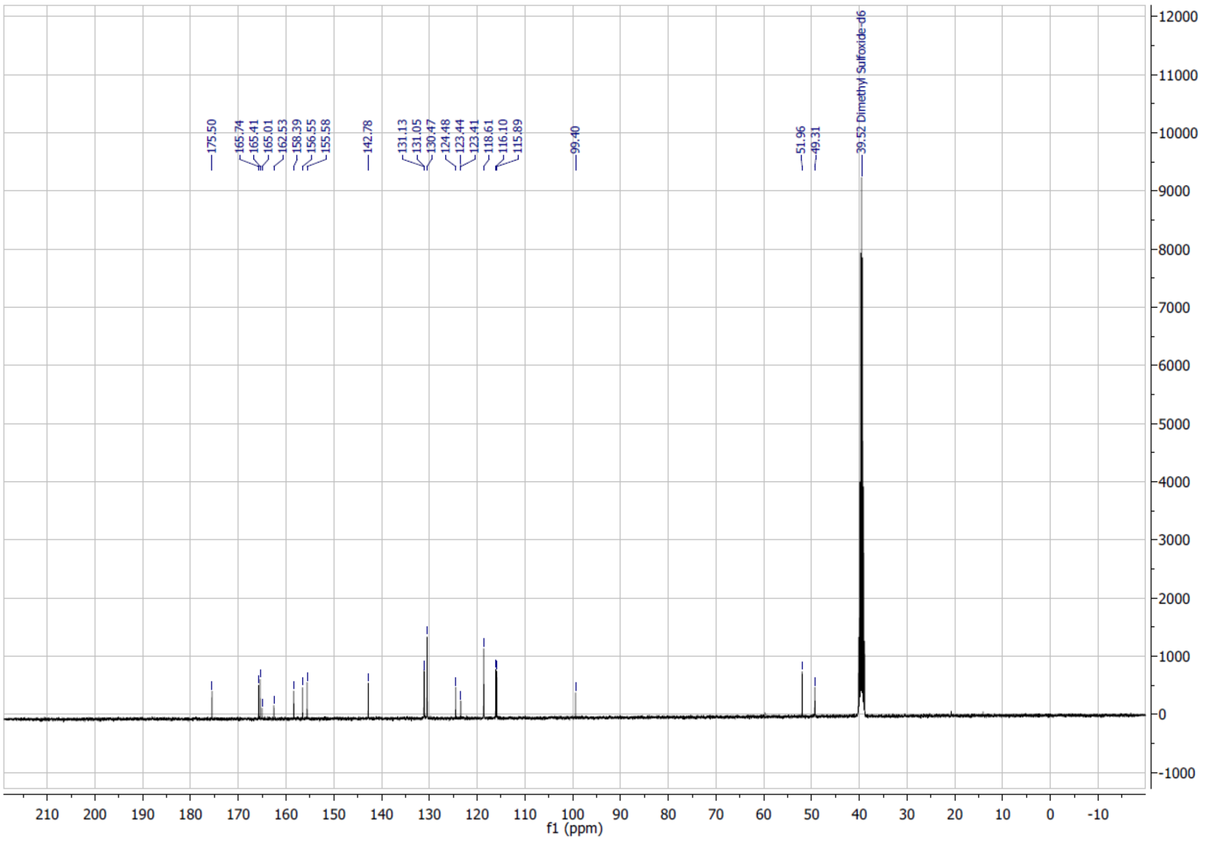

Chromatogram and Results

Injection Details

Injection Name:		*Run Time (min):*	**15,00**
Vial Number:	**GB1**	*Injection Volume:*	**5,00**
Injection Type:	**Unknown**	*Channel:*	**UV_VIS_2**
Calibration Level:		*Wavelength:*	**254**
Instrument Method:		*Bandwidth:*	**16**
Processing Method:	**Basic Quantitative**	*Dilution Factor:*	**1,0000**
Injection Date/Time:	**29.jul..20 11:36**	*Sample Weight:*	**1,0000**

Chromatogram

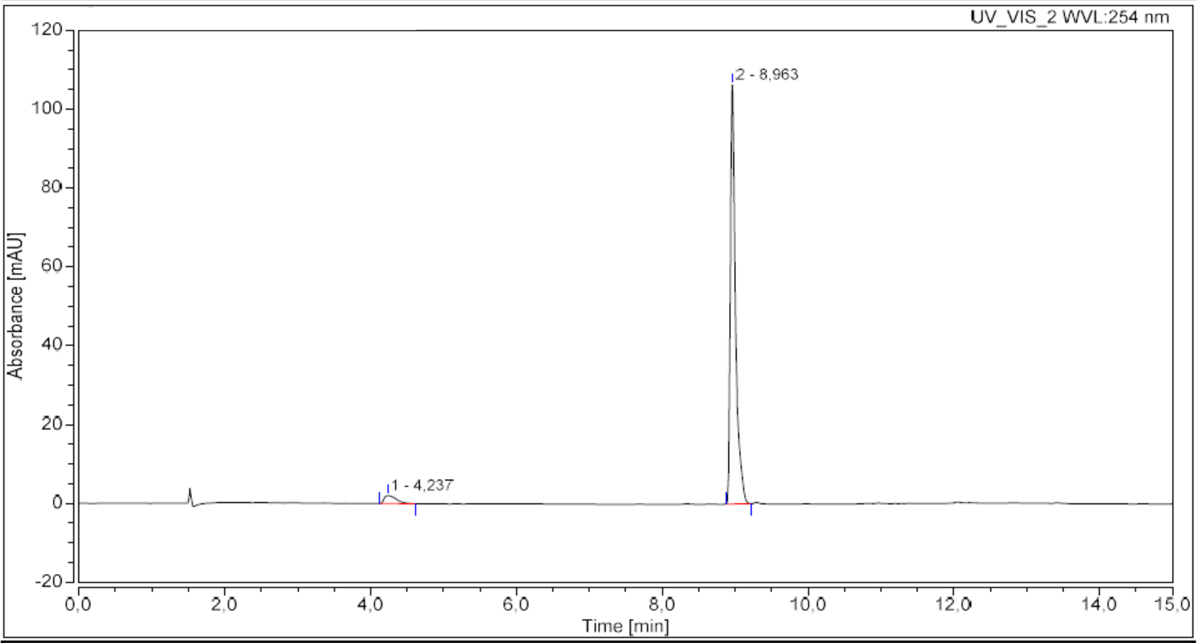

Integration Results

No.	Peak Name	Retention Time min	Area mAU*min	Height mAU	Relative Area %	Relative Height %	Amount n.a.
1		4,237	0,391	2,025	4,27	1,87	n.a.
2		8,963	8,776	106,341	95,73	98,13	n.a.
**Total:**			**9,167**	**108,366**	**100,00**	**100,00**	

2.2 *Compound 23*

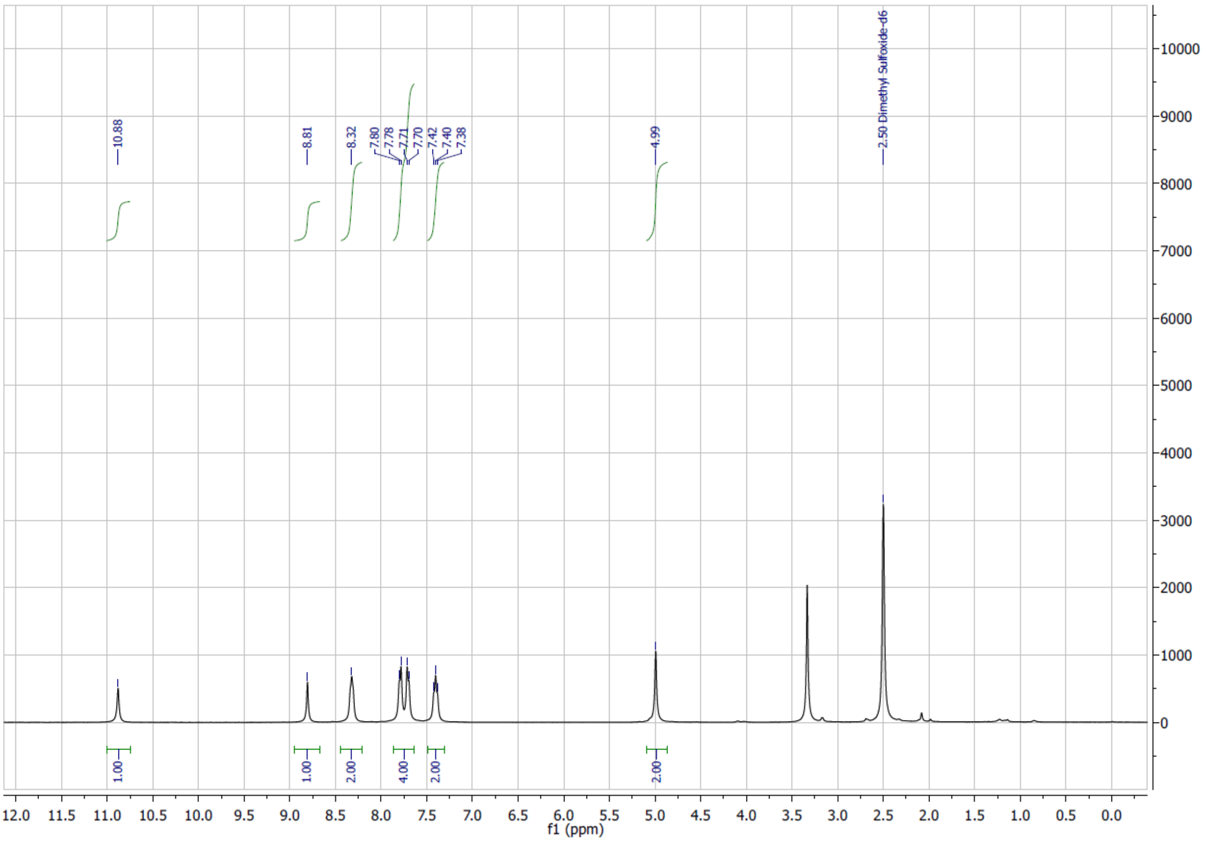

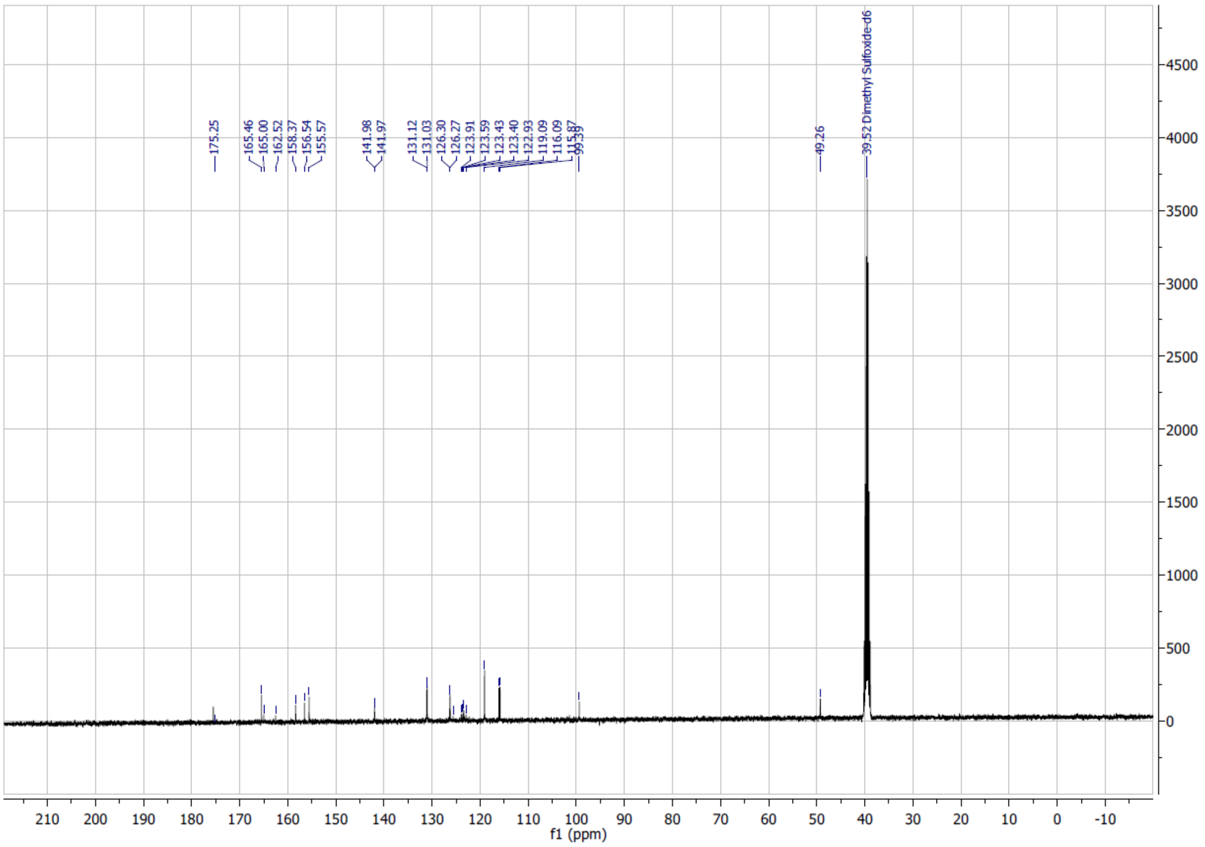

Chromatogram and Results

Injection Details

Injection Name:		*Run Time (min):*	**15,00**
Vial Number:	**BA5**	*Injection Volume:*	**5,00**
Injection Type:	**Unknown**	*Channel:*	**UV_VIS_2**
Calibration Level:		*Wavelength:*	**254**
Instrument Method:		*Bandwidth:*	**16**
Processing Method:	**Basic Quantitative**	*Dilution Factor:*	**1,0000**
Injection Date/Time:	**24.jun..20 14:51**	*Sample Weight:*	**1,0000**

Chromatogram

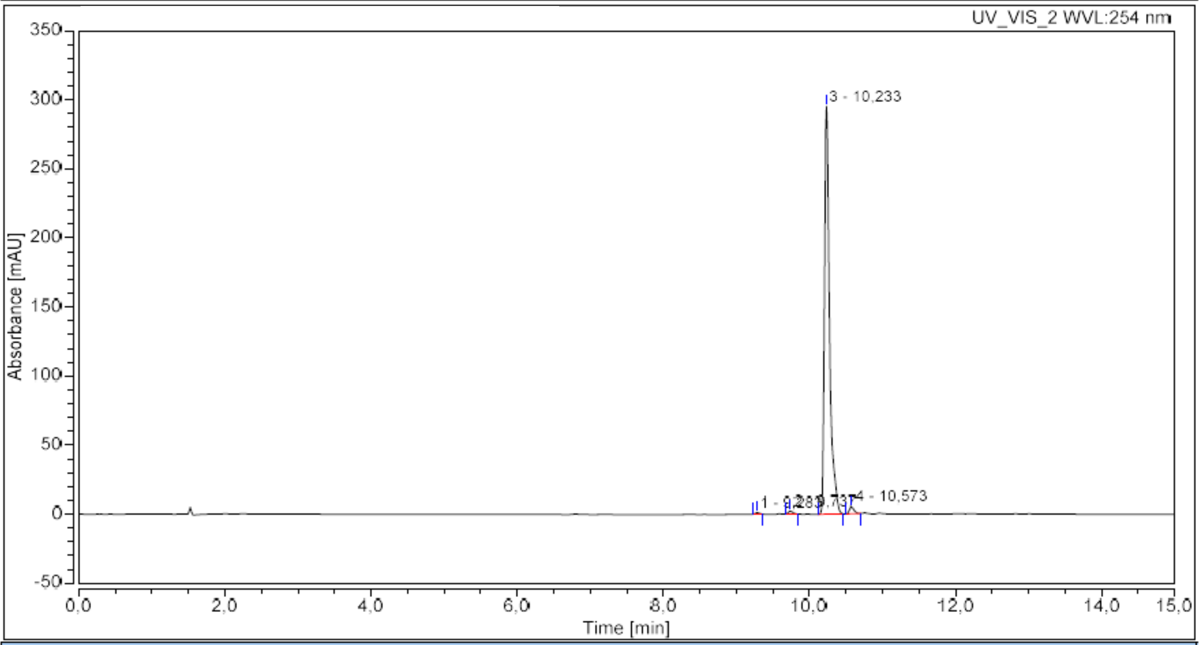

Integration Results

No.	Peak Name	Retention Time min	Area mAU*min	Height mAU	Relative Area %	Relative Height %	Amount n.a.
1		9,283	0,071	1,097	0,29	0,36	n.a.
2		9,737	0,145	2,054	0,60	0,68	n.a.
3		10,233	23,606	295,210	97,58	97,30	n.a.
4		10,573	0,368	5,056	1,52	1,67	n.a.
**Total:**			**24,190**	**303,417**	**100,00**	**100,00**	

2.3 *Compound 32*

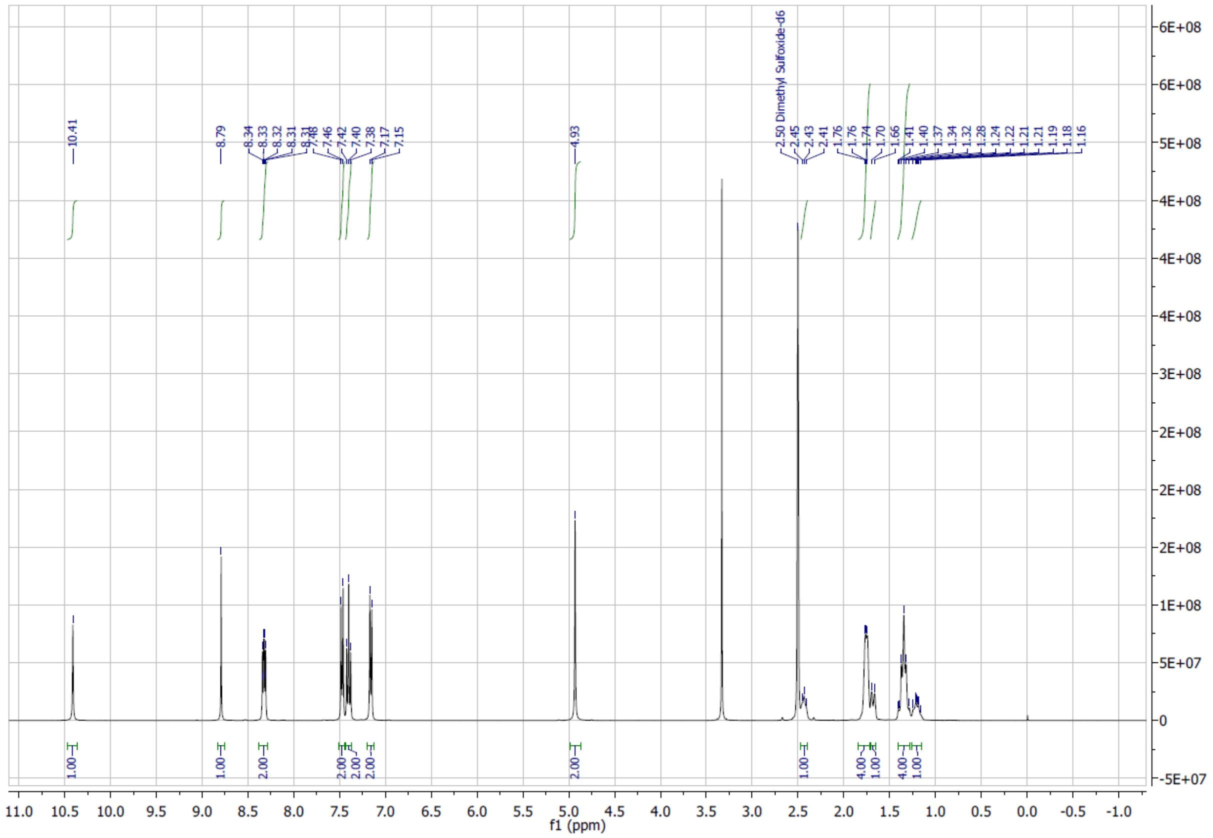

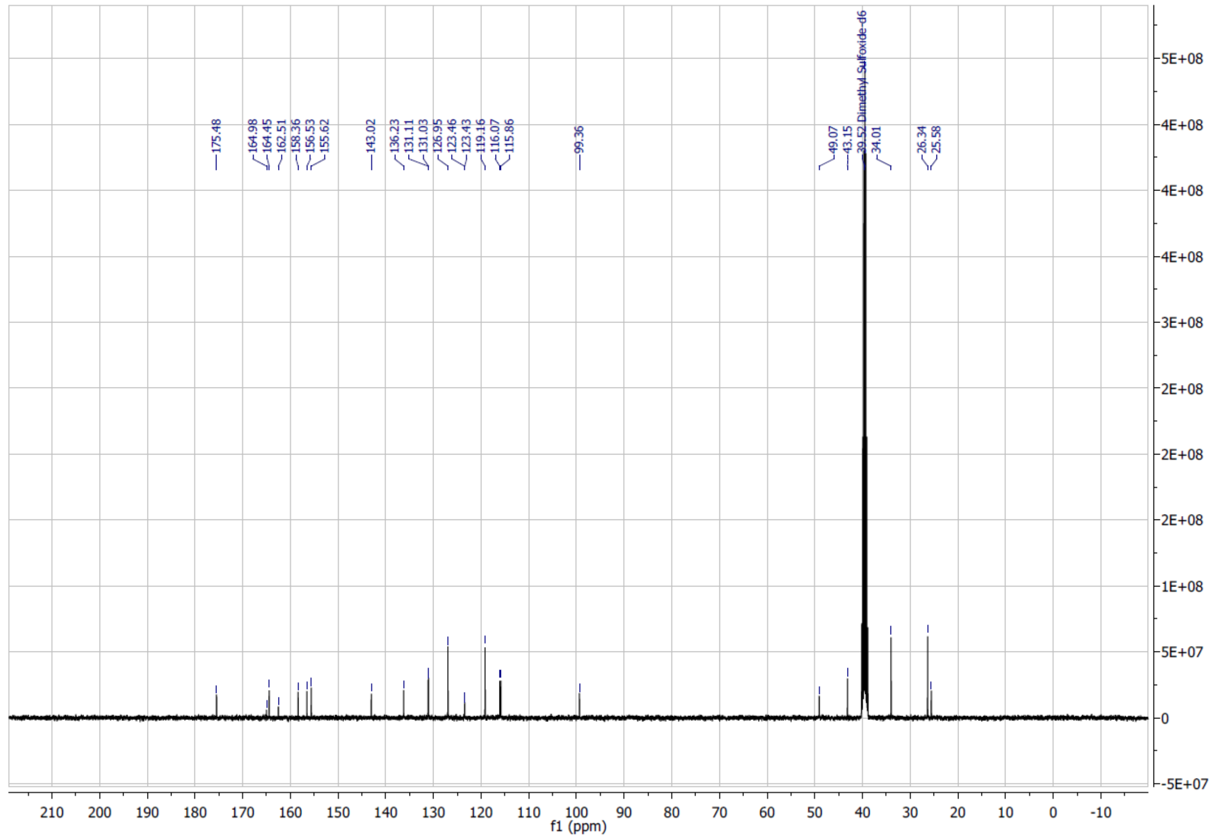

Chromatogram and Results

Injection Details

Injection Name:		*Run Time (min):*	**15,00**
Vial Number:	**BA7**	*Injection Volume:*	**5,00**
Injection Type:	**Unknown**	*Channel:*	**UV_VIS_2**
Calibration Level:		*Wavelength:*	**254**
Instrument Method:		*Bandwidth:*	**16**
Processing Method:	**Basic Quantitative**	*Dilution Factor:*	**1,0000**
Injection Date/Time:	**24.jun..20 15:29**	*Sample Weight:*	**1,0000**

Chromatogram

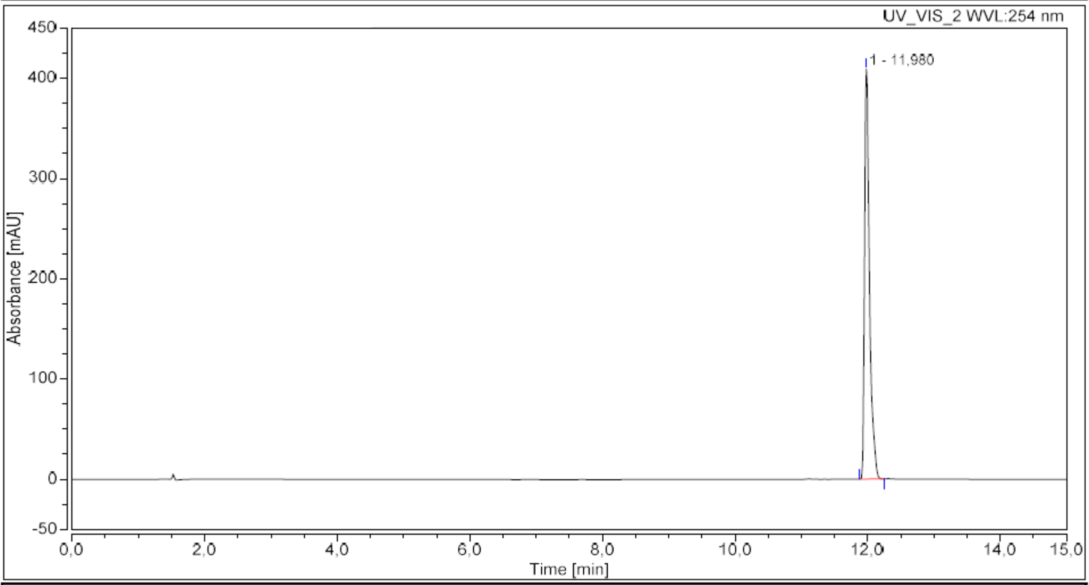

Integration Results

No.	Peak Name	Retention Time min	Area mAU*min	Height mAU	Relative Area %	Relative Height %	Amount n.a.
1		11,980	34,824	408,997	100,00	100,00	n.a.
**Total:**			**34,824**	**408,997**	**100,00**	**100,00**	

2.4 *Compound 39*

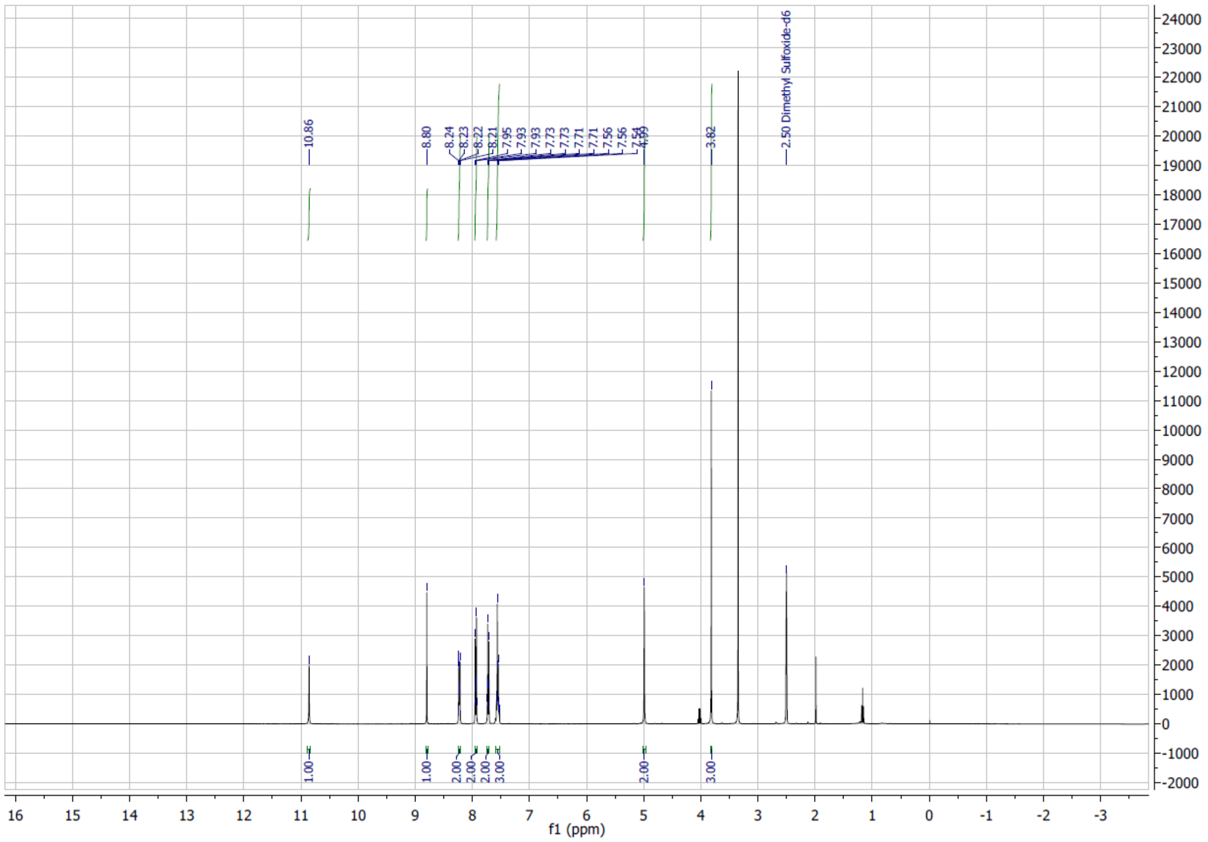

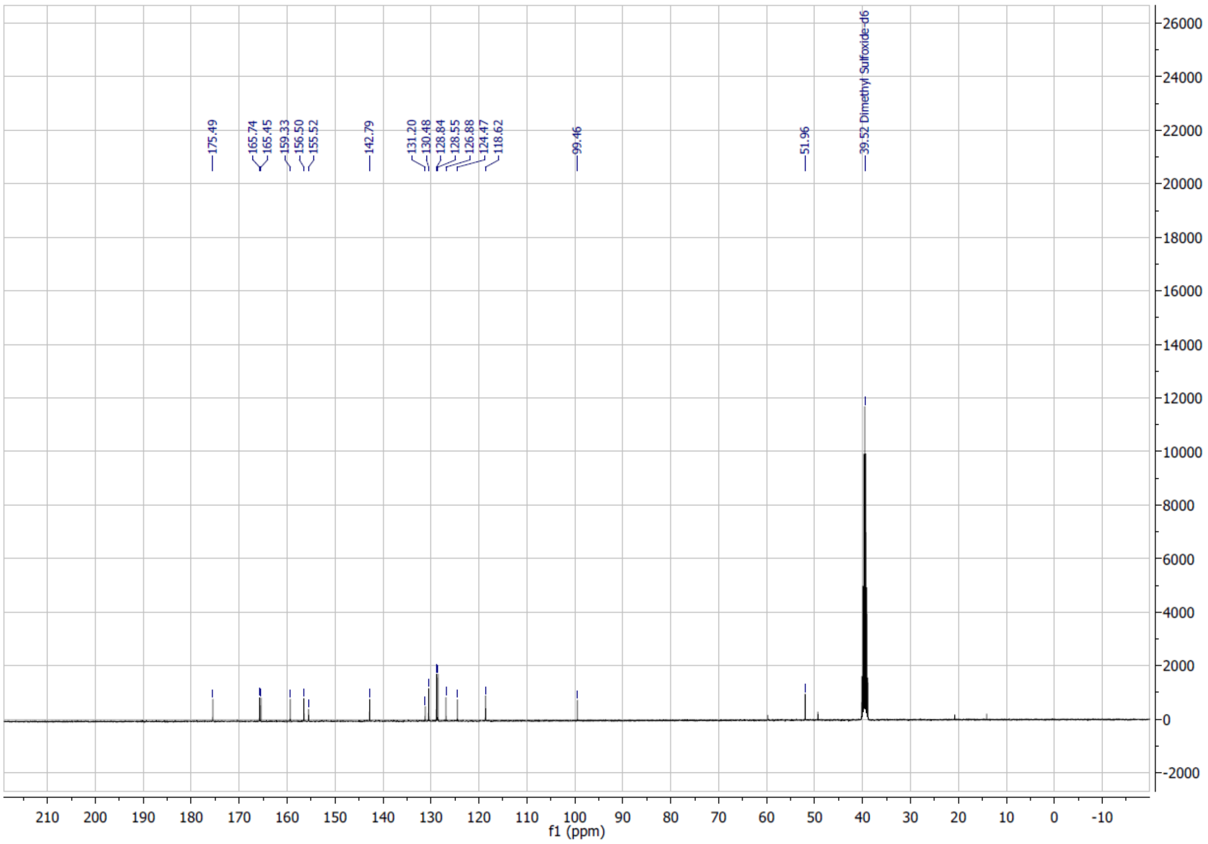

Chromatogram and Results

Injection Details

Injection Name:		*Run Time (min):*	**15,00**
Vial Number:	**GA5**	*Injection Volume:*	**5,00**
Injection Type:	**Unknown**	*Channel:*	**UV_VIS_2**
Calibration Level:		*Wavelength:*	**254**
Instrument Method:		*Bandwidth:*	**16**
Processing Method:	**Basic Quantitative**	*Dilution Factor:*	**1,0000**
Injection Date/Time:	**29.jul..20 10:20**	*Sample Weight:*	**1,0000**

Chromatogram

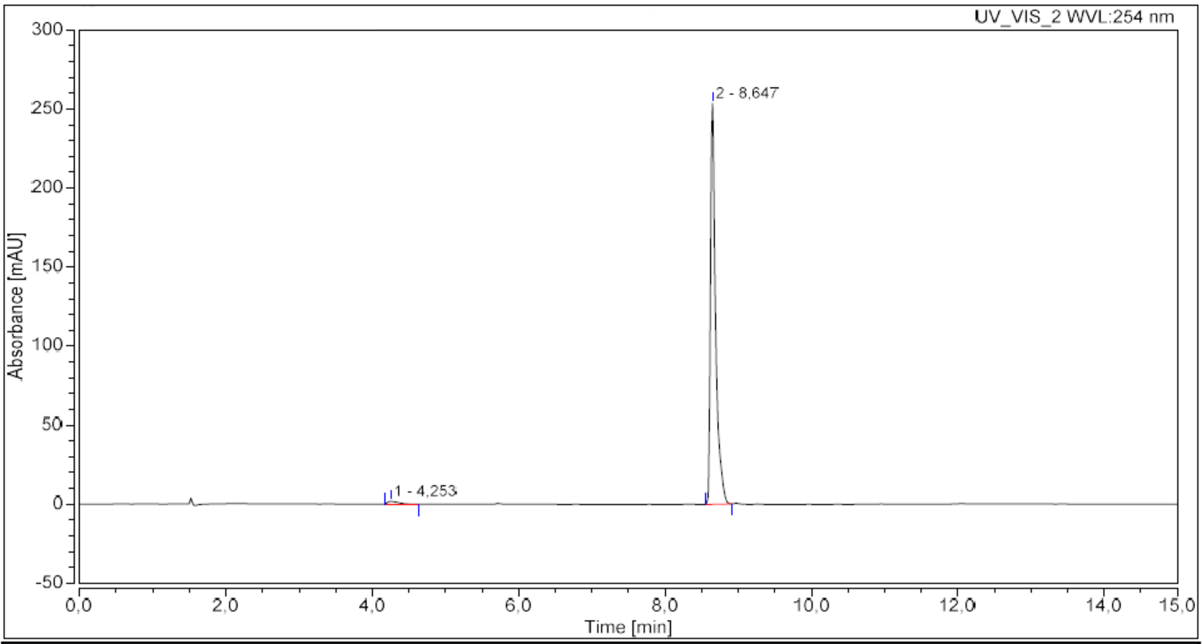

Integration Results

No.	Peak Name	Retention Time min	Area mAU*min	Height mAU	Relative Area %	Relative Height %	Amount
1		4,253	0,309	1,765	1,46	0,69	n.a.
2		8,647	20,821	253,721	98,54	99,31	n.a.
**Total:**			**21,131**	**255,486**	**100,00**	**100,00**	

2.5 Compound 53

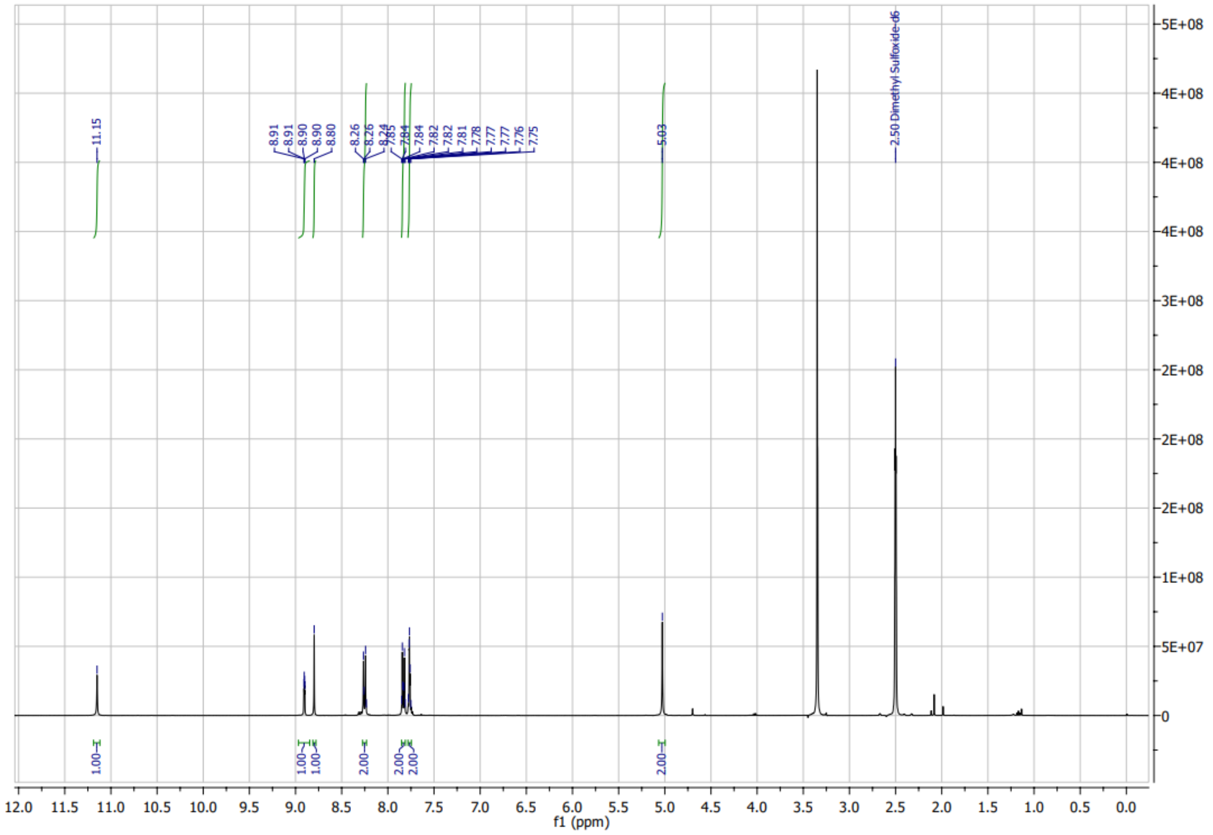

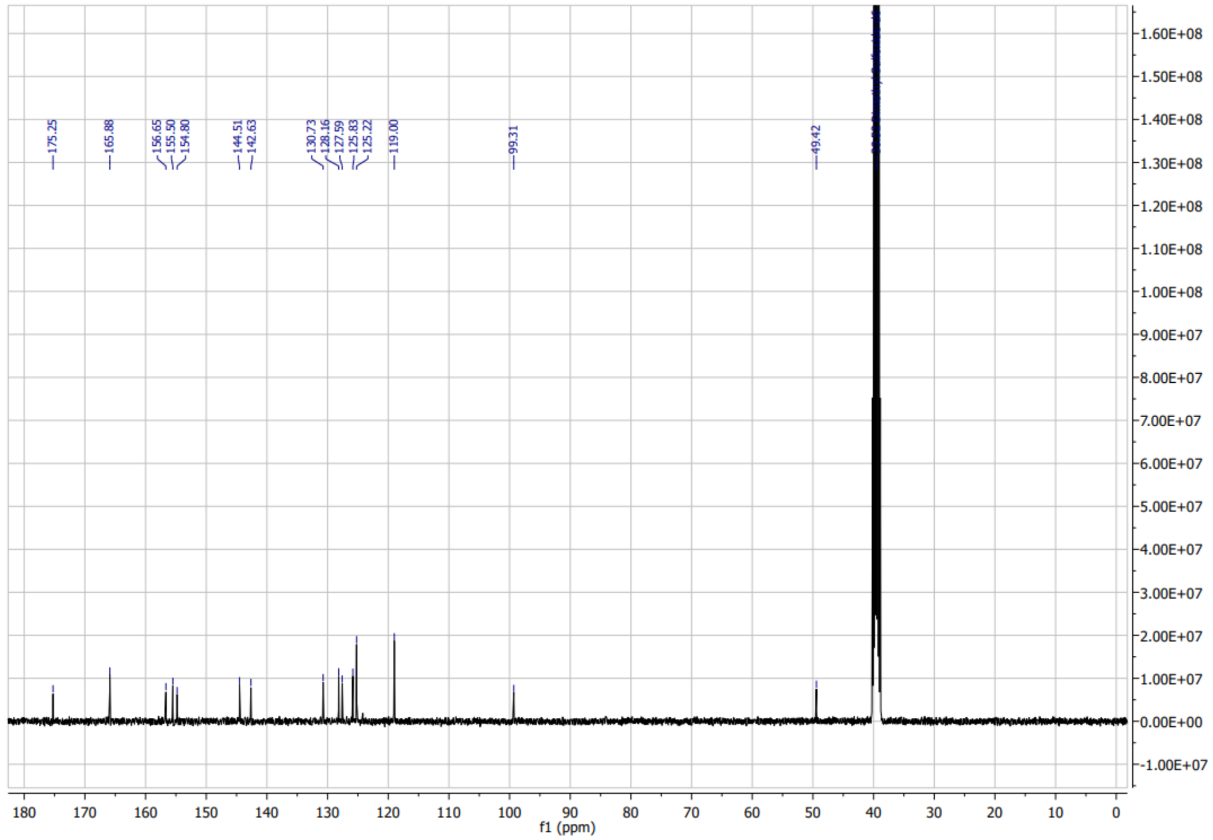

Chromatogram and Results

Injection Details

Injection Name:		*Run Time (min):*	**15,00**
Vial Number:	**RE3**	*Injection Volume:*	**5,00**
Injection Type:	**Unknown**	*Channel:*	**UV_VIS_2**
Calibration Level:		*Wavelength:*	**254**
Instrument Method:		*Bandwidth:*	**16**
Processing Method:	**Basic Quantitative**	*Dilution Factor:*	**1,0000**
Injection Date/Time:	**07.nov..19 21:01**	*Sample Weight:*	**1,0000**

Chromatogram

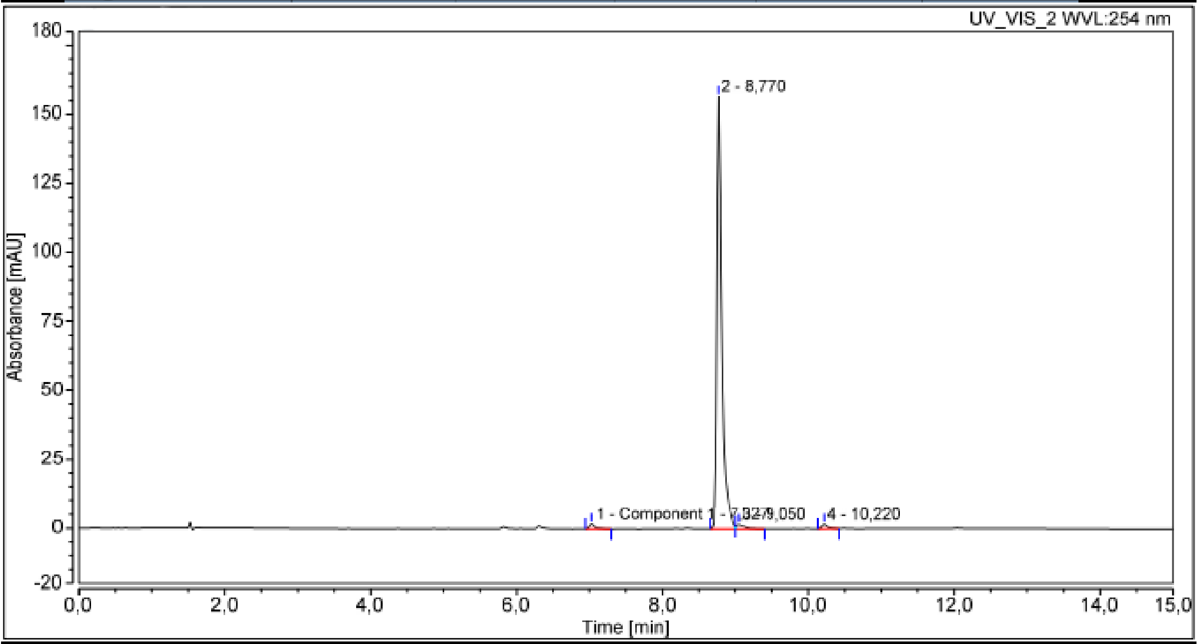

Integration Results

No.	Peak Name	Retention Time min	Area mAU*min	Height mAU	Relative Area %	Relative Height %	Amount
n.a.	Component 4	n.a.	n.a.	n.a.	n.a.	n.a.	n.a.
n.a.	Component 2	n.a.	n.a.	n.a.	n.a.	n.a.	n.a.
n.a.	Component 3	n.a.	n.a.	n.a.	n.a.	n.a.	n.a.
n.a.	Component 5	n.a.	n.a.	n.a.	n.a.	n.a.	n.a.
n.a.	Component 6	n.a.	n.a.	n.a.	n.a.	n.a.	n.a.
n.a.	Component 7	n.a.	n.a.	n.a.	n.a.	n.a.	n.a.
1	Component 1	7,027	0,148	1,748	1,11	1,08	n.a.
n.a.	Component 8	n.a.	n.a.	n.a.	n.a.	n.a.	n.a.
2		8,770	12,883	156,723	96,54	96,99	n.a.
3		9,050	0,177	1,533	1,32	0,95	n.a.
n.a.	Component 9	n.a.	n.a.	n.a.	n.a.	n.a.	n.a.
4		10,220	0,138	1,579	1,04	0,98	n.a.
**Total:**			**13,346**	**161,583**	**100,00**	**100,00**	

3. Determination of inhibitory potencies

3.1 Positive control

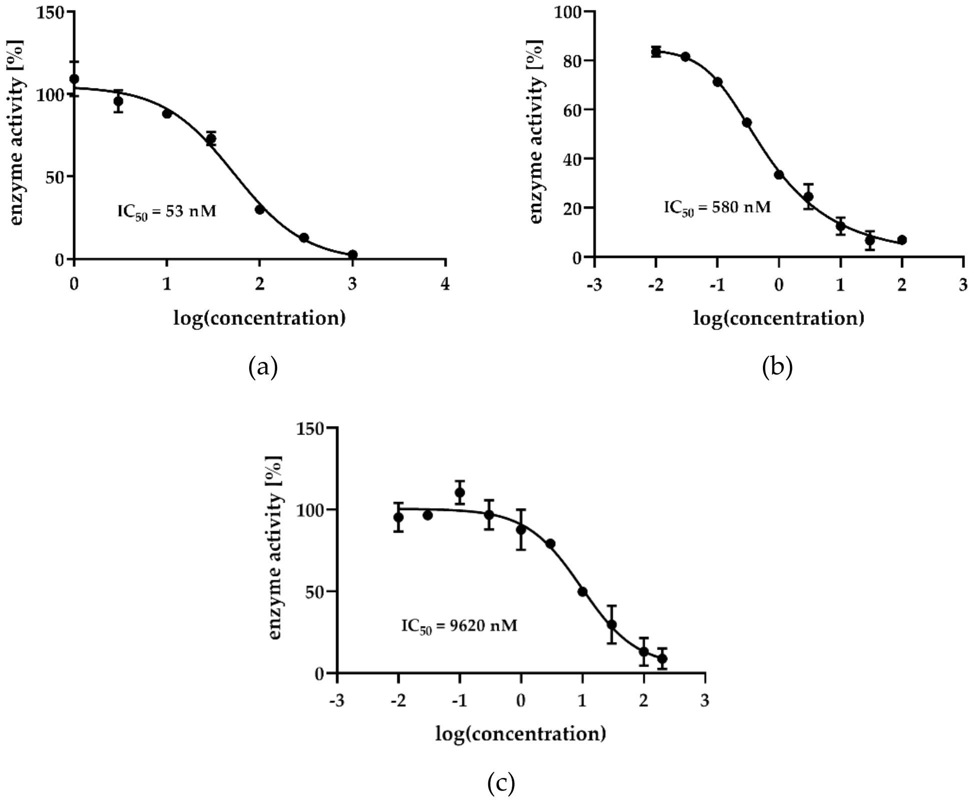

Figure S1. Inhibitory potencies of epacadostat (1) on (a) hIDO1, (b) hIDO2 and (c) hTDO. The percentage of residual activities of enzyme, measured at different concentrations of inhibitor, was plotted against logarithmic values of concentrations, and the IC_{50} value was determined.

3.2 *Inhibitory potencies for the most potent compounds (graphs for IC_{50} calculations)*

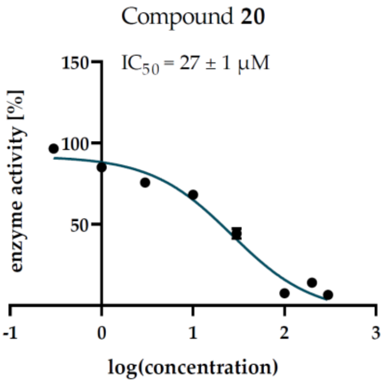

(a)

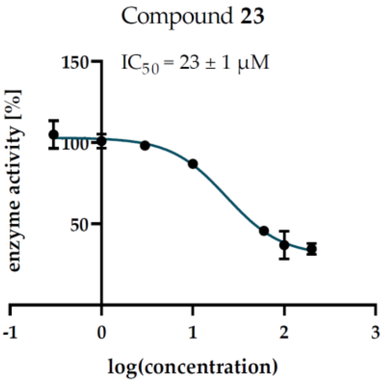

(b)

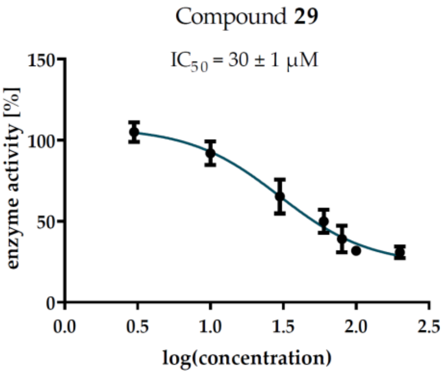

(c)

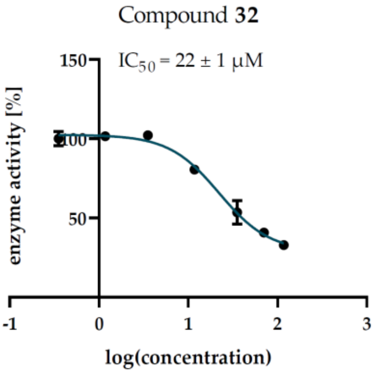

(d)

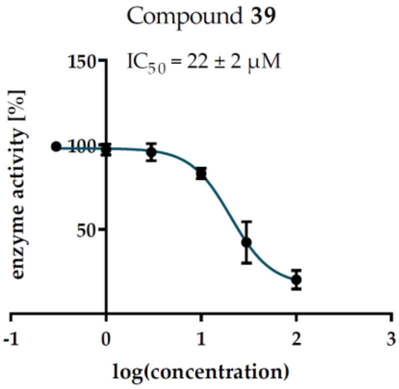

(e)

Figure S2. Inhibitory potencies of most potent compounds 20 (a), 23 (b), 29 (c), 32 (d) and 39 (e).

References

1. Pícha, J.; Cibulka, R.; Hampl, F.; Liška, F.; Pařík, P.; Pytela, O. Reactivity of *p*-substituted benzaldoximes in the cleavage of *p*-nitrophenyl acetate: Kinetics and mechanism. *Collect Czech Chem Commun* **2004**, *69*, 397–413, doi:10.1135/cccc20040397.
2. Lin, C.-F.; Yang, J.-S.; Chang, C.-Y.; Kuo, S.-C.; Lee, M.-R.; Huang, L.-J. Synthesis and anticancer activity of benzyloxybenzaldehyde derivatives against HL-60 cells. *Bioorg Med Chem* **2005**, *13*, 1537–1544, doi:10.1016/j.bmc.2004.12.026.
3. Mokhtari, J.; Naimi-Jamal, M.R.; Hamzeali, H.; Dekamin, M.G.; Kaupp, G. Kneading ball-milling and stoichiometric melts for the quantitative derivatization of carbonyl compounds with gas–solid recovery. *ChemSusChem* **2009**, *2*, 248–254, doi:10.1002/cssc.200800258.
4. Campaigne, E.; Thomas, H.L. 3-Substituted thiophenes. IX. [1] 3-Thienones and 5-(3-thienyl)-hydantoins [2]. *J Am Chem Soc* **1955**, *77*, 5365–5369, doi:10.1021/ja01625a048.
5. Yu, G.J.; Yang, B.; Verkman, A.S.; Kurth, M.J. Isoxazolopyrimidines as novel ΔF508-CFTR correctors. *Synlett* **2010**, *2010*, 1063–1066, doi:10.1055/s-0029-1219781.
6. Rajagopalan, P.; Talaty, C.N. Synthesis of 4-hydroxy-, 4-chloro-, 4-amino- and 4-substituted aminoisoxazolo [5.4-*d*] pyrimidines. *Tetrahedron* **1967**, *23*, 3541–3543, doi:10.1016/S0040-4020(01)92315-4.
7. Alyabiev, S.B.; Kravchenko, D.V.; Ivachtchenko, A.V. Regioselective synthesis of substituted isoxazolo[5,4-*d*]pyrimidines. *Mendeleev Commun* **2008**, *18*, 144–146, doi:10.1016/j.mencom.2008.05.011.
8. Roberti, M.; Schipani, F.; Bagnolini, G.; Milano, D.; Giacomini, E.; Falchi, F.; Balboni, A.; Manerba, M.; Farabegoli, F.; De Franco, F.; et al. Rad51/BRCA2 disruptors inhibit homologous recombination and synergize with olaparib in pancreatic cancer cells. *Eur J Med Chem* **2019**, *165*, 80–92, doi:10.1016/j.ejmech.2019.01.008.
9. Posevins, D.; Suta, K.; Turks, M. Indium-triflate-catalyzed Ritter reaction in liquid sulfur dioxide. *Eur J Org Chem* **2016**, *2016*, 1414–1419, doi:10.1002/ejoc.201600013.
10. Ma, L.; Xie, C.; Ma, Y.; Liu, J.; Xiang, M.; Ye, X.; Zheng, H.; Chen, Z.; Xu, Q.; Chen, T.; et al. Synthesis and biological evaluation of novel 5-benzylidenethiazolidine-2,4-dione derivatives for the treatment of inflammatory diseases. *J Med Chem* **2011**, *54*, 2060–2068, doi:10.1021/jm1011534.
11. Meng, J.; Yu, S.; Wan, S.; Ren, S.; Jiang, T. Synthesis, saccharide-binding and anti-cancer cell proliferation properties of arylboronic acid aerivatives of andoquinolines. *Chem Biol Drug Des* **2011**, *78*, 816–825, doi:10.1111/j.1747-0285.2011.01196.x.
12. Komatsu, T.; Furubayashi, Y.; Nishide, H.; Tsuchida, E. 5,10,15,20-Tetrakis($\alpha,\alpha,\alpha,\alpha$-o-pivalamidophenyl)porphinatoiron(II) bearing a covalently linked axial imidazole via m-aminobenzoic acid. Synthesis and influence of imidazole basicity on O2-binding affinity. *Inorg Chim Acta* **1999**, *295*, 234–238, doi:10.1016/S0020-1693(99)00346-1.
13. Henderson, B.J.; Carper, D.J.; González-Cestari, T.F.; Yi, B.; Mahasenan, K.; Pavlovicz, R.E.; Dalefield, M.L.; Coleman, R.S.; Li, C.; McKay, D.B. Structure–activity relationship studies of sulfonylpiperazine analogues as novel negative allosteric modulators of human neuronal nicotinic receptors. *J Med Chem* **2011**, *54*, 8681–8692, doi:10.1021/jm201294r.
14. Suchý, M.; Milne, M.; Elmehriki, A.A.H.; McVicar, N.; Li, A.X.; Bartha, R.; Hudson, R.H.E. Introduction of peripheral carboxylates to decrease the charge on Tm3+ DOTAM-alkyl complexes: Implications for detection sensitivity and in vivo toxicity of PARACEST MRI contrast agents. *J Med Chem* **2015**, *58*, 6516–6532, doi:10.1021/acs.jmedchem.5b00621.
15. Flipo, M.; Willand, N.; Lecat-Guillet, N.; Hounsou, C.; Desroses, M.; Leroux, F.; Lens, Z.; Villeret, V.; Wohlkönig, A.; Wintjens, R.; et al. Discovery of novel *N*-phenylphenoxyacetamide derivatives as EthR inhibitors and ethionamide boosters by combining high-throughput screening and synthesis. *J Med Chem* **2012**, *55*, 6391–6402, doi:10.1021/jm300377g.
16. Drewe, W.C.; Nanjunda, R.; Gunaratnam, M.; Beltran, M.; Parkinson, G.N.; Reszka, A.P.; Wilson, W.D.; Neidle, S. Rational design of substituted diarylureas: A scaffold for binding to G-quadruplex motifs. *J Med Chem* **2008**, *51*, 7751–7767, doi:10.1021/jm801245v.
17. Hernández-Núñez, E.; Tlahuext, H.; Moo-Puc, R.; Torres-Gómez, H.; Reyes-Martínez, R.; Cedillo-Rivera, R.; Nava-Zuazo, C.; Navarrete-Vazquez, G. Synthesis and in vitro trichomonicidal, giardicidal and amebicidal activity of *N*-acetamide(sulfonamide)-2-methyl-4-nitro-1*H*-imidazoles. *Eur J Med Chem* **2009**, *44*, 2975–2984, doi:10.1016/j.ejmech.2009.01.005.
18. de Sousa Luis, J.A.; da Silva Souza, H.D.; Lira, B.F.; da Silva Alves, F.; de Athayde-Filho, P.F.; de Souza Lima, T.K.; Rocha, J.C.; Mendonça Junior, F.J.B.; Scotti, L.; Scotti, M.T. Combined structure- and ligand-based

virtual screening aiding discovery of selenoglycolicamides as potential multitarget agents against Leishmania species. *J Mol Struct* **2019**, *1198*, 126872, doi:10.1016/j.molstruc.2019.126872.
19. Wise, J.G.; Nanayakkara, A.K.; Aljowni, M.; Chen, G.; De Oliveira, M.C.; Ammerman, L.; Olengue, K.; Lippert, A.R.; Vogel, P.D. Optimizing targeted inhibitors of P-glycoprotein using computational and structure-guided approaches. *J Med Chem* **2019**, *62*, 10645–10663, doi:10.1021/acs.jmedchem.9b00966.
20. Tang, R.-Y.; Li, G.; Yu, J.-Q. Conformation-induced remote meta -C–H activation of amines. *Nature* **2014**, *507*, 215–220, doi:10.1038/nature12963.
21. Venkov, A.P.; Lukanov, L.K. New modification of the intramolecular α-amidoalkylation for the synthesis of 2-acyl-1,2,3,4-tetrahydroisoquinolines. *Synthesis* **1989**, *1989*, 59–61, doi:10.1055/s-1989-27153.
